# DNA methylation analysis reveals epimutation hotspots in patients with dilated cardiomyopathy-associated laminopathies

**DOI:** 10.1186/s13148-021-01127-0

**Published:** 2021-07-10

**Authors:** Julien L. P. Morival, Halida P. Widyastuti, Cecilia H. H. Nguyen, Michael V. Zaragoza, Timothy L. Downing

**Affiliations:** 1 Department of Biomedical Engineering and The Edwards Lifesciences Center for Advanced Cardiovascular Technology, University of California Irvine, 2408 Engineering III, Irvine, CA 92697 USA; 2 UCI Cardiogenomics Program, Department of Pediatrics, Division of Genetics and Genomics and Department of Biological Chemistry, University of California Irvine, 2042 Hewitt Hall, Irvine, CA 92697 USA; 3 NSF-Simons Center for Multiscale Cell Fate Research, University of California Irvine, Irvine, CA USA; 4 Center for Complex Biological Systems, University of California Irvine, Irvine, CA USA; 5 Department of Microbiology and Molecular Genetics, University of California Irvine, Irvine, CA USA

**Keywords:** DNA methylation, Lamin A/C, Dilated cardiomyopathy, Brachydactyly

## Abstract

**Background:**

Mutations in *LMNA*, encoding lamin A/C, lead to a variety of diseases known as laminopathies including dilated cardiomyopathy (DCM) and skeletal abnormalities. Though previous studies have investigated the dysregulation of gene expression in cells from patients with DCM, the role of epigenetic (gene regulatory) mechanisms, such as DNA methylation, has not been thoroughly investigated. Furthermore, the impact of family-specific *LMNA* mutations on DNA methylation is unknown. Here, we performed reduced representation bisulfite sequencing on ten pairs of fibroblasts and their induced pluripotent stem cell (iPSC) derivatives from two families with DCM due to distinct *LMNA* mutations, one of which also induces brachydactyly.

**Results:**

Family-specific differentially methylated regions (DMRs) were identified by comparing the DNA methylation landscape of patient and control samples. Fibroblast DMRs were found to enrich for distal regulatory features and transcriptionally repressed chromatin and to associate with genes related to phenotypes found in tissues affected by laminopathies. These DMRs, in combination with transcriptome-wide expression data and lamina-associated domain (LAD) organization, revealed the presence of inter-family epimutation hotspots near differentially expressed genes, most of which were located outside LADs redistributed in *LMNA*-related DCM. Comparison of DMRs found in fibroblasts and iPSCs identified regions where epimutations were persistent across both cell types. Finally, a network of aberrantly methylated disease-associated genes revealed a potential molecular link between pathways involved in bone and heart development.

**Conclusions:**

Our results identified both shared and mutation-specific laminopathy epimutation landscapes that were consistent with lamin A/C mutation-mediated epigenetic aberrancies that arose in somatic and early developmental cell stages.

**Supplementary Information:**

The online version contains supplementary material available at 10.1186/s13148-021-01127-0.

## Background

Mutations in the *LMNA* gene, which codes for the lamin A/C proteins, cause a variety of diseases including premature aging, muscular dystrophy, lipodystrophy, and bone abnormities. Cardiac disease such as dilated cardiomyopathy (DCM), however, remains the most common type among the *LMNA*-related diseases, called laminopathies [1]. While the cardiac disease typically presents in adulthood, other laminopathy-associated phenotypes, such as facial and digital bone abnormalities, are congenital and indicative of disease mechanisms occurring early in development.

The intermediate filaments lamin A/C line the inner membrane of the nuclear envelope and are essential in providing structure to the nucleus, while simultaneously linking the chromatin to the cytoskeleton [2]. DNA regions associated with lamins at the periphery of the nucleus, termed lamina-associated domains (LADs), have previously been shown to be part of heterochromatin, the condensed region of chromatin where gene expression is silenced [3]. These structural associations, however, are disrupted in cases of mutated *LMNA*, leading to nuclear blebbing and subsequently nuclear envelope rupture [4]. Together, these events lead to DNA damage [5], as well as altered gene expression and chromatin organization [6].

Due to this chromatin structure disturbance, it is unsurprising that *LMNA* mutation studies related to DCM have focused on identifying differences in gene expression and LADs in diseased cells [7, 8]. However, the role of DNA methylation, which works in conjunction with the chromatin to control gene expression, has not been thoroughly investigated in the context of *LMNA* mutations. A recent study examined the impact of DNA methylation in heart tissue from patients with DCM [8]. This study concluded that altered CpG methylation, in combination with LAD redistribution and dysregulated gene expression, plays a key role in DCM pathogenesis. Although this study further solidifies the potential role of DNA methylation in the context of DCM, the individual impact of each family-specific *LMNA* mutation was not considered. Taking into account, the specific mutation remains important since laminopathies arise in a large variety of tissue types and tissue abnormalities often appear in a mutation-specific fashion [1, 9, 10]. Furthermore, it was previously shown that methylation levels varied at the promoter of laminopathy-related genes in cells with two distinct *LMNA* mutations [11].

The objective of this study is to understand the epigenetic mechanisms that play a role in both early developmental phenotypes (brachydactyly) and late-onset conditions (DCM) that arise due to *LMNA* mutations. To do so, we studied two families with distinct *LMNA* mutations, a heterozygous splice-site mutation causing DCM [12] and a heterozygous missense mutation displaying both DCM and brachydactyly, similar to heart–hand syndrome (HHS) IV [13]. We performed reduced representation bisulfite sequencing (RRBS) to explore the regulatory consequences of accumulating DNA methylation aberrancies [14] in patient-biopsied fibroblasts and their induced pluripotent stem cell (iPSCs) derivatives as in vitro culture models for somatic (adult) and early developmental stages, respectively. We identified family-specific differentially methylated regions (DMRs) in each cell type and performed integrative genomic analysis to compare these DMRs with changes in gene expression and LAD organization observed in previous studies in DCM patient tissues [7, 8]. We hypothesize that we will identify differential DNA methylation predictive of a disease mechanism both shared across laminopathies and unique to each family mutation.

## Results

### Genome-wide DNA methylation analysis within family-specific primary fibroblasts and iPSCs

**Fig. 1:**
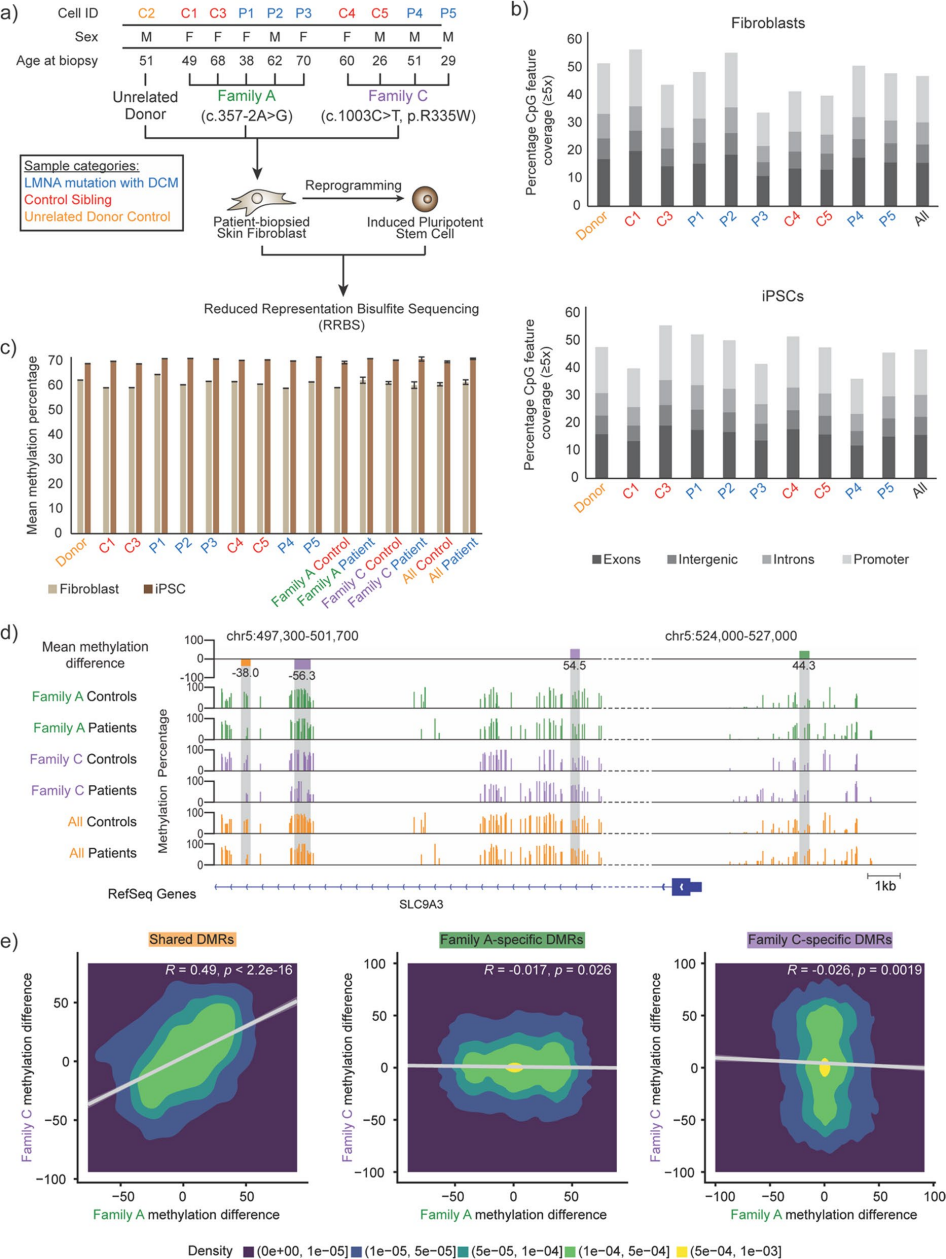
Characterization of DNA methylation in *LMNA*-mutant fibroblasts and iPSCs. **a** Schematic representation of the experimental setup. Cluster branches indicate groups of samples by family. **b** Stacked bar plot showing the percentage of CpGs (≥ 5 × depth) in a particular feature (Exon, Intergenic, Introns, Promoter from bottom to top) for all samples individually and merged in fibroblast (top) and iPSC (bottom). **c** Bar plot displaying mean genome-wide DNA methylation percentage using CpGs (≥ 5 × depth) across all samples individually and merged by groups in fibroblasts (tan) and iPSCs (brown). **d** Example of regions with CpG methylation differences between patient and control fibroblasts. Top, Genome browser track (chr5:497,300–501,700 and chr5:524,000–527,000) displaying DMRs based on mean methylation differences (patient minus control) by group (Family A-specific—green, Family C-specific—purple, Shared—orange). Middle, Methylation levels for patient and control samples by group. Gray regions reflect the location of DMRs from the top track. Bottom, Depiction of RefSeq gene annotation. **e** 2D density plots of CpG methylation difference (patient minus control) in fibroblasts from Family C (*y*-axis) or Family A (*x*-axis) at Shared, Family A-specific, and Family C-specific DMRs

To investigate the effect of *LMNA* mutations on the DNA methylation landscape, RRBS was performed on primary skin fibroblasts (and their iPSC derivatives) obtained from two families harboring unique *LMNA* mutations, and an additional unaffected (and unrelated) donor control cell line (Fig. 1a). After filtering, we captured an average of 2.2 million CpGs per sample in both cell types (Additional file 1: Table S1), of which 1,539,576 (62.2–73.2% of total CpGs) and 1,418,269 (58.2–62.9% of total CpGs) overlapped all samples in fibroblasts and iPSCs, respectively (Additional file 1: Figure S1A). Filtered CpGs represented a large portion of CpGs found in exons (13.7–20.0%) and promoters (12.1–20.5%) in fibroblasts, and in iPSCs (12.0–19.2% and 12.8–19.9%, respectively) (Fig. 1b). This represented a coverage of approximately half of all promoters in both fibroblasts and iPSCs (Additional file 1: Table S2). The relative distribution of CpGs captured in exon, intergenic, intron, and promoter was similar within each sample, in both cell types. These results agree with previous reports that RRBS captures about 2.8 million CpGs, within 60% of promoters [15, 16].

Globally, average methylation levels of controls (60.6 ± 0.6 in fibroblast and 69.7 ± 0.3% in iPSC) and patients (61.42 ± 0.9% in fibroblast and 70.9 ± 0.6% in iPSC) did not vary between the two groups (Fig. 1c). This observation was consistent when separated by family. At the single CpG level, however, we observed differences between patient and control sample methylation levels in both fibroblast and iPSCs (Additional file 1: Figure S1B), with the largest differences observed at CpGs with intermediate methylation (30–60%) in controls. To obtain a regional view of how methylation patterns change in patient samples compared to unaffected controls, we focused on differences in methylation levels over sections of the genome rather than individual CpGs. Interestingly, some differences in methylation, in the fibroblast genome for example, appeared to be shared across both families (Fig. 1d). In contrast, other methylation differences were unique to one family, with little differences seen across samples in the other family. Due to the presence of distinct regional methylation difference between patient samples and unaffected controls, we focused our analysis on DMR tiles (Additional file 1: Figure S2), classified as “Shared” (Fig. 1d, e, orange shaded region), “Family A-specific” (green shaded region), or “Family C-specific” (purple shaded region). Methylation differences of Family A and Family C samples were confirmed to significantly correlate genome-wide at shared DMRs (Fig. 1e, left panel, Pearson correlation: *R* = 0.49, *p* < 2.2 × 10^–16^), while no positive correlation was observed at Family-specific DMRs (Pearson correlation: *R* = − 0.017, *p* = 0.026 for Family A; *R* = − 0.026, *p* = 0.0019 for Family C).

### Family-specific epigenetic signatures dominate DMR landscape in fibroblasts

**Fig. 2:**
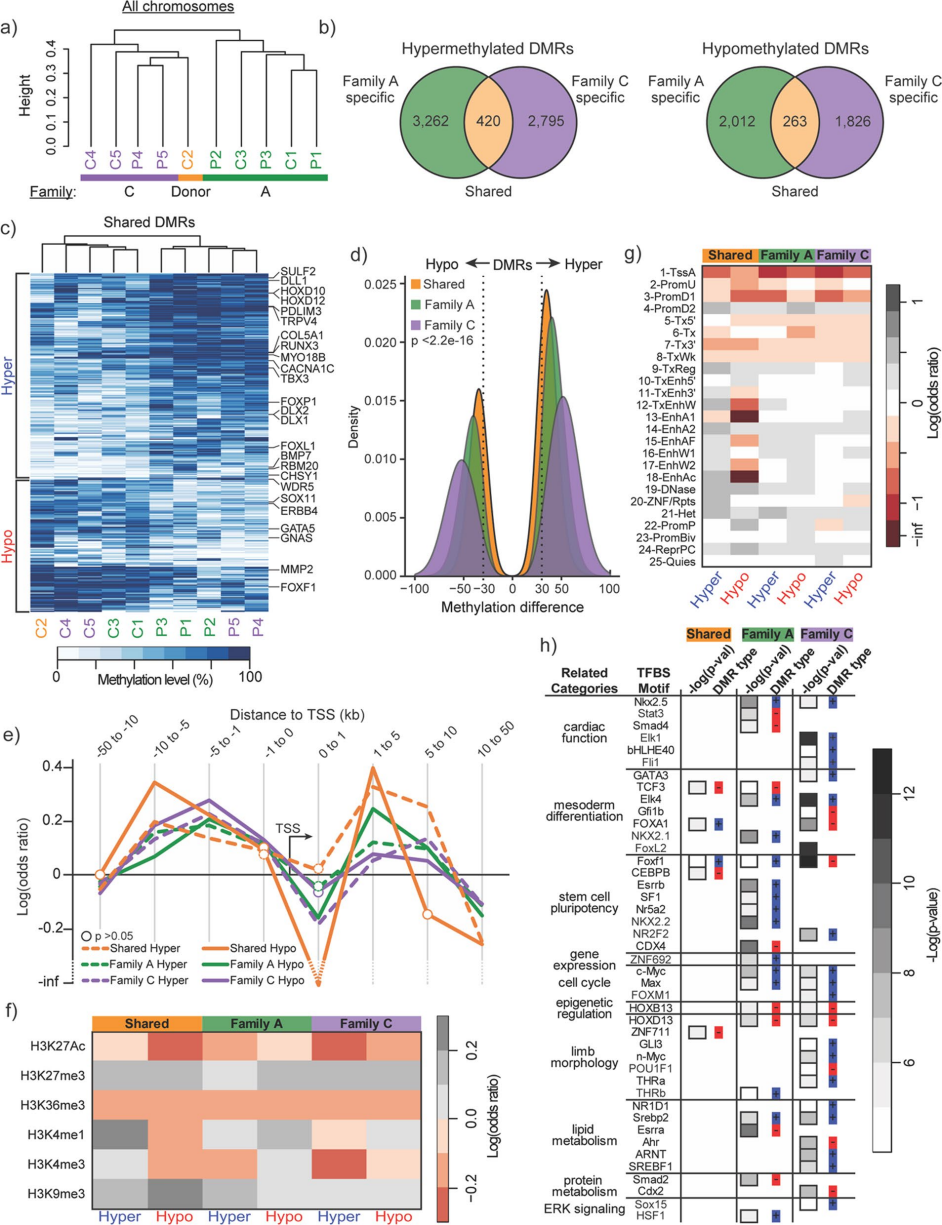
Hypermethylated and hypomethylated DMRs localize at distal regulatory features and transcriptionally repressed chromatin in fibroblasts. **a** Hierarchical clustering of all fibroblast samples by genome-wide DNA methylation. Colors represent family groupings. **b** Venn diagrams showing the number of DMRs captured by group for both hypermethylated and hypomethylated DMRs. Orange regions denote “Shared DMRs,” green regions denote “Family A-specific DMRs,” and purple regions denote “Family C-specific DMRs.” **c** Top, Hierarchical clustering of all samples by shared DMR methylation. Bottom, Heatmap of average CpG (≥ 5 × depth) methylation percentage across shared DMRs for each individual sample. Genes associated with heart and skeletal system development are shown next to the associated DMR. **d** Density plot of mean methylation difference (patient minus control) within DMRs by group. Overall Kruskal–Wallis test *p*-value is displayed. **e** Line plot of log odds ratio of the likelihood of CpGs to fall within a hypermethylated (“Hyper”) or hypomethylated (“Hypo”) DMR and a given range of genomic distance away from a gene’s TSS. Open circles designate log odd ratios that were non-significant (*p*-value > 0.05) by Fisher’s exact test. **f** Heatmap showing the log odds ratio of a CpG falling within both a DMR group and a given histone modification. **g** Heatmap showing the log odds ratio of a CpG falling within both a DMR group and one of 25 ChromHMM annotated genomic regions. **h** Table highlighting TFBS motifs enriched in shared, Family A, and Family C DMRs, grouped by TF-related categories. Heatmap reports the degree of statistical significance for TFBS motif enrichment. Results were categorized as hypomethylated (red) or hypermethylated (blue) according to the type of DMR associated with a particular TFBS motif

To characterize family-specific and shared DNA methylation differences between patient and control samples, we first focused on data from patient-biopsied fibroblasts only. Despite no differences in global methylation levels between families (Fig. 1c), hierarchical clustering of samples based on all DNA methylation data showed that samples tended to group according to family (Fig. 2a). Clustering was also performed on samples following removal of sex chromosomes X and Y, in order to identify possible sex biases. Despite clusters no longer segregating by family (Additional file 1: Figure S3A), the average Pearson correlation coefficient of genome-wide methylation data between samples was higher when compared between samples of the same family than when compared across families (Additional file 1: Figure S3B), indicating that genome-wide methylation signatures were more dependent on family than sex. Furthermore, DMRs in sex chromosomes made up only 0.76–2.63% of total DMRs generated for each category (Fig. 2b, Additional file 1: Figure S3C).

By performing methylation comparisons between patient and control samples within the same family, we posited that disease-specific patterns of differential methylation would more strongly emerge from our analyses, while normalizing for family-specific methylation pattern biases. We therefore focused the rest of our analyses on “shared” (Fig. 2b, orange shaded region), “Family A-specific” (green shaded region), or “Family C-specific” (purple shaded region) DMRs. These three groupings were replicated through hierarchical clustering based on methylation at DMR locations (Additional file 1: Figure S4). While clustering based on shared DMR methylation showed a clear separation between patient and control samples, family-specific clusters still emerged from within each patient and control sub-cluster (Fig. 2c). This evidence, together with the identification of a relatively low number of shared DMRs overall (Fig. 2b), shows that family-specific changes dominated our DMR analysis. Furthermore, we noted that the absolute median methylation difference across DMR tiles was significantly higher across family-specific comparisons (41.60 for Family A, and 52.63 for Family C) relative to DMRs obtained from our shared comparison (34.10) (Fig. 2d, Kruskal–Wallis test: *p*-value < 2.2 × 10^–16^). These findings indicated that epimutations that arise in DCM patients occur largely in a family-specific manner.

### Fibroblast DMRs associate with distal regulatory features and transcriptionally repressed chromatin

To investigate the potential regulatory impact of the DMRs identified, we used the Genomic Regions Enrichment of Annotations Tool (GREAT) [17] to identify genes that our DMRs may be regulating, both proximally and distally. Shared DMRs, despite their low frequency, revealed an association to 62 genes included in heart (e.g., *GATA5*, *FOXL1*, *TBX3*, *MYO18B*, *CACNA1C*, *BMP7*) and skeletal system (e.g., *HOXD10*, *HOXD12, RUNX3*) development GO terms (Fig. 2c, full list shown in Additional file 1: Table S3). To examine the potential regulatory impact of methylation on these DMR-associated genes, we performed an odds ratio (OR) analysis to determine the likelihood of CpGs falling within each of the three DMR groups and within a given genomic distance of a gene’s transcriptional start site (TSS). This analysis revealed that CpGs within DMRs were generally more significantly likely to fall within genomic locations 1–10 Kb upstream of a given gene’s TSS and, more proximally, between 1 and 5 kb downstream of the TSS (Fig. 2e, Fisher’s exact test: *p*-value ≤ 0.05, unless specified as non-significant).

The tendency of DMR-overlapping CpGs to fall distally to TSSs, beyond ± 1 kb, suggested that disease-associated changes in methylation could exist within diverse chromatin context that lie largely outside of promoters (which generally showed an odds ratio close to 1) and potentially within distal gene regulatory elements. To explore this, we performed a similar odds ratio analysis across a broader chromatin context (Fig. 2f, g, Fisher’s exact test: *p*-value ≤ 0.05, unless specified as non-significant in Additional file 1: Tables S4 and S5), to infer any potential role that aberrant methylation patterns might have on gene regulation in patient cells. Interestingly, an analysis based on CpG overlap within fibroblast-specific histone modification landscapes (rather than distance from TSS) revealed that CpGs within hypermethylated DMRs obtained from our shared category showed a strong association (logOR = 0.24; *p*-value = 2.08 × 10^–20^) with regions marked by histone 3 lysine 4 mono-methylation (H3K4me1), a histone mark traditionally enriched at enhancers [18, 19] (Fig. 2f). This was in stark contrast to CpGs within hypermethylated Family C DMRs, which displayed a protective effect with respect to H3K4me1 marks (logOR = − 0.05; *p*-value = 3.2 × 10^–5^). Conversely, Family A hypermethylated (logOR = 0.04; *p*-value = 2.71 × 10^–5^) and hypomethylated DMRs (logOR = 0.12; *p*-value = 4.61 × 10^–24^) both showed a slightly stronger association with this histone modification. A similar analysis which included the removal of sex chromosomes showed similar histone modification enrichment (Additional file 1: Figure S3D) to those previously mentioned.

We next took a more focused approach toward understanding the relationship between the occurrence of CpGs in DMRs and functionally annotated genomic regions, as assigned (computationally) by ChromHMM [20, 21]. These results revealed that all of our DMR categories showed a significant increased association with at least one subtype of enhancer annotation, including those functionally characterized as weak (annotation 16–18), strong (annotation 13–15) or transcribed (annotation 10–12). (Fig. 2g, Fisher’s exact test: *p*-value ≤ 0.05, unless specified as non-significant in Additional file 1: Table S5). Additionally, we saw a general negative association with promoter annotations (annotation 2–3, Fisher’s exact test: *p*-value ≤ 0.02); however, we did observe strong associations with “downstream promoter elements” (annotation 4, Fisher’s exact test: *p*-value ≤ 0.04), which likely coincide with the increased association of DMRs at genomic distances 1–5 kb downstream of gene TSSs that we observed previously (Fig. 2e). Removal of sex chromosomes did not affect the results above for our ChromHMM analysis (Additional file 1: Figure S3E).

We also observed that DMRs showed a strong likelihood to fall within histone modifications—H3K27me3 and H3K9me3 (Fig. 2f, Fisher’s exact test: *p*-value ≤ 2.2 × 10^–5^)—and functional genomic annotations—heterochromatin (annotation 21) and polycomb repression (annotation 24) ChromHMM annotations (Fig. 2g, Fisher’s exact test: *p*-value ≤ 0.03)—associated with gene repression. This was particularly interesting given that LADs, which are disrupted due to numerous *LMNA* mutations [8, 22, 23], typically co-localize to the nuclear periphery along with heterochromatic regions of DNA and also marked by H3K9m3 and H3K27me3 [3].

We next wanted to investigate whether DMR locations co-localized with certain classes of regulatory factor binding sites (TFBS). This could reveal important molecular targets within key signaling pathways that might be impacted by family-specific epimutations. We performed TFBS motif enrichment analysis in our DMRs using HOMER [24], focusing on TFBS motifs enriched only in either hypo- or hypermethylated DMRs. Few TFBS motifs were enriched within shared DMRs; however, these motifs were involved in mesoderm differentiation (e.g., *TCF3*, *FOXA1*) and stem cell pluripotency (e.g., *Foxf1* and *CEBPB*) (Fig. 2h, full list shown in Additional file 1: Table S6). Conversely, family-specific DMRs enriched for TFBS motifs of transcription factors (TFs) previously shown to be implicated in multiple categories relevant to laminopathies (cardiac function, limb morphology, lipid metabolism, mesoderm differentiation). The tendency of Family C DMRs to enrich for several TFBS motifs associated with limb morphology was particularly interesting given this family’s presentation of a brachydactyly phenotype. In general, DMRs related to the enriched motifs were largely hypermethylated, though this could be due to the larger amount of hypermethylated DMRs present in fibroblasts.

### Fibroblast DMR-associated genes enrich for family-specific disease ontologies

**Fig. 3:**
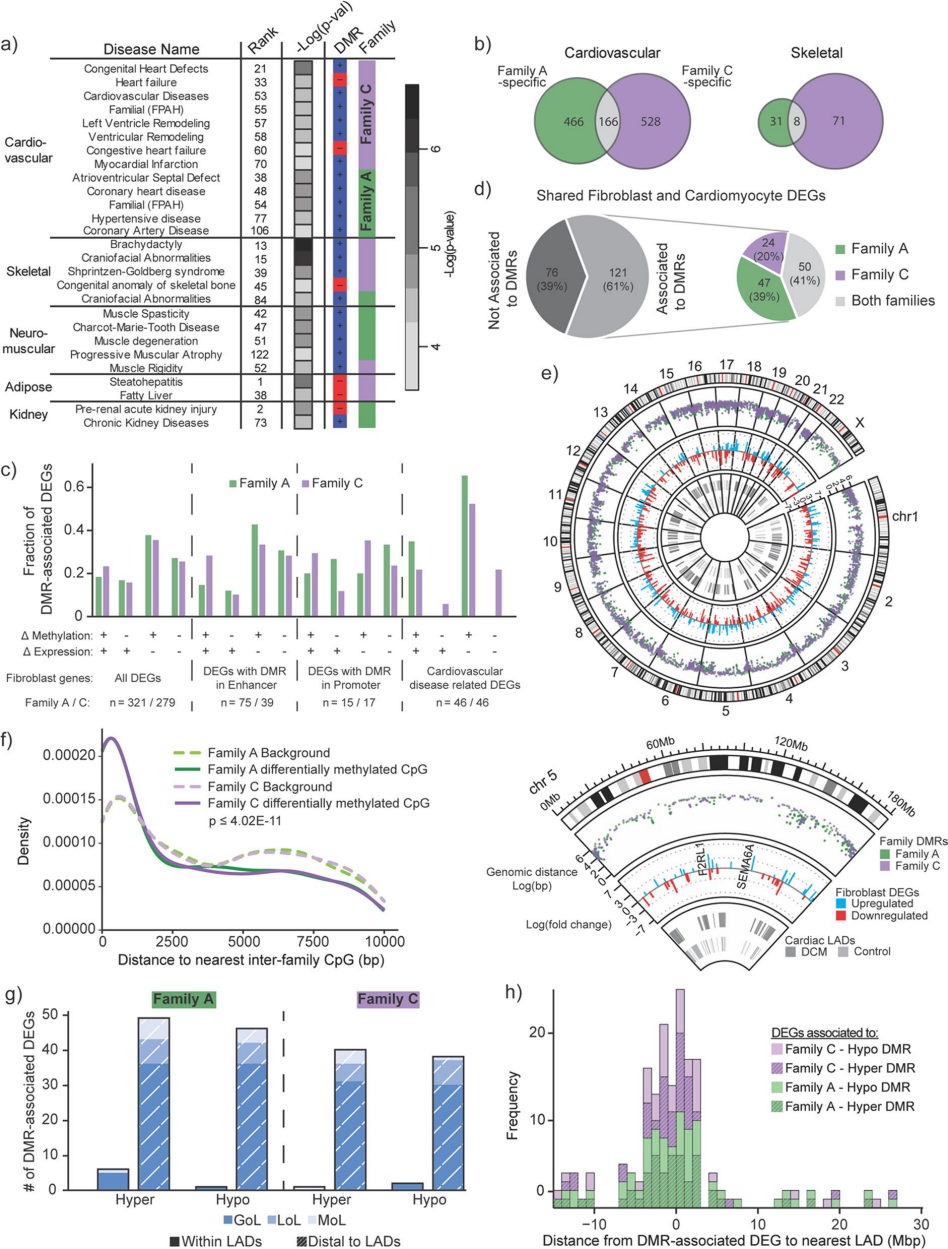
DMRs associate to dysregulated and disease-relevant genes near redistributed LADs. **a** Disease ontology terms enriched in DMRs, grouped by disease type. Heatmap reports the degree of statistical significance for enrichment. Results were categorized as hypomethylated (red) or hypermethylated (blue) by type of DMR associated with a particular disease. **b** Number of genes in cardiovascular and skeletal disease associated with Family A-specific and Family C-specific DMRs. **c** Top, Fraction of DMR-associated fibroblast DEGs present in one of four combinatorial groups of differential methylation (Δ Methylation) and differential gene expression (Δ Expression). Middle, (+) indicate patient > control, while (−) indicate patient < control for both differential methylation and gene expression. Bottom, Category of fibroblast DEGs and number of DEGs by family (Family A/Family C). **d** Number and percentage of DEGs shared between fibroblast and cardiac tissue associated with DMRs in Family A only, Family C only, or both. **e** Circos map of the genome (Top) and zoomed in chromosome 5 (Bottom). Outer to inner rings represent the following: Track I—genomic distance (log 10) between DMRs within Family A or Family C. Track II—fold change (log 2) of fibroblast DEGs, highlighting two genes found within the top 10 most differentially expressed. Track III—location of LADs in cardiomyocytes from either *LMNA*-related DCM or control samples from prior study [8]. **f** Density of genomic distance to the nearest inter-family CpG for differentially methylated CpGs and a random sample of CpGs. Wilcoxon rank sum test *p*-value is displayed. **g** Number of DEGs shared between fibroblast and cardiac tissue associated with DMRs in Family A or Family C falling within or distal to redistributed LADs (Gain of LAD (GoL), Loss of LAD (LoL), or Maintenance of LAD (MoL)). **h** Stacked histogram of the distance between DMR-associated DEGs, shared between fibroblast and cardiac tissue, and the nearest redistributed LAD

Due to the enrichment of TFBS motifs associated with pathways critical for tissue functions commonly disrupted in laminopathy diseases, we decided to investigate if shared and/or family-specific DMRs enriched for certain disease ontologies (Additional file 1: Table S7). We performed disease ontology enrichment on genes associated with either hypo- or hypermethylated DMR contexts. The large presence of disease ontology terms represented by genes associated with hypermethylated DMRs (Fig. 3a) further demonstrated the bias toward this type of DMR. We also found that Family A and C DMRs showed enriched association with several laminopathy disease categories, while shared DMRs showed no enrichment within these categories (Fig. 3a). This observation corroborated the low number of TFBS motifs that were associated with categories related to laminopathy-impacted tissues (“cardiac development”, “limb development”, “lipid metabolism”) that we noted previously (Fig. 2h). Both families equally enriched for a variety of cardiovascular diseases, including both cardiac remodeling and hypertensive diseases, which supported the DCM phenotype observed in both families. Despite patients not exhibiting hypertensive disease, both sets of family-specific DMRs enriched for this phenotype, which has been shown to lead to excessive remodeling of the myocardium, resulting in the development of DCM [25]. Similar to our motif enrichment, we also observed a strong enrichment for diseases associated with skeletal malformations in Family C DMRs. Indeed, brachydactyly, which Family C patients exhibit, was the most enriched laminopathy-related ontology associated with our Family C DMR dataset. Family A DMRs instead favored diseases related to neuro-muscular phenotypes. Surprisingly, we also observed the presence of kidney-related disease terms in genes associated with Family A DMRs. Although not widely recognized as a form of laminopathy, several studies have documented the occurrence of kidney-related diseases in patients with *LMNA* mutation-induced lipodystrophy or DCM [26, 27]. A large majority of the remaining disease ontologies (Additional file 1: Table S7) were found to be involved in either cancer (21%) or nervous system disorders/abnormalities (50%). The documented low levels of lamins in several types of cancers [28] and the known involvement of neurodegeneration [29] and neuropathies [30] in laminopathies could account for some of these observations.

### Genes dysregulated in both fibroblast and DCM cardiac tissues associate with DMRs from both families and LADs

The lack of enrichment for diseases related to tissues affect by laminopathies in genes associated with shared DMRs led us to focus on family-specific DMRs only. Given that both family-specific DMR sets were enriched for cardiovascular and skeletal disease ontology categories, we evaluated for inter-family gene overlap within each of the corresponding disease-associated gene sets. Unexpectedly, we found no overlap for the majority of these genes including those in the cardiovascular category despite both families exhibiting DCM (Fig. 3b).

To examine this more thoroughly, we first compared our DMR data with the list of differentially expressed genes (DEGs) between patient and their unaffected controls previously obtained from transcriptome-wide expression data from Family A fibroblasts [7] (Fig. 3c). To ascertain the potential role of aberrant DNA methylation on differential expression, we compared the direction of methylation change within DMRs to the direction of expression change for associated DEGs. Genome-wide DMRs associated with both families were weakly inversely correlated (54.5%, Quadrant Count Ratio (QCR) = − 0.09 for Family A and 51.2%, QCR = − 0.03 for Family C) with expression changes (i.e., higher methylation level in patients compared to controls (+) was associated with lower gene expression (−)) (Fig. 3c). Notably, analysis of DEGs present within our previously identified cardiovascular disease-related gene list (Fig. 3b) also showed an inverse correlation between methylation and gene expression though more pronounced in both families (65.2%, QCR = − 0.30 for Family A and 56.5%, QCR = − 0.13 for Family C). Most of these correlations were the result of hypermethylation association to decreased expression. This observation corroborates our previous observations of hypermethylation also being associated with disease-related genes (Fig. 3a).

When broken down into DEGs associated with DMRs located within gene enhancers, we noted that this bias was also present. However, we did observe more DEGs were inversely correlated with DMR methylation changes in Family A (QCR = − 0.09), unlike in Family C (QCR = 0.13). In the promoter context, however, DMRs associated with DEGs did not show any negative trends with expression changes (QCR = 0.07 for Family A and QCR = 0.06 for Family C). These findings are consistent with a more important regulatory function for enhancer-located DMRs in Family A compared to Family C, and a lack of association in both families for DMRs in upstream promoters (Fig. 2g), also observed in a prior study on *LMNA*-related DCM cardiac tissues [8].

To relate our DMR data to DEGs observed within a more physiologically relevant context, we identified DEGs found in both our patient fibroblasts and within DCM patient cardiac tissues from a prior study [8]. Interestingly, 61% of the 197 conserved DEGs were associated with a DMR from at least one of the families (Fig. 3d). Remarkably, despite the lack of inter-family overlap seen for disease-related genes (Fig. 3b), 41% of DEGs in this category were found to associate with at least one DMR from both families. Given this overlap, we wondered if inter-family DMRs occurred in close genomic proximity more broadly. To explore this, we compared the density distributions of CpG proximity in DMRs for each family and random background (Fig. 3f, Wilcoxon rank sum test: *p*-value ≤ 4.02 × 10^–11^ for both families). We found that differentially methylated CpGs (DMCpGs) indeed showed a greater density bias toward smaller inter-family distances (median for Family A: 2192.5 bp, Family C: 2036.5 bp) compared to the random background (median for Family A: 3640 bp, Family C: 3645 bp), up until about 1450 bp. This proximity between Family A and C DMRs was also observed in our circos and rainfall plot analysis (Fig. 3e, Additional file 1: Figure S5).

Given these results along with our previous observations that DMRs, in general, tended to associate with epigenomic features that co-localize to the nuclear periphery (Fig. 2f, g), we next analyzed the proximity of DMRs associated with conserved DEGs (between fibroblasts and cardiac tissues) to LADs known to be dynamic (or redistributed) in *LMNA*-related DCM [8] (Fig. 3e). In addition to two previously defined domain redistribution categories [8], Gain of LAD (GoL) and Loss of LAD (LoL), genomic regions were also assigned to Maintenance of LAD (MoL). Of the DMR-associated DEGs found in both fibroblasts and cardiac tissues, we found that only a small fraction fell directly within a redistributed LAD (0–6.2% for GoLs, and 0% for LoLs) or MoLs (0–2.1%), comparable to those previously observed in DCM tissues [8]. The remainder of the DMR-associated DEGs were mostly distal to GoLs (73.5–78.9%) (Fig. 3g). Moreover, identified DMR-associated DEGs were found be significantly more likely to fall within 2Mbp of their closest redistributed LAD (Fig. 3h) than outside of that range (logOR = 0.50, *p* = 1.31 × 10^–7^). Interestingly, chromosome 19 did not contain any conserved DEGs distal to redistributed LADs (Additional file 1: Figure S6).

### Reprogramming reveals epigenetic hotspots for aberrant methylation during early development

**Fig. 4:**
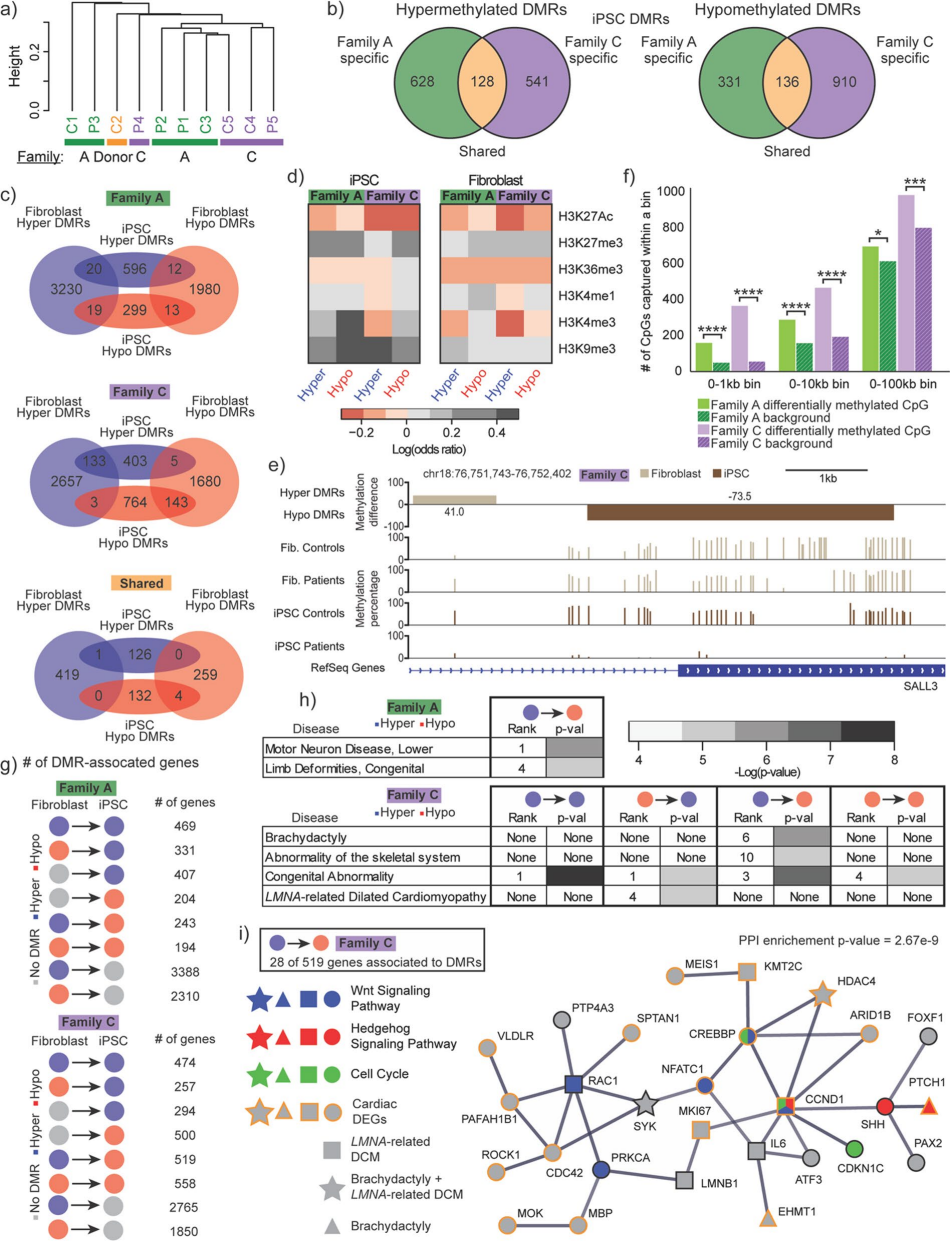
DMRs in iPSCs reveal tissue-persistent epimutation hotspots at developmentally and laminopathy relevant genes. **a** Hierarchical clustering of iPSC samples by genome-wide DNA methylation. Colors represent family groups. **b** Venn diagram showing the number of DMRs captured by group for both hypermethylated and hypomethylated DMRs. Orange regions denote “Shared DMRs,” green regions denote “Family A-specific DMRs,” and purple regions denote “Family C-specific DMRs.” **c** Number of DMRs captured within fibroblast and iPSC samples for each grouping for hypermethylated and hypomethylated DMRs. **d** Log odds ratio of a CpG falling within both a DMR group and a given histone modification in iPSC and fibroblast. **e** Example of Family C DMR proximity in both cell types. Top, Genome browser track displaying DMRs based on mean methylation differences (patient minus control) in fibroblasts and iPSCs. Middle, Methylation levels for patient and control samples for each cell type. Bottom, Depiction of RefSeq gene annotation. **f** Number of either differentially methylated CpGs or randomly sampled CpGs in iPSC that fell within a range of genomic distances from their closest neighboring fibroblast CpG in the same family; Fisher’s exact test: *p ≤ 0.05; ***p ≤ 0.001; ****p ≤ 0.0001 g Diagram depicting the number of genes associated with DMRs falling within one of eight categories of DMR methylation patterns in fibroblast and iPSCs. **h** Table highlighting laminopathy-related disease ontologies enriched in DMRs grouped by fibroblast and iPSC DMR state (hyper- or hypomethylated). Heatmap reports the degree of statistical significance for disease enrichment. **i** Protein–protein interaction (PPI) network of 28 genes associated with Family C-specific DMRs (hypermethylated in fibroblasts and hypomethylated in iPSCs) and either *LMNA*-related dilated cardiomyopathy (DCM), congenital abnormality, or both. Pathway enrichment and disease association are denoted by color and shape, respectively. Orange node borders indicate that the gene is differentially expressed in cardiac tissue (cardiac DEG)

Given that patients in Family C presented with developmental abnormalities in bone formation (brachydactyly), we wanted to see if our in vitro cell system could be used to better understand the influence of DNA methylation epimutations in the early stages of development. We therefore performed similar studies in iPSC, as an early developmental model of LMNA mutations. Unlike in fibroblasts, hierarchical clustering of iPSC samples based on DNA methylation from all chromosomes (Fig. 4a) or autosomal chromosomes only (Additional file 1: Figure S3F) did not cluster according to family. This confirmed our expectation that reprograming would lead to massive epigenetic remodeling and resetting (at least partially) of somatic methylation patterns that might have arose due to family-specific conditions [31–33]. Despite this global change in DNA methylation levels (Fig. 1d), we still identified DMRs in patient iPSCs across each category (Fig. 4b). However, the number of DMRs found in iPSCs (2674 DMRs) was still only ~ 1/4 of the number found in fibroblast (10,578 DMRs). Direct overlap between fibroblast and iPSC DMRs was greatest in Family C by almost threefold (19.6% compared to 6.7% for Family A and 1.9% for our shared category) (Fig. 4c). In addition to the greatest amount of intercell-type DMR overlap, Family C had the largest fraction (0.97 versus 0.52 for Family A) of overlapped DMRs with conserved directionality (hyper or hypomethylated).

We also found that iPSC DMRs varied in their association to histone modifications compared to their fibroblast counterparts (Fig. 4d, Fisher’s exact test: *p*-value ≤ 0.05 unless specified as non-significant in Additional file 1: Table S8). Particularly, we saw an increased presence of iPSC DMRs in H3K9me3 and H3K27me3, further highlighting the presence of aberrant methylation in the compacted and silenced regions of chromatin. We also observed an overall increase in odds ratio at H3K4me3, a histone mark enriched at active promoters [19].

Although direct overlap of DMRs across cell types was low (Fig. 4c), we observed genomic regions where iPSC DMRs were in close proximity to fibroblast DMRs (Fig. 4e), which made us wonder if regions highly susceptible to epimutations were conserved between fibroblast and iPSC states. We therefore compared the distance between CpGs in iPSCs and their closest neighboring CpG in fibroblast for both a randomized set of CpGs and our DMCpGs (Fig. 4f 1-tailed Fisher’s exact test: *p*-values ≤ 0.05). Interestingly, compared to our randomized background, 3.3 and 6.5 times more CpGs fell within 1 kb of each other between the two cell types in Family A and Family C, respectively. This fold difference decreased in both families for bins of larger inter-CpG distances. Moreover, when we focused on genes associated with DMRs in iPSCs and fibroblasts, we found a large amount of overlap between the two gene sets (Fig. 4g). Specifically, 59.8% and 61.6% of genes that were associated with an iPSC DMR were also associated with a fibroblast DMR in Family A and Family C, respectively (Additional file 1: Figure S7). We also saw a comparable number of DMR-associated genes that switched in the methylation change direction between fibroblasts and iPSCs (e.g., hyper hypo, or hypo hyper) for both families.

Analysis of these DMR-associated genes showed enrichment for laminopathy-related disease ontologies (Fig. 4h, full list shown in Additional file 1: Table S9). Family A showed enrichment only in genes associated with DMRs hypermethylated in fibroblast and hypomethylated in iPSCs. In contrast, Family C enrichment in all categories of DMRs except those that were uniquely found in iPSCs. Most notably, genes associated with Family C DMRs hypermethylated in fibroblast but hypomethylated in iPSCs showed specific enrichment for brachydactyly, abnormality of the skeletal system, and congenital abnormality. Genes associated with Family C DMRs hypomethylated in fibroblast but hypermethylated in iPSCs enriched for *LMNA*-related DCM. All four diseases were ranked in the top 10 diseases, and, interestingly, both the skeletal disease-associated DMRs and brachydactyly phenotype were unique to Family C [13].

To gain further insight into disease mechanism in our early development model, we performed protein–protein interaction network analysis, using STRING. The list of 519 genes for Family C DMRs hypermethylated in fibroblast but hypomethylated in iPSC (Fig. 4g) was filtered for association to *LMNA*-related DCM (Concept ID: C1449563) and Congenital Abnormality (Concept ID: C0000768), both of which are phenotypes that Family C patients exhibited. The resulting STRING output included a large interaction network that included 28 genes with high confidence interactions (Fig. 4i). Of the genes associated with congenital abnormality, four genes (*HDAC4*, *PTCH1*, *EHMT1*, *SYK*) were associated with brachydactyly, according to the DisGeNET [34] database. Interestingly, *LMNB1*, which codes for one of the two types of B-type lamins and is associated with DCM [35], was present within this network. Within this network, *CCND1*, the most connected node (8 associations), was involved in three pathways (Wnt signaling, Hedgehog (Hh) signaling, and the cell cycle) found to be enriched in this gene set (Additional file 1: Table S10). Another 60.7% of the genes in this network were previously identified as DEGs in hearts from *LMNA*-related DCM patients [8], substantiating that our analysis was able to reveal a highly networked set of disease-associated genes that may be dysregulated due to methylation changes linked to *LMNA* mutations.

## Discussion

We performed a comprehensive analysis of differential DNA methylation for ten matched pairs of fibroblasts and iPSC from DCM patients in two families with distinct *LMNA* mutations and their unaffected sibling controls. Our results provide new insight into mutation-specific mechanisms that influence both common and unique aspects of phenotypic expression of laminopathies.

First, our observations suggest that aberrant DNA methylation in *LMNA*-mutated cells affects not only normally silenced regions of the genome but also previously unappreciated regulatory features such as enhancers and downstream promoters. Although large differences in methylation level were not observed from genome-wide averages in either cell type, closer inspection of the RRBS data at a regional level revealed DMRs in *LMNA*-mutant samples compared to controls. In fibroblasts, we observed an increased likelihood of finding CpGs in DMRs falling 1–5 Kb downstream of TSS and distally upstream of the gene promoter. Along with DMR association to relevant histone marks such as H3K4me1 [18, 19], this suggests that Family A DMRs serve a more important regulatory function as enhancers relative to Family C DMRs, and that neither Family DMRs had much association to upstream promoters as previously shown [8]. In contrast, the association of iPSC DMRs to H3K4me3 suggested that the regulatory mechanism most impacted by differential methylation in this cell type is at promoters. In addition, the association of fibroblast and iPSC DMRs to histone modifications related to both heterochromatin and LADs suggests that, despite each of our families showing largely unique DMR landscapes, both families experience epimutations within these normally silenced regions of the genome, which could contribute to (or be associated with) the dysregulated of genes. This concept adds to the previous observation that altered CpG methylation was associated with redistributed LADs and gene dysregulation in DCM hearts [8].

Second, our results for DMRs identified multiple epimutation hotspots in the genome across all samples that may play an important role in the expression of DCM, a common laminopathy phenotype. Several shared DMRs were notably associated with genes in close genomic proximity to one another (ex. *HOXD10* and *HOXD12*), and fibroblast DEGs associated with family-specific DMRs showed a substantial amount of inter-family overlap. These inter-family epimutation hotspots were supported with observations in fibroblasts that the distance between inter-family DMCpGs had a higher density bias at short genomic distances than a random background. Furthermore, despite shared DMRs having little to no association to TFBS motif pathways and disease ontologies related to laminopathies, a relatively larger number of DMR-associated genes related to cardiovascular disease were present in both Family A and C. Thus, the identification of these epimutation hotspots across samples from families with distinct *LMNA* mutation suggests that family-specific aberrances in DNA methylation might lead to common functional consequences in DCM.

Our findings for a common subset of laminopathy epimutations in family-specific DMRs, in conjunction with LAD redistribution, also suggest a significant role of Lamin A/C in epigenetic regulating mechanisms of laminopathy-related pathways in multiple affected tissues but insufficient to express disease phenotype. The close proximity of family-specific DMRs at epimutation hotspots and silenced chromatin could explain our observation that both sets of family-specific DMRs had overlapping DEGs, shared between fibroblast and cardiac tissue DCM samples. This commonality between the two families further extended to DMR-associated DEG localization outside of redistributed LADs. Interestingly, family-specific DMRs also both showed enrichment for disease in laminopathy-related tissues outside of those affected in patients (e.g., neuromuscular, adipose, and kidney). Family A DMRs, for example, enriched for “Charcot-Marie-Tooth disease,” known to be caused by a *LMNA* mutation [30], despite neither family having muscular dystrophy. Furthermore, a previous study of patients with DCM revealed a GO term enrichment for “lipid metabolism” in genes with transcript level correlated with their associated methylation status and LAD localization [8]. Another study on Emery–Dreifuss muscular dystrophy (EDMD) similarly suggested that nuclear envelope disorders could account for a unifying molecular model responsible for the wide range of laminopathy phenotypes [11].

In addition to a possible common laminopathic mechanism, our study identified family-specific epimutations with unique regulatory functions in chromatin remodeling, disease mechanism, and phenotypic expression. Until now, DNA methylation studies using samples from DCM patients did not consider the role for specific *LMNA* mutation in affected families [8, 36]. The individual impact of specific mutations is further highlighted by the previous observation that expression of the *LMNA* mutation responsible for familial partial lipodystrophy did not induce epigenetic alterations of myogenic loci in a human myogenic cell line unlike the *LMNA* mutation involved in EDMD [11]. In our study, the presence of divergent mutation-specific epimutations is apparent in the limited overlap of disease-related genes associated with Family A and C DMRs. Family C DMRs were particularly interesting due to the strikingly significant enrichment for disease ontology of brachydactyly, a unique phenotype in patients from Family C [13]. De novo enhancer–promoter interactions from the disruption of topology associated domains (TADs) previously were demonstrated to result in ectopic gene expression and subsequently brachydactyly [37]. The significant presence of many redistributed LADs, mostly GoLs, within 2 Mb, the maximum distance for enhancer–promoter interacting pairs [38], of DEGs associated with DMRs in Family C further supports the involvement of TAD restructuring. It is therefore conceivable that the aberrant methylation observed at enhancers is a signature remnant of disease-induced chromatin remodeling.

In iPSC samples, the presence of mutation-specific epimutations also supports a disease mechanism during early development. Despite little direct overlap between iPSC and fibroblast DMRs, Family C hypermethylated and hypomethylated DMRs were more conserved from fibroblast to iPSC than DMRs in Family A. The presence of retained epimutations further supported Family C’s involvement in the iPSC’s primed pluripotent state. Paradoxically, the subset of Family C DMRs, which reversed methylation directionality from being hypermethylated in fibroblasts to hypomethylated in iPSCs, was associated with developmental genes implicated in skeletal malformations, echoing the family’s unique brachydactyly phenotype. This suggests aberrant increases and decreases in DNA methylation in regions more susceptible to epimutations are important in disease pathogenesis.

Finally, the set of genes associated with these reversed Family C DMRs, when filtered, provided us with a particularly interesting network of protein–protein interaction that provides further involvement of the Wnt signaling pathway and cell cycle regulation in the disease mechanism of laminopathies for DCM. Despite Family C patients having skeletal involvement, our network showed a specific association also to cardiac disease in several ways. Foremost, over half of the genes identified within our network was previously identified as DEGs in hearts from DCM patients [8]. Additionally, the Wnt signaling pathway, enriched in our network, is known to be involved in heart development and disease [39, 40] and dysregulated in *LMNA*-mutated mouse models of DCM [41]. In parallel, Wnt proteins regulate the cell cycle, itself involved in cardiac development and disease [42]. Specifically, cell cycle-related GO terms previously were observed in genes associated with redistributed LADs with altered CpG methylation and differential expression in cardiac tissue from *LMNA*-related DCM patients [8, 43]. Furthermore, cell cycle progression is tightly regulated during cardiac development, with the exit of G1 phase mediated through E2F transcription of its target genes [42]. Despite not being associated with cell cycle, the expression of *LMNB1*, encoding for lamin B1, previously was shown to be regulated by E2F as part of cell cycle progression [44]. The presence of lamin B1 is especially significant in the context of iPSCs since this isoform is expressed in early embryo and differentiating cells, unlike lamin A/C which is expressed primarily in differentiated somatic cells [1]. E2F TF target genes previously were shown to be dysregulated in *LMNA*-mutated cardiomyocytes with DCM [43]. Of the dysregulated E2F target genes [43], three (*CCND1*, *CDKN1C*, *MKI67*) were identified in our network. *CCND1*’s involvement in cardiac disease is supported by its presence in both the cell cycle and Wnt [45] signaling and previous observations of upregulation in DCM [43, 46]. Interestingly, a previous study of EDMD also implicated E2F and cell cycle dysregulation as a key feature of the disease mechanism [11].

In addition to DCM, our protein–protein interaction network provides further involvement of the Hedgehog (Hh) signaling pathway and cell cycle regulation in the disease mechanism for brachydactyly. *CCND1*, as mentioned above, encodes for Cyclin D1 that also is involved in Hh [47] signaling, an important regulating pathway in limb development [48]. *SHH*, one of the three Hh proteins, has specifically been shown to be tightly regulated by a long-range enhancer region, whose disruption can lead to *SHH* dysregulation and subsequent finger malformation [48]. The relevance of our network in finger malformation was further highlighted by the presence of genes involved in brachydactyly (*HDAC4*, *PTCH1*, *EHMT1*, *SYK*). Of particular note, *HDAC4* is considered highly associated with brachydactyly (second highest gene-disease association according to the disease database DisGeNET [34]), due in part because of its direct involvement in inducing brachydactyly mental retardation syndrome (BDMR) [49, 50]. Additionally, *PTCH1* has also been previously involved in brachydactyly as part of Hh signaling [51]. Together, these results suggest that epimutations at important cell cycle genes such as *CCND1* could provide a molecular link for how both cardiovascular disease and limb malformation may be present in patients.

## Conclusions

This study describes a framework for how DMR analysis of in vitro systems can be utilized to understand how regulatory elements become misregulated in laminopathy-associated diseases. Our results add to the previous studies substantiating that DNA methylation and chromatin remodeling of LADs/TADs have a combinatorial impact on the dysregulation of genes responsible for the development of DCM. Additionally, the family-specific DMR gene associations suggest the presence of both a laminopathy-shared and a mutation-unique set of epimutations. This type of analysis may prove to be highly beneficial for identifying networks of disease-relevant genes for rare diseases such as Family C’s HHS IV, which have a limited disease-gene association database.

Still, certain limitations of this study must be considered. First, our study only had a limited number of patients and sibling controls per mutation and were not sex-diverse. This limits our ability to attain high statistical power and entirely rule out any sex bias, respectively. Additionally, our observations were made in patient skin fibroblasts and their iPSCs derivatives, neither of which are directly involved in the observed disease phenotypes. The study was performed, however, under the assumption that these more easily obtainable cell types could maintain a disease-specific epigenetic signature and thus, provide us with a powerful model to use as a foundation for future works.

Ultimately, our study highlights the potential for DNA methylation to provide new perspective on the etiology of mutation-specific laminopathies, as well as an alternative therapeutic substrate. Future studies will focus on validating the misregulation of identified genes and performing similar analyses on iPSCs-derived cardiomyocytes and osteoblasts from these *LMNA* families to confirm our findings and to identify further gene networks associated with epimutations.

## Methods

### Fibroblast and iPSC lines

Ten matched pairs of PATIENT and CONTROL fibroblasts and iPSC lines were used in this study (Fig. 1a and Additional file 1: Table S11). For the PATIENT group, dermal fibroblasts were cultured from skin biopsies obtained from five affected individuals of two *LMNA* study families (A and C) as previously reported [12, 13]. Family A includes three patients (P1, P2, and P3) heterozygous for *LMNA* splice-site (c.357-2A>G) that exhibit sick sinus syndrome and DCM leading to heart failure [12]. Family C includes two patients (P4 and P5) heterozygous for *LMNA* missense (p.Arg335Trp) mutation displaying conduction disease, DCM, and brachydactyly, similar to HHS IV [13]. For the CONTROL group, dermal fibroblasts were cultured from skin biopsies obtained from four unaffected siblings (C1, C3, C4, and C5) and from a purchased sample obtained from one healthy, unrelated “Donor” individual (C2) (CC-2511, Lonza, Basel, Switzerland). Fibroblast culture and genomic DNA (gDNA) extraction were performed as described previously [12, 13]. By Sanger sequencing of all 12 *LMNA* exons in fibroblast DNA, the presence or absence of the LMNA mutation was confirmed in all PATIENT and CONTROL lines, respectively.

To generate matched iPSC lines, the PATIENT and CONTROL fibroblasts were reprogrammed using the CytoTune-iPS 2.0 Sendai Reprogramming Kit (Life Technologies, Carlsbad, CA) that uses a replication-defective Sendai virus as vectors to introduce reprogramming factors (OCT3/4, SOX2, KLF4, c-MYC) into the host cell [52, 53]. Cryopreserved fibroblasts at passage 5 were revived for culture in 20% FBS (Sigma-Aldrich, St. Louis, MO) and DMEM (Life Technologies) at 37C and 5% CO2. At passage 7, fibroblasts were confirmed free of mycoplasma infection using MycoAlert Mycoplasma Detection Kit and Assay Control (Lonza) and plated at the appropriate density on 6-well plates two days prior to Sendai viral transduction to achieve 50–80% confluency. The cells were transduced (Day 0) using the calculated volumes of each virus to reach the target MOI. Twenty-four hours after transduction (Day 1), media was changed, and cells were cultured for six days with fibroblast media changes every other day. Seven days after transduction (Day 7), transduced fibroblasts were replated onto 60-mm tissue culture dishes pre-coated with recombinant Vitronectin (Life Technologies) in fibroblast medium. After twenty-four hours, medium was replaced with Essential 8 Media (Life Technologies), and cells were cultured with iPSC media changes every day. Eight days after transduction (Day 8), the cells were checked under the microscope for the emergence of cell clumps indicative of transformed cells. Three to four weeks post-transduction after sufficient growth, individual undifferentiated colonies were selected by iPSC morphology, manually picked (passage 0) and transferred to plates pre-coated with Corning Matrigel Matrix (Thermo Fisher Scientific, Waltham, MA) in TeSR-E8 media (STEMCELL Technologies) for culture at 37C and 5% CO2 with daily media changes. The iPSC clones first were passaged manually (passage 1–5) and thereafter passaged using ReLeSR (STEMCELL Technologies). For each iPSC line, independent clones were created, serially passaged, expanded, and cryopreserved in Bambanker media (Thermo Fisher Scientific) for long-term storage in liquid nitrogen.

For each iPSC line at passage 10 or above, independent clones were validated for normal pluripotency (Additional file 1: Figure S8). iPSC clones were tested for positive staining by immunocytochemistry (ICC) of established pluripotency makers. For ICC, iPSCs clones for each line were grown, processed, and analyzed directly on Matrigel-coated, Nunc Lab-Tek 4-well Chamber Slides (Thermo Fisher Scientific) for pluripotent stem cell markers (OCT4, SOX2, SSEA4, and TRA-1-60) using the Pluripotent Stem Cell 4-Marker Immunocytochemistry Kit (A24881, Life Technologies). Cells were fixed, permeabilized, and incubated with blocking solution and antibodies (Additional file 1: Table S12). Cells were nuclear counterstained using Fluoroshield with DAPI (Sigma-Aldrich) and visualized using a Nikon Ti-E Inverted Fluorescent Microscope.

For each iPSC line at passage 9 or above, independent clones were validated for normal chromosome constitution by karyotype (Additional file 1: Figure S9). iPSC cultures in Matrigel-coated T25 flasks with TeSR-E8 media were sent to WiCell Genetics (Madison, WI) for routine study of G-banded chromosomes by counting 20 cells and analyzing eight cells. Karyotype results were classified as either normal (46,XX or 46,XY) or abnormal with clonal or nonclonal findings. Clonal findings were defined as chromosome gain or structural rearrangement in at least two cells or chromosome loss in at least three cells. Nonclonal findings were defined as chromosome gain and structural rearrangements in a single cell consistent with technical artifact, developing clonal abnormality, or low-level mosaicism. If the result of the first clone was abnormal (clonal or nonclonal), a second independent clone isolated from the iPSC line was analyzed by ICC and then karyotyped. This process was repeated until at least one chromosomally normal clone was identified with validation of pluripotency.

After fibroblast reprogramming and characterization for normal pluripotency and karyotype, iPSCs were cultured from cryopreserved vials and maintained on Matrigel-coated 6-well plates with mTESR1 for gDNA extraction. At 90–100% confluency, iPSCs were harvested using ReLeSR, and gDNA was isolated using MasterPure Complete DNA Purification Kit (Lucigen, Middleton, WI). Total gDNA was then quantified using Nanodrop Spectrophotometer (Thermo Fisher Scientific).

### RNA-sequencing (RNA-seq) and differentially expressed gene (DEG) analysis

Bulk RNA-seq was previously performed on Family A fibroblasts (3 unaffected mutation-negative family members and 3 patients heterozygous for *LMNA* splice-site (c.357-2A>G)) and 3 healthy, unrelated individuals (Donors 2, 3, and 4). A list of DEGs between patients, control siblings, and the unrelated controls was attained from GSE125990 [7]. DEGs were filtered for FDR-adjusted *p*-value ≤ 0.05. RNA-seq data for control and DCM heart tissue were accessed from GSE120836 [8]. Provided log_2_ fold change values of DCM over Control, filtered for genes with *p*-values ≤ 0.05, were then intersected with fibroblast DEGs for analyses.

### Reduced representation bisulfite sequencing (RRBS)

Extracted gDNA from fibroblasts and iPSCs was subjected to RRBS for DNA methylation analysis. For all twenty samples, 4.5 μg of DNA was first mixed with 4 μL MspI (20,000 U/mL, New England BioLabs) and 1 × CutSmart and incubated at 37˚C for 24 h. 0.5 × Agencourt Ampure XP beads (Beckman Coulter) were then used to keep fragments ≤ 300 bp, which were then concentrated using Zymo Clean and Concentrator kit’s protocol. Zymo DNA Methylation-Gold Kit was used according to manufacturer’s protocol to perform bisulfite conversion on all samples, with a final volume of 15 μL in elution buffer. The eluted DNA was then processed through the Accel-NGS Methyl-seq DNA library kit (Swift Biosciences), following the manufacturer’s protocol, for adapter ligation. Post-ligation DNA was subjected to 10 PCR cycles for indexing. PCR products were then eluted in 21 μL of low EDTA elution buffer, of which 1 μL was run in a 2200 TapeStation (Agilent) to ensure correct band sizes of approximately 300 bp. Pooled multiplex RRBS libraries were sent to the UCI Genomics High-Throughput Facility and sequenced on an Illumina HiSeq4000 sequencer. We performed paired-end sequencing runs for a total of 100 cycles.

### Differentially methylated region (DMR) analysis

Raw fastq files were trimmed by 11 bp on both 5’ and 3’ ends of both reads 1 and 2 using Trim Galore (Version 0.4.4) [54]. Trimmed reads were then aligned to hg19/GRCh37 using Bowtie2 [55] as part of Bismark (Version 0.20.1) [56]. Paired-end read mapping efficiency varied between 68.0 and 82.3%, with an average of 77.4% across all twenty samples (Additional file 1: Table S1). Bismark was used to make methylation calls, which were then merged for neighboring CpGs on opposite sides of the strand. Finally, the methylation ratios generated were filtered to keep only CpGs with a minimum read coverage of ≥ 5×, thus ensuring fair comparisons across samples.

DNA methylation data and DMRs were visualized across the hg19 genome using the Broad Institute’s Integrative Genome Viewer (IGV) [57], Circos and Trellis plots generated with R packages circlize (Version 0.4.5) [58] and gtrellis (Version 1.16.1) [59]. Hierarchical clustering of samples based on genome-wide DNA methylation was performed using the ward method as part of methylKit. Additional heatmaps of DNA methylation levels in DMRs was generated through heatmap.2 from R package gplots (Version 2.11.0) [60] was used to generate heatmap and corresponding dendrograms for DMRs.

To obtain DMCpGs, methylation call BAM files were inputted into the R package methylKit (Version 1.16.0) [61], with a specified minimum read coverage of 5 (≥ 5x) per sample and assembly hg19. The unite() function was then applied to compare methylation calls of ≥ 5 × CpGs, overlapped across all input samples, generated after destranding to merge methylation calls on both sides of DNA strand at CpG dinucleotides. A filter of minimum *q*-value of ≤ 0.01 and a ± 30% CpG methylation difference cutoff between CONTROL and PATIENT samples were used to ensure reliable differential methylation results. This generated a set of DMCpGs, where negative DNA methylation differences indicated scenarios where patient samples were hypomethylated relative to controls and positive differences indicated where patient samples were hypermethylated. DMRs were generated by merging neighboring DMCpGs within ± 500 bp of one another into a single tile. Tiles with a size < 100 bp were extended equally on each side until a size of 100 bp was attained, similar to previously described methods [62]. Tiles containing DMCpGs with methylation differences with opposite directionality (hyper- or hypomethylation) were considered ambiguous and were removed from further analyses (0.13–0.8% of total DMRs generated) (Additional file 1: Table S13). Methylation difference of DMCpGs falling within the same tile was averaged in the remaining DMRs. This methodology was applied with three different inputs (1) all samples, (2) Family A samples (C1, C3, P1, P2, P3), (3) Family C samples (C4, C5, P4, P5), thus yielding three categories of DMR tiles. To compare across all three categories, DMRs were filtered to keep only those with CpG methylation data overlapped in both Family A and Family C. DMR tiles from the three groups were reclassified as follows: “Family-Specific” tiles were defined as DMRs only found in one of two family DMR categories (2) or (3), described above, or found in one of the two family DMR categories (2) or (3) and in the all samples category (1). “Shared” tiles were defined as DMRs found in both categories (2) and (3), or found only in all samples (1) and not in family categories (2) or (3), or found in all three categories (1), (2), and (3). This DMR methodology and grouping was applied to both fibroblast and iPSC samples separately. When comparing iPSC DMRs to their fibroblast counterparts, tiles were filtered to keep only those that had CpG methylation in both cell types. A detailed workflow of the computational methods used for DNA methylation analyses in this study is available at Additional file 1: Figure S2.

### Genomic feature annotation of DMRs

To determine DMR association to inferred and experimentally derived genomic features, DMR files were annotated against ChromHMM’s 25-state chromatin model [63] for normal human dermal fibroblasts (NHDFs), acquired from NIH Epigenome Roadmap, and RefSeq genomic features and histone modifications for NHDFs and a human embryonic stem cell line (HUES64), acquired from UCSC genome table browser, using BEDTools’ “intersection” function [64]. Genomic promoter features were defined as 2 Kb upstream of gene transcription start sites (TSS) acquired from UCSC genome table browser. Intergenic features were acquired by finding regions outside of gene bodies, against acquired from UCSC genome table browser, using BEDTools’ “subtract” function. A list of double elite enhancer locations, including their associated genes, used for annotation was acquired from the GeneHancer database [65] available on the UCSC genome table browser.

### Identification of gene network and ontologies from DMR-associated gene lists

Stanford’s Genomic Regions Enrichment of Annotations Tool (GREAT) software [17] (Version 4.0.4) was used with default parameters (basal plus extension/proximal 5 Kb upstream, 1 Kb downstream, plus distal up to 1000 Kb) to find hg19 UCSC genes associated with input DMR files. From there, (1) disease ontology, (2) gene ontology, (3) protein–protein interaction networks, and (4) pathway enrichment analysis were performed as follows: (1) Disease ontology was performed on acquired gene lists using ToppFun, a part of the ToppGene suite [66], using default correction and *p*-value cutoff parameters (FDR correction with *p*-value ≤ 0.05) and “Gene Limits” increased to include the number of genes inputted. Additionally, gene lists related to diseases of interest were acquired from DisGeNET database [34] (Version 7.0). (2) Gene lists for GO terms heart development (GO:0007507) and skeletal system development (GO:0001501) were acquired from the AmiGO database [67, 68]. (3) Gene lists were submitted to STRING [69] (Version 11.0b) to identify protein–protein interaction (PPI) networks. The minimum required interaction score for all PPI was set at 0.700 (considered “high confidence”) for the network. (4) The Kyoto Encyclopedia of Genes and Genomes (KEGG) [70] database was used, as part of STRING [69], to identify enriched of pathways within a PPI network. Strength scores are calculated as log_10_(observed/expected) by STRING. Enriched pathways are filtered for a false discovery rate (calculated according to the Benjamini & Hochberg method [71]) ≤ 0.05 by STRING. PPI enrichment *p*-value for the generated network was provided by STRING.

### Determining differentially methylated transcription factor binding sites (TFBS)

DMR files, in BED format, were inputted into Hypergeometric Optimization of Motif EnRichment (HOMER) software [24] (Version 4.7) to identify enrichment of known TFBS motifs, reposited within the software’s vertebrae database. Analyses were performed with hg19 genome as background, along with a specified motif size parameter based on average DMR tile size. TFBS motif results were finally filtered for *p*-value ≤ 0.01. Known related categories for each transcription factor (TF) were determined using GeneCards’ Human Phenotype Ontology (HPO) and SuperPathways databases [72].

### Lamina-associated domain (LAD) redistribution analyses

*LMNA* peaks, generated by anti-lamin A/C ChIP-seq, from cardiomyocytes derived from DCM patients and control individuals were acquired from GSE120837 [8]. In order to determine the location of redistributed LAD, BEDtools’ “subtract” function [64] was used to compare DCM and control LAD locations. Gain of LAD (GoL) regions demarcated LAD locations that were present in diseased tissues but absent in unaffected donors. Loss of LAD (LoL) regions demarcated LAD locations that were present in unaffected donors but absent in diseased tissues. Regions where LADs were present in both control and diseased tissues were termed MoL (maintenance of LAD) regions.

LADs from normal human primary dermal fibroblast (AD04) were acquired from GSM1313399 [73] and compared to the aforementioned cardiomyocyte redistributed LADs to identify LADs conserved across both cell types. Fibroblast LADs locations were compared to those of the three LAD categories (GoL, LoL, and MoL) generated in the cardiomyocyte samples. Genomic regions identified as cardiomyocyte GoLs that did not overlap with a fibroblast LAD were kept for downstream analyses. Similarly, genomic regions annotated as LoLs and MoLs in cardiomyocytes that overlapped with a fibroblast LAD were retained for further analyses. Distance between DEGs and closest redistributed LADs was determined using BEDtools’ “closest” function [64].

### Statistical analyses

All statistical tests were performed through R (Version 2.15.2) [74]. Data distributions were first tested for normality using the Shapiro–Wilks test. The Kruskal–Wallis and Wilcoxon rank sum tests were performed for datasets with non-normal distribution.

Quadrant count ratio (QCR) was calculated as $\frac{{n\left( {{\text{Quadrant}}\,{\text{I}}} \right) + n\left( {{\text{Quadrant}}\,{\text{III}}} \right) - n\left( {{\text{Quadrant}}\,{\text{II}}} \right) - n\left( {{\text{Quadrant}}\,{\text{IV}}} \right)}}{{N_{{{\text{total}}}} }}$, where n(Quadrant) is the number of observations present within a given quadrant, and *N*_total_ is the total number of observations across all four quadrants.

Odds ratio (OR) analyses were performed to determine the significance of DMR association to particular chromatin contexts (for example, distance from a gene’s transcriptional start site (TSS), histone modifications, and ChromHMM annotations). CpGs (filtered for ≥ 5 × depth) captured in our RRBS study for each sample were merged according to the three categories previously described (all samples, Family A samples, Family C samples), thus creating three categories of background CpGs. The resulting background CpG files were then intersected with one of the six DMR files previously generated (Hyper and hypomethylated DMRs for shared, Family A, and Family C). Subsequently, the number of DMR-filtered CpGs and background CpGs that intersected with a particular context of interest were compared. For distance from a gene’s TSS, CpGs were intersected with bins of distance (from 0–1 Kb up to 10–50 Kb) in both up and downstream directions relative to each gene’s genomic orientation. For histone modifications and ChromHMM annotations, CpGs were simply intersected with the Chip-seq peak tiles or annotated tiles. OR was then calculated as follows: $\frac{{a/c~}}{{b/d}}$, where *a* = the number of CpGs that fall within a DMR and within the context of interest, *b* = the number of CpGs that fall within DMRs and outside of the context of interest, *c* = the number of CpGs that fall outside of DMRs and within the context of interest, *d* = the number of CpGs that fall outside of DMRs and outside of the context of interest. The logarithmic OR value (logOR) was then reported for each context of interest. Fisher’s exact test was used to determine significance of odds ratios.

To determine the significance of proximity between DMRs in different contexts of interest (across families or cell types), we randomly sampled our set of captured CpGs to match the number of differentially methylated CpGs found within each DMR category. We then calculated the distance between CpGs from one category to the nearest sampled CpG from the category of comparison (e.g., Family A CpGs vs. Family C CpGs, or iPSC CpGs vs. fibroblast CpGs). This comparison served as our background distribution for CpG distance in the context of interest. The same analysis was performed for differentially methylated CpGs. These distributions were plotted as a density distribution for interfamily CpG distance or using histogram bins for inter-cell type CpG distance. Significance was determine using Wilcoxon rank sum test and 1-tailed Fisher’s exact test for interfamily and inter-cell type analyses, respectively.

## Supplementary Information

**Additional file 1.** Contains Supplementary Tables 1–13 and Supplementary Figures 1–9.

## Data Availability

The fibroblast and iPSC RRBS datasets supporting the conclusions in this article are publicly available in the GEO repository (GSE164365) [75]. All supporting data are available in this published article and its supplementary information files.

## Abbreviations

RRBS: Reduced representation bisulfite sequencing
DMCpG: Differentially methylated CpG
DMR: Differentially methylated region
LAD: Lamina-associated domains
RNA-seq: RNA-sequencing
LoL: Loss of LAD
GoL: Gain of LAD
MoL: Maintenance of LAD
ChromHMM: Chromatin hidden Markov model
TFBS: Transcription factor binding site
TF: Transcription factor
DEG: Differentially expressed gene
DCM: Dilated cardiomyopathy
