# Supplementary material for: DNA methylation analysis reveals epimutation hotspots in patients with dilated cardiomyopathy-associated laminopathies
Source: Clin Epigenetics. 2021 Jul 10;13:139. doi: 10.1186/s13148-021-01127-0 (PMC8272901; doi:10.1186/s13148-021-01127-0)
Supplement: Supplementary file 1 — Additional file 1. Contains Supplementary Tables 1–13 and Supplementary Figures 1–9. [file 13148_2021_1127_MOESM1_ESM.docx]

**SUPPLEMENTARY MATERIAL**

**Contents**

1. **Supplementary Tables**...........................................................................................................3
   1. Table S1 - RRBS read and methylation call data before and after depth filtering for fibroblast (A) and iPSC (B)..............................................................................................3
   2. Table S2 - Number of genomic features captured in RRBS by each sample in fibroblast (A) and iPSC (B). ............................................................................................4
   3. Table S3 - Full list of genes associated to Shared DMRs included in GO term heart (A) and skeletal development (B)...................................................................................6
   4. Table S4 - OR statistics for fibroblast DMR and histone modifications. .......................6
   5. Table S5 - Odds ratio statistics for fibroblast DMRs and ChromHMM annotations.......8
   6. Table S6 - Complete list of TFBS motif enrichment for fibroblast DMRs acquired from HOMER.........................................................................................................................12
   7. Table S7 - Complete list of disease ontology terms from ToppGene for gene lists associated with either hypo or hypermethylated fibroblast DMR contexts...................16
   8. Table S8 - OR statistics for iPSC DMR and histone modifications..............................25
   9. Table S9 - Complete list of disease ontology terms from ToppGene for gene lists associated with fibroblast and iPSC DMRs...........................................................................................................................26
   10. Table S10 - KEGG pathway enrichment for the set of 28 genes associated to DMRs whose methylation change is hypermethylated in fibroblast and hypomethylated in iPSC, acquired from STRING.......................................................................................30
   11. Table S11 - Fibroblast and iPSC line pairs with corresponding genotype, sex, and age when skin biopsies were performed.............................................................................31
   12. Table S12 - Antibodies used for pluripotency characterization of iPSCs.....................31
   13. Table S13 - Hyper and hypomethylated DMR statistics in fibroblasts (A) and iPSCs (B) for all samples and by family........................................................................................32
2. **Supplementary Figures**.......................................................................................................34
   1. Figure S1 - Characterization of DNA methylation in *LMNA­*-mutant fibroblasts and iPSCs..........................................................................................................................34
   2. Figure S2 - Computational workflow of DNA methylation analyses............................35
   3. Figure S3 - Sex chromosomes had minimal impact on genome-wide and DMR results..........................................................................................................................37
   4. Figure S4 - Hypermethylated and hypomethylated DMRs localize at distal regulatory features and transcriptionally repressed chromatin in fibroblasts...............................39
   5. Figure S5 - Inter-DMR distances overlap across both families.
   6. Figure S6 - DMRs associate to dysregulated and disease-relevant genes near redistributed LADs.......................................................................................................40
   7. Figure S7 - DMRs in iPSCs reveal tissue-persistent epimutation hotspots at developmentally and laminopathy relevant genes......................................................40
   8. Figure S8 - Validation of induced pluripotent stem cell (iPSC) pluripotency...............42
   9. Figure S9 - Validation of normal chromosome constitution in each induced pluripotent stem cell clone.............................................................................................................43
3. **SUPPLEMENTARY TABLES**

**Table S1**

1. RRBS read and methylation call data before and after depth filtering for fibroblast lines

| **Cell ID** | **Family** | **Mapping efficiency (%)** | **# of mapped reads** | **Mean read depth** | **# of CpGs** | **# of CpGs with ≥5x depth** | **% of total CpGs with ≥5x depth** | **Mean methylation level (%) ≥5x** |
| --- | --- | --- | --- | --- | --- | --- | --- | --- |
| C2 | Donor | 80.10 | 11167514 | 5.93 | 5765443 | 2453192 | 42.55 | 62.27 |
| C1 | A | 78.90 | 12955944 | 7.62 | 5732669 | 2478080 | 43.23 | 59.15 |
| C3 | A | 76.40 | 9405102 | 5.07 | 5566829 | 2082358 | 37.41 | 59.21 |
| P1 | A | 79.70 | 11919395 | 4.96 | 6687065 | 2418012 | 36.16 | 64.54 |
| P2 | A | 74.70 | 11726435 | 5.55 | 6434663 | 2574977 | 40.02 | 60.37 |
| P3 | A | 68.00 | 8205263 | 4.06 | 5658271 | 1617429 | 28.59 | 61.75 |
| C4 | C | 71.60 | 9253758 | 4.79 | 5722213 | 1988035 | 34.74 | 61.66 |
| C5 | C | 74.70 | 8597498 | 4.77 | 5368763 | 1897892 | 35.35 | 60.61 |
| P4 | C | 71.30 | 9723382 | 5.27 | 5842361 | 2240017 | 38.34 | 58.93 |
| P5 | C | 79.20 | 9976610 | 4.96 | 6087224 | 2273148 | 37.34 | 61.52 |
| Control | Avg | 76.34 | 10275963.2 | 5.63 | 5631183.4 | 2179911.4 | 38.66 | 60.58 |
| Patient | Avg | 74.58 | 10310217 | 4.96 | 6141916.8 | 2224716.6 | 36.09 | 61.42 |
| All | Avg | 75.46 | 10293090.1 | 5.30 | 5886550.1 | 2202314 | 37.37 | 61.00 |

1. RRBS read and methylation call data before and after depth filtering for iPSC lines

| **Cell ID** | **Family** | **Mapping efficency (%)** | **# of mapped reads** | **Mean read depth** | **# of CpGs** | **# of CpGs with ≥5x depth** | **% of total CpGs with ≥5x depth** | **Mean methylation level (%) ≥5x** |
| --- | --- | --- | --- | --- | --- | --- | --- | --- |
| C2 | Donor | 81.8 | 15412513 | 3.76 | 9679992 | 2253977 | 23.28 | 68.94 |
| C1 | A | 82.3 | 14005546 | 3.37 | 9272820 | 1863154 | 20.09 | 69.89 |
| C3 | A | 79.9 | 12818488 | 5.22 | 7269552 | 2524314 | 34.72 | 68.85 |
| P1 | A | 80 | 13239664 | 5.01 | 7303946 | 2456742 | 33.64 | 70.97 |
| P2 | A | 74.4 | 13889429 | 4.16 | 8538282 | 2369711 | 27.75 | 71.06 |
| P3 | A | 77.3 | 11897293 | 4.01 | 7459790 | 1994609 | 26.74 | 70.85 |
| C4 | C | 74.7 | 12541395 | 4.16 | 8285819 | 2304838 | 27.82 | 70.27 |
| C5 | C | 80.9 | 11418775 | 4.74 | 6786321 | 2245024 | 33.08 | 70.43 |
| P4 | C | 81.1 | 12307525 | 3.33 | 8391565 | 1729506 | 20.61 | 69.99 |
| P5 | C | 81.2 | 11057593 | 4.44 | 6912886 | 2201672 | 31.85 | 71.62 |
| Control | Avg | 79.92 | 13239343.4 | 4.25 | 8258900.8 | 2238261.4 | 27.80 | 69.68 |
| Patient | Avg | 78.8 | 12478300.8 | 4.19 | 7721293.8 | 2150448 | 28.12 | 70.90 |
| All | Avg | 79.36 | 12858822.1 | 4.22 | 7990097.3 | 2194354.7 | 27.96 | 70.29 |

**Abbreviations:** RRBS, reduced representation bisulfite sequencing; iPSC, induced pluripotent stem cell; Avg, average

**Table S2**

1. Number of genomic features captured in RRBS by each sample in fibroblast

| **Feature** | **C1** | **C2** | **C3** | **C4** | **C5** | **P1** | **P2** | **P3** | **P4** | **P5** | ***All samples** | **total # of features** | **% of features in all samples** |
| --- | --- | --- | --- | --- | --- | --- | --- | --- | --- | --- | --- | --- | --- |
| promoters | 16100 | 16389 | 15799 | 15652 | 15446 | 16491 | 16769 | 14901 | 16230 | 16384 | 18686 | 28180 | 66.30 |
| exons | 43866 | 45265 | 40795 | 39704 | 38893 | 44346 | 47258 | 34130 | 43881 | 44157 | 62713 | 242221 | 25.89 |
| introns | 71387 | 74197 | 70271 | 69760 | 68389 | 75080 | 75997 | 64738 | 71261 | 73333 | 90438 | 188793 | 47.90 |
| intergenic | 17447 | 17576 | 17249 | 17124 | 17110 | 17574 | 17730 | 16817 | 17431 | 17522 | 18700 | 21508 | 86.94 |

1. Number of genomic features captured in RRBS by each sample in iPSC

| **Feature** | **C1** | **C2** | **C3** | **C4** | **C5** | **P1** | **P2** | **P3** | **P4** | **P5** | ***All samples** | **total # of features** | **% of features in all samples** |
| --- | --- | --- | --- | --- | --- | --- | --- | --- | --- | --- | --- | --- | --- |
| promoters | 15467 | 16401 | 16411 | 16524 | 16025 | 16494 | 16577 | 15869 | 15248 | 16091 | 19458 | 28180 | 69.05 |
| exons | 38711 | 44089 | 46231 | 45644 | 43324 | 45603 | 45212 | 40139 | 36913 | 43356 | 65888 | 242221 | 27.20 |
| introns | 70186 | 75164 | 74271 | 74233 | 72394 | 75095 | 75528 | 71255 | 68008 | 73273 | 98064 | 188793 | 51.94 |
| intergenic | 17210 | 17604 | 17554 | 17591 | 17414 | 17564 | 17626 | 17291 | 17026 | 17425 | 19061 | 21508 | 88.62 |

(*) Features found in all samples were merged together, without any duplicates

**Table S3**

1. Full list of genes associated to Shared DMRs included in GO term heart development (GO:0007507) (n=34)

| TBX3 | SOX11 | ZFPM2 | MYO18B |
| --- | --- | --- | --- |
| CACNA1C | RPS6KA2 | RBM20 | GLI2 |
| COL5A1 | FOXL1 | MSX1 | ZMIZ1 |
| ERBB4 | DLL1 | SMYD2 | FOXN4 |
| JMJD6 | DNAH5 | BMP7 |  |
| PKD1 | SMG9 | MIXL1 |  |
| FOLR1 | PDLIM3 | SORBS2 |  |
| FOXF1 | RXRA | GATA5 |  |
| EYA1 | ZFPM1 | SIX1 |  |
| ZFP36L1 | TAB1 | ZBTB14 |  |

1. Full list of genes associated to Shared DMRs included in GO term skeletal system development (GO: 0001501) (n=37)

| ALPL | FAM20C | MSX1 | SULF2 |
| --- | --- | --- | --- |
| ALX3 | FOXP1 | PBX1 | TBX3 |
| BMP7 | GLI2 | PKD1 | TPO |
| CHSY1 | GNAS | RASSF2 | TRPV4 |
| CYTL1 | HMGA2 | RPL13 | WDR5 |
| DLX1 | HOXD10 | RUNX3 | XYLT1 |
| DLX2 | HOXD12 | SIX1 | ZFPM1 |
| DSCAML1 | LHX1 | SNX19 |  |
| EYA1 | LRRK1 | SOX11 |  |
| FAM101A | MMP2 | SP5 |  |

**Table S4**

**OR statistics for fibroblast DMR and histone modifications**

| **DMR Group** | **DMR Type** | **Histone Mark** | **a*** | **b*** | **c*** | **d*** | **OR†** | **log(OR)** | **p-value‡** |
| --- | --- | --- | --- | --- | --- | --- | --- | --- | --- |
| Shared | Hyper | H3K27Ac | 326 | 1015793 | 1126 | 3408263 | 0.97 | -0.01 | 6.62E-01 |
| Shared | Hyper | H3K27me3 | 532 | 1238261 | 920 | 3185589 | 1.49 | 0.17 | 1.02E-12 |
| Shared | Hyper | H3K36me3 | 366 | 1355818 | 1086 | 3068198 | 0.76 | -0.12 | 5.12E-06 |
| Shared | Hyper | H3K4me1 | 445 | 899637 | 1007 | 3524300 | 1.73 | 0.24 | 2.08E-20 |
| Shared | Hyper | H3K4me3 | 328 | 951104 | 1124 | 3472950 | 1.07 | 0.03 | 3.07E-01 |
| Shared | Hyper | H3K9me3 | 308 | 747719 | 1144 | 3676355 | 1.32 | 0.12 | 2.19E-05 |
| Shared | Hypo | H3K27Ac | 116 | 1016003 | 890 | 3408709 | 0.44 | -0.36 | 1.95E-20 |
| Shared | Hypo | H3K27me3 | 361 | 1238432 | 645 | 3186035 | 1.44 | 0.16 | 5.85E-08 |
| Shared | Hypo | H3K36me3 | 235 | 1355949 | 771 | 3068644 | 0.69 | -0.16 | 3.27E-07 |
| Shared | Hypo | H3K4me1 | 169 | 899913 | 837 | 3524746 | 0.79 | -0.10 | 4.78E-03 |
| Shared | Hypo | H3K4me3 | 160 | 951272 | 846 | 3473396 | 0.69 | -0.16 | 9.73E-06 |
| Shared | Hypo | H3K9me3 | 249 | 747778 | 757 | 3676801 | 1.62 | 0.21 | 2.96E-10 |
| Family A | Hyper | H3K27Ac | 1946 | 912786 | 9406 | 3009559 | 0.68 | -0.17 | 2.13E-57 |
| Family A | Hyper | H3K27me3 | 3613 | 1099621 | 7739 | 2821057 | 1.20 | 0.08 | 1.01E-18 |
| Family A | Hyper | H3K36me3 | 2645 | 1211411 | 8707 | 2710235 | 0.68 | -0.17 | 6.64E-72 |
| Family A | Hyper | H3K4me1 | 2502 | 801355 | 8850 | 3120434 | 1.10 | 0.04 | 2.71E-05 |
| Family A | Hyper | H3K4me3 | 1931 | 860760 | 9421 | 3061600 | 0.73 | -0.14 | 5.58E-39 |
| Family A | Hyper | H3K9me3 | 2626 | 661875 | 8726 | 3259790 | 1.48 | 0.17 | 6.65E-65 |
| Family A | Hypo | H3K27Ac | 1540 | 913192 | 5963 | 3013408 | 0.85 | -0.07 | 1.47E-08 |
| Family A | Hypo | H3K27me3 | 2781 | 1100453 | 4722 | 2824906 | 1.51 | 0.18 | 3.08E-64 |
| Family A | Hypo | H3K36me3 | 1871 | 1212185 | 5632 | 2714084 | 0.74 | -0.13 | 8.57E-30 |
| Family A | Hypo | H3K4me1 | 1896 | 801961 | 5607 | 3124283 | 1.32 | 0.12 | 4.61E-24 |
| Family A | Hypo | H3K4me3 | 1654 | 861037 | 5849 | 3065449 | 1.01 | 0.00 | 8.12E-01 |
| Family A | Hypo | H3K9me3 | 1475 | 663026 | 6028 | 3263639 | 1.20 | 0.08 | 3.37E-10 |
| Family C | Hyper | H3K27Ac | 1405 | 805630 | 7602 | 2623702 | 0.60 | -0.22 | 4.48E-76 |
| Family C | Hyper | H3K27me3 | 3151 | 963853 | 5856 | 2463733 | 1.38 | 0.14 | 1.75E-45 |
| Family C | Hyper | H3K36me3 | 2013 | 1058107 | 6994 | 2370617 | 0.64 | -0.19 | 2.21E-72 |
| Family C | Hyper | H3K4me1 | 1701 | 707970 | 7306 | 2721066 | 0.89 | -0.05 | 3.20E-05 |
| Family C | Hyper | H3K4me3 | 1362 | 763549 | 7645 | 2665826 | 0.62 | -0.21 | 7.55E-65 |
| Family C | Hyper | H3K9me3 | 1696 | 574902 | 7311 | 2854139 | 1.15 | 0.06 | 2.53E-07 |
| Family C | Hypo | H3K27Ac | 1231 | 805804 | 5425 | 2626053 | 0.74 | -0.13 | 8.45E-23 |
| Family C | Hypo | H3K27me3 | 2421 | 964583 | 4235 | 2466084 | 1.46 | 0.16 | 3.34E-48 |
| Family C | Hypo | H3K36me3 | 1586 | 1058534 | 5070 | 2372968 | 0.70 | -0.15 | 8.19E-37 |
| Family C | Hypo | H3K4me1 | 1391 | 708280 | 5265 | 2723417 | 1.02 | 0.01 | 6.06E-01 |
| Family C | Hypo | H3K4me3 | 1352 | 763559 | 5304 | 2668177 | 0.89 | -0.05 | 1.33E-04 |
| Family C | Hypo | H3K9me3 | 1249 | 575349 | 5407 | 2856490 | 1.15 | 0.06 | 1.68E-05 |

(*) *a*, *b*, *c*, and *d* values are the contingency parameters used to calculate OR. (†) OR was calculated as described in the Methods section. (‡) P-values were calculated by Fisher’s exact test

**Abbreviations:** DMR, differentially methylated region; OR, odds ratio

**Table S5**

**Odds ratio statistics for fibroblast DMRs and ChromHMM annotations**

| **DMR Group** | **DMR Type** | **Annotation** | **a*** | **b*** | **c*** | **d*** | **OR†** | **log(OR)** | **p-value‡** |
| --- | --- | --- | --- | --- | --- | --- | --- | --- | --- |
| Shared | Hyper | 1_TssA | 11 | 187497 | 1441 | 4236874 | 0.17 | -0.76 | 2.75E-15 |
| Shared | Hyper | 2_PromU | 41 | 176466 | 1411 | 4247875 | 0.70 | -0.16 | 2.23E-02 |
| Shared | Hyper | 3_PromD1 | 33 | 186423 | 1419 | 4237926 | 0.53 | -0.28 | 8.33E-05 |
| Shared | Hyper | 4_PromD2 | 33 | 34490 | 1419 | 4389859 | 2.96 | 0.47 | 1.07E-07 |
| Shared | Hyper | 5_Tx5' | 51 | 126216 | 1401 | 4298115 | 1.24 | 0.09 | 1.34E-01 |
| Shared | Hyper | 6_Tx | 8 | 58069 | 1444 | 4366305 | 0.42 | -0.38 | 7.50E-03 |
| Shared | Hyper | 7_Tx3' | 39 | 313455 | 1413 | 4110888 | 0.36 | -0.44 | 2.24E-13 |
| Shared | Hyper | 8_TxWk | 76 | 357266 | 1376 | 4067040 | 0.63 | -0.20 | 3.26E-05 |
| Shared | Hyper | 9_TxReg | 28 | 32655 | 1424 | 4391699 | 2.64 | 0.42 | 7.20E-06 |
| Shared | Hyper | 10_TxEnh5' | 7 | 20788 | 1445 | 4403587 | 1.03 | 0.01 | 8.47E-01 |
| Shared | Hyper | 11_TxEnh3' | 9 | 17011 | 1443 | 4407362 | 1.62 | 0.21 | 1.37E-01 |
| Shared | Hyper | 12_TxEnhW | 29 | 22785 | 1423 | 4401568 | 3.94 | 0.60 | 1.60E-09 |
| Shared | Hyper | 13_EnhA1 | 4 | 22488 | 1448 | 4401890 | 0.54 | -0.27 | 2.67E-01 |
| Shared | Hyper | 14_EnhA2 | 20 | 17341 | 1432 | 4407021 | 3.55 | 0.55 | 2.26E-06 |
| Shared | Hyper | 15_EnhAF | 23 | 37791 | 1429 | 4386568 | 1.87 | 0.27 | 5.94E-03 |
| Shared | Hyper | 16_EnhW1 | 26 | 39436 | 1426 | 4384920 | 2.03 | 0.31 | 1.08E-03 |
| Shared | Hyper | 17_EnhW2 | 30 | 56505 | 1422 | 4367847 | 1.63 | 0.21 | 1.32E-02 |
| Shared | Hyper | 18_EnhAc | 15 | 12699 | 1437 | 4411668 | 3.63 | 0.56 | 3.06E-05 |
| Shared | Hyper | 19_DNase | 33 | 31149 | 1419 | 4393200 | 3.28 | 0.52 | 1.04E-08 |
| Shared | Hyper | 20_ZNF/Rpts | 4 | 4576 | 1448 | 4419802 | 2.67 | 0.43 | 6.58E-02 |
| Shared | Hyper | 21_Het | 27 | 24691 | 1425 | 4399664 | 3.38 | 0.53 | 1.19E-07 |
| Shared | Hyper | 22_PromP | 24 | 61721 | 1428 | 4362637 | 1.19 | 0.07 | 3.70E-01 |
| Shared | Hyper | 23_PromBiv | 52 | 130099 | 1400 | 4294231 | 1.23 | 0.09 | 1.61E-01 |
| Shared | Hyper | 24_ReprPC | 197 | 367844 | 1255 | 4056341 | 1.73 | 0.24 | 2.06E-11 |
| Shared | Hyper | 25_Quies | 635 | 2080494 | 817 | 2343253 | 0.88 | -0.06 | 1.25E-02 |
| Shared | Hypo | 1_TssA | 14 | 187494 | 992 | 4237320 | 0.32 | -0.50 | 4.20E-07 |
| Shared | Hypo | 2_PromU | 10 | 176497 | 996 | 4248321 | 0.24 | -0.62 | 1.75E-08 |
| Shared | Hypo | 3_PromD1 | 6 | 186450 | 1000 | 4238372 | 0.14 | -0.87 | 3.28E-12 |
| Shared | Hypo | 4_PromD2 | 14 | 34509 | 992 | 4390305 | 1.80 | 0.25 | 4.45E-02 |
| Shared | Hypo | 5_Tx5' | 14 | 126253 | 992 | 4298561 | 0.48 | -0.32 | 3.19E-03 |
| Shared | Hypo | 6_Tx | 14 | 58063 | 992 | 4366751 | 1.06 | 0.03 | 7.81E-01 |
| Shared | Hypo | 7_Tx3' | 29 | 313465 | 977 | 4111334 | 0.39 | -0.41 | 8.33E-09 |
| Shared | Hypo | 8_TxWk | 40 | 357302 | 966 | 4067486 | 0.47 | -0.33 | 2.24E-07 |
| Shared | Hypo | 9_TxReg | 12 | 32671 | 994 | 4392145 | 1.62 | 0.21 | 9.54E-02 |
| Shared | Hypo | 10_TxEnh5' | 11 | 20784 | 995 | 4404033 | 2.34 | 0.37 | 9.20E-03 |
| Shared | Hypo | 11_TxEnh3' | 2 | 17018 | 1004 | 4407808 | 0.52 | -0.29 | 6.03E-01 |
| Shared | Hypo | 12_TxEnhW | 1 | 22813 | 1005 | 4402014 | 0.19 | -0.72 | 7.29E-02 |
| Shared | Hypo | 13_EnhA1 | 0 | 22492 | 1006 | 4402336 | 0.00 | -Inf | 1.22E-02 |
| Shared | Hypo | 14_EnhA2 | 6 | 17355 | 1000 | 4407467 | 1.52 | 0.18 | 3.01E-01 |
| Shared | Hypo | 15_EnhAF | 3 | 37811 | 1003 | 4387014 | 0.35 | -0.46 | 5.69E-02 |
| Shared | Hypo | 16_EnhW1 | 7 | 39455 | 999 | 4385366 | 0.78 | -0.11 | 6.16E-01 |
| Shared | Hypo | 17_EnhW2 | 3 | 56532 | 1003 | 4368293 | 0.23 | -0.64 | 2.72E-03 |
| Shared | Hypo | 18_EnhAc | 0 | 12714 | 1006 | 4412114 | 0.00 | -Inf | 1.28E-01 |
| Shared | Hypo | 19_DNase | 19 | 31163 | 987 | 4393646 | 2.71 | 0.43 | 1.41E-04 |
| Shared | Hypo | 20_ZNF/Rpts | 1 | 4579 | 1005 | 4420248 | 0.96 | -0.02 | 1.00E+00 |
| Shared | Hypo | 21_Het | 11 | 24707 | 995 | 4400110 | 1.97 | 0.29 | 3.20E-02 |
| Shared | Hypo | 22_PromP | 36 | 61709 | 970 | 4363083 | 2.62 | 0.42 | 5.38E-07 |
| Shared | Hypo | 23_PromBiv | 26 | 130125 | 980 | 4294677 | 0.88 | -0.06 | 5.75E-01 |
| Shared | Hypo | 24_ReprPC | 253 | 367788 | 753 | 4056787 | 3.71 | 0.57 | 2.11E-57 |
| Shared | Hypo | 25_Quies | 475 | 2080654 | 531 | 2343699 | 1.01 | 0.00 | 9.25E-01 |
| Family A | Hyper | 1_TssA | 58 | 171117 | 11294 | 3753116 | 0.11 | -0.95 | 6.38E-141 |
| Family A | Hyper | 2_PromU | 336 | 160571 | 11016 | 3763384 | 0.71 | -0.15 | 2.21E-10 |
| Family A | Hyper | 3_PromD1 | 93 | 170717 | 11259 | 3753481 | 0.18 | -0.74 | 2.19E-111 |
| Family A | Hyper | 4_PromD2 | 204 | 30961 | 11148 | 3893126 | 2.30 | 0.36 | 2.57E-25 |
| Family A | Hyper | 5_Tx5' | 196 | 112115 | 11156 | 3811980 | 0.60 | -0.22 | 1.08E-14 |
| Family A | Hyper | 6_Tx | 126 | 51250 | 11226 | 3872915 | 0.85 | -0.07 | 6.83E-02 |
| Family A | Hyper | 7_Tx3' | 446 | 278242 | 10906 | 3645603 | 0.54 | -0.27 | 8.39E-46 |
| Family A | Hyper | 8_TxWk | 511 | 317826 | 10841 | 3605954 | 0.53 | -0.27 | 3.50E-52 |
| Family A | Hyper | 9_TxReg | 203 | 28851 | 11149 | 3895237 | 2.46 | 0.39 | 1.26E-28 |
| Family A | Hyper | 10_TxEnh5' | 46 | 18226 | 11306 | 3906019 | 0.87 | -0.06 | 4.06E-01 |
| Family A | Hyper | 11_TxEnh3' | 56 | 14927 | 11296 | 3909308 | 1.30 | 0.11 | 5.60E-02 |
| Family A | Hyper | 12_TxEnhW | 82 | 20017 | 11270 | 3904192 | 1.42 | 0.15 | 2.91E-03 |
| Family A | Hyper | 13_EnhA1 | 73 | 19476 | 11279 | 3904742 | 1.30 | 0.11 | 3.20E-02 |
| Family A | Hyper | 14_EnhA2 | 75 | 15074 | 11277 | 3909142 | 1.72 | 0.24 | 1.33E-05 |
| Family A | Hyper | 15_EnhAF | 132 | 32884 | 11220 | 3891275 | 1.39 | 0.14 | 3.01E-04 |
| Family A | Hyper | 16_EnhW1 | 167 | 35231 | 11185 | 3888893 | 1.65 | 0.22 | 2.63E-09 |
| Family A | Hyper | 17_EnhW2 | 189 | 49086 | 11163 | 3875016 | 1.34 | 0.13 | 1.64E-04 |
| Family A | Hyper | 18_EnhAc | 28 | 11248 | 11324 | 3913015 | 0.86 | -0.07 | 4.82E-01 |
| Family A | Hyper | 19_DNase | 125 | 27672 | 11227 | 3896494 | 1.57 | 0.20 | 2.89E-06 |
| Family A | Hyper | 20_ZNF/Rpts | 13 | 4079 | 11339 | 3920199 | 1.10 | 0.04 | 6.61E-01 |
| Family A | Hyper | 21_Het | 183 | 22073 | 11169 | 3902035 | 2.90 | 0.46 | 3.88E-34 |
| Family A | Hyper | 22_PromP | 201 | 54673 | 11151 | 3869417 | 1.28 | 0.11 | 9.97E-04 |
| Family A | Hyper | 23_PromBiv | 380 | 119007 | 10972 | 3804904 | 1.11 | 0.04 | 5.50E-02 |
| Family A | Hyper | 24_ReprPC | 1460 | 325138 | 9892 | 3597693 | 1.63 | 0.21 | 1.03E-60 |
| Family A | Hyper | 25_Quies | 5955 | 1831369 | 5397 | 2086967 | 1.26 | 0.10 | 4.32E-34 |
| Family A | Hypo | 1_TssA | 59 | 171116 | 7444 | 3756965 | 0.17 | -0.76 | 3.71E-76 |
| Family A | Hypo | 2_PromU | 345 | 160562 | 7158 | 3767233 | 1.13 | 0.05 | 2.86E-02 |
| Family A | Hypo | 3_PromD1 | 178 | 170632 | 7325 | 3757330 | 0.54 | -0.27 | 1.05E-19 |
| Family A | Hypo | 4_PromD2 | 165 | 31000 | 7338 | 3896975 | 2.83 | 0.45 | 8.79E-30 |
| Family A | Hypo | 5_Tx5' | 105 | 112206 | 7398 | 3815829 | 0.48 | -0.32 | 7.70E-17 |
| Family A | Hypo | 6_Tx | 30 | 51346 | 7473 | 3876764 | 0.30 | -0.52 | 1.06E-15 |
| Family A | Hypo | 7_Tx3' | 324 | 278364 | 7179 | 3649452 | 0.59 | -0.23 | 1.72E-23 |
| Family A | Hypo | 8_TxWk | 382 | 317955 | 7121 | 3609803 | 0.61 | -0.22 | 3.72E-24 |
| Family A | Hypo | 9_TxReg | 109 | 28945 | 7394 | 3899086 | 1.99 | 0.30 | 1.33E-10 |
| Family A | Hypo | 10_TxEnh5' | 44 | 18228 | 7459 | 3909868 | 1.27 | 0.10 | 1.25E-01 |
| Family A | Hypo | 11_TxEnh3' | 27 | 14956 | 7476 | 3913157 | 0.94 | -0.02 | 8.51E-01 |
| Family A | Hypo | 12_TxEnhW | 42 | 20057 | 7461 | 3908041 | 1.10 | 0.04 | 5.17E-01 |
| Family A | Hypo | 13_EnhA1 | 48 | 19501 | 7455 | 3908591 | 1.29 | 0.11 | 8.36E-02 |
| Family A | Hypo | 14_EnhA2 | 55 | 15094 | 7448 | 3912991 | 1.91 | 0.28 | 1.37E-05 |
| Family A | Hypo | 15_EnhAF | 106 | 32910 | 7397 | 3895124 | 1.70 | 0.23 | 6.46E-07 |
| Family A | Hypo | 16_EnhW1 | 133 | 35265 | 7370 | 3892742 | 1.99 | 0.30 | 1.08E-12 |
| Family A | Hypo | 17_EnhW2 | 150 | 49125 | 7353 | 3878865 | 1.61 | 0.21 | 7.42E-08 |
| Family A | Hypo | 18_EnhAc | 31 | 11245 | 7472 | 3916864 | 1.45 | 0.16 | 5.03E-02 |
| Family A | Hypo | 19_DNase | 86 | 27711 | 7417 | 3900343 | 1.63 | 0.21 | 2.36E-05 |
| Family A | Hypo | 20_ZNF/Rpts | 8 | 4084 | 7495 | 3924048 | 1.03 | 0.01 | 8.57E-01 |
| Family A | Hypo | 21_Het | 65 | 22191 | 7438 | 3905884 | 1.54 | 0.19 | 1.14E-03 |
| Family A | Hypo | 22_PromP | 154 | 54720 | 7349 | 3873266 | 1.48 | 0.17 | 5.36E-06 |
| Family A | Hypo | 23_PromBiv | 376 | 119011 | 7127 | 3808753 | 1.69 | 0.23 | 4.23E-20 |
| Family A | Hypo | 24_ReprPC | 1147 | 325451 | 6356 | 3601542 | 2.00 | 0.30 | 9.69E-88 |
| Family A | Hypo | 25_Quies | 3335 | 1833989 | 4168 | 2090816 | 0.91 | -0.04 | 7.83E-05 |
| Family C | Hyper | 1_TssA | 28 | 150782 | 8979 | 3279927 | 0.07 | -1.17 | 2.61E-132 |
| Family C | Hyper | 2_PromU | 177 | 144059 | 8830 | 3286501 | 0.46 | -0.34 | 1.23E-31 |
| Family C | Hyper | 3_PromD1 | 69 | 153340 | 8938 | 3277328 | 0.16 | -0.78 | 2.94E-96 |
| Family C | Hyper | 4_PromD2 | 141 | 27367 | 8866 | 3403229 | 1.98 | 0.30 | 4.09E-13 |
| Family C | Hyper | 5_Tx5' | 126 | 97654 | 8881 | 3332957 | 0.48 | -0.31 | 9.69E-20 |
| Family C | Hyper | 6_Tx | 79 | 45364 | 8928 | 3385294 | 0.66 | -0.18 | 1.21E-04 |
| Family C | Hyper | 7_Tx3' | 476 | 244075 | 8531 | 3186186 | 0.73 | -0.14 | 2.08E-12 |
| Family C | Hyper | 8_TxWk | 550 | 273945 | 8457 | 3156242 | 0.75 | -0.13 | 1.03E-11 |
| Family C | Hyper | 9_TxReg | 69 | 26335 | 8938 | 3404333 | 1.00 | 0.00 | 1.00E+00 |
| Family C | Hyper | 10_TxEnh5' | 32 | 15901 | 8975 | 3414804 | 0.77 | -0.12 | 1.40E-01 |
| Family C | Hyper | 11_TxEnh3' | 44 | 13370 | 8963 | 3417323 | 1.25 | 0.10 | 1.49E-01 |
| Family C | Hyper | 12_TxEnhW | 41 | 17594 | 8966 | 3413102 | 0.89 | -0.05 | 5.06E-01 |
| Family C | Hyper | 13_EnhA1 | 38 | 17315 | 8969 | 3413384 | 0.84 | -0.08 | 2.97E-01 |
| Family C | Hyper | 14_EnhA2 | 52 | 13301 | 8955 | 3417384 | 1.49 | 0.17 | 6.37E-03 |
| Family C | Hyper | 15_EnhAF | 99 | 29015 | 8908 | 3401623 | 1.30 | 0.11 | 1.12E-02 |
| Family C | Hyper | 16_EnhW1 | 110 | 31534 | 8897 | 3399093 | 1.33 | 0.12 | 3.99E-03 |
| Family C | Hyper | 17_EnhW2 | 115 | 43366 | 8892 | 3387256 | 1.01 | 0.00 | 8.87E-01 |
| Family C | Hyper | 18_EnhAc | 23 | 9793 | 8984 | 3420921 | 0.89 | -0.05 | 6.92E-01 |
| Family C | Hyper | 19_DNase | 82 | 24391 | 8925 | 3406264 | 1.28 | 0.11 | 2.79E-02 |
| Family C | Hyper | 20_ZNF/Rpts | 12 | 3572 | 8995 | 3427153 | 1.28 | 0.11 | 4.09E-01 |
| Family C | Hyper | 21_Het | 114 | 19776 | 8893 | 3410847 | 2.21 | 0.34 | 9.60E-14 |
| Family C | Hyper | 22_PromP | 89 | 47610 | 8918 | 3383038 | 0.71 | -0.15 | 8.27E-04 |
| Family C | Hyper | 23_PromBiv | 361 | 107137 | 8646 | 3323239 | 1.30 | 0.11 | 3.42E-06 |
| Family C | Hyper | 24_ReprPC | 1172 | 290148 | 7835 | 3139417 | 1.62 | 0.21 | 1.89E-47 |
| Family C | Hyper | 25_Quies | 4896 | 1580512 | 4111 | 1845329 | 1.39 | 0.14 | 7.21E-55 |
| Family C | Hypo | 1_TssA | 65 | 150745 | 6591 | 3282278 | 0.21 | -0.67 | 1.31E-59 |
| Family C | Hypo | 2_PromU | 232 | 144004 | 6424 | 3288852 | 0.82 | -0.08 | 3.62E-03 |
| Family C | Hypo | 3_PromD1 | 123 | 153286 | 6533 | 3279679 | 0.40 | -0.39 | 3.41E-31 |
| Family C | Hypo | 4_PromD2 | 88 | 27420 | 6568 | 3405580 | 1.66 | 0.22 | 9.46E-06 |
| Family C | Hypo | 5_Tx5' | 94 | 97686 | 6562 | 3335308 | 0.49 | -0.31 | 1.20E-14 |
| Family C | Hypo | 6_Tx | 50 | 45393 | 6606 | 3387645 | 0.56 | -0.25 | 1.27E-05 |
| Family C | Hypo | 7_Tx3' | 311 | 244240 | 6345 | 3188537 | 0.64 | -0.19 | 2.73E-16 |
| Family C | Hypo | 8_TxWk | 311 | 274184 | 6345 | 3158593 | 0.56 | -0.25 | 9.77E-27 |
| Family C | Hypo | 9_TxReg | 78 | 26326 | 6578 | 3406684 | 1.53 | 0.19 | 4.13E-04 |
| Family C | Hypo | 10_TxEnh5' | 57 | 15876 | 6599 | 3417155 | 1.86 | 0.27 | 1.83E-05 |
| Family C | Hypo | 11_TxEnh3' | 22 | 13392 | 6634 | 3419674 | 0.85 | -0.07 | 4.91E-01 |
| Family C | Hypo | 12_TxEnhW | 28 | 17607 | 6628 | 3415453 | 0.82 | -0.09 | 3.44E-01 |
| Family C | Hypo | 13_EnhA1 | 78 | 17275 | 6578 | 3415735 | 2.34 | 0.37 | 5.14E-11 |
| Family C | Hypo | 14_EnhA2 | 40 | 13313 | 6616 | 3419735 | 1.55 | 0.19 | 9.80E-03 |
| Family C | Hypo | 15_EnhAF | 47 | 29067 | 6609 | 3403974 | 0.83 | -0.08 | 2.28E-01 |
| Family C | Hypo | 16_EnhW1 | 96 | 31548 | 6560 | 3401444 | 1.58 | 0.20 | 3.55E-05 |
| Family C | Hypo | 17_EnhW2 | 83 | 43398 | 6573 | 3389607 | 0.99 | -0.01 | 9.56E-01 |
| Family C | Hypo | 18_EnhAc | 21 | 9795 | 6635 | 3423272 | 1.11 | 0.04 | 6.44E-01 |
| Family C | Hypo | 19_DNase | 78 | 24395 | 6578 | 3408615 | 1.66 | 0.22 | 3.87E-05 |
| Family C | Hypo | 20_ZNF/Rpts | 3 | 3581 | 6653 | 3429504 | 0.43 | -0.36 | 1.79E-01 |
| Family C | Hypo | 21_Het | 92 | 19798 | 6564 | 3413198 | 2.42 | 0.38 | 1.53E-13 |
| Family C | Hypo | 22_PromP | 177 | 47522 | 6479 | 3385389 | 1.95 | 0.29 | 2.64E-15 |
| Family C | Hypo | 23_PromBiv | 231 | 107267 | 6425 | 3325590 | 1.11 | 0.05 | 1.05E-01 |
| Family C | Hypo | 24_ReprPC | 1062 | 290258 | 5594 | 3141768 | 2.05 | 0.31 | 4.78E-87 |
| Family C | Hypo | 25_Quies | 3189 | 1582219 | 3467 | 1847680 | 1.07 | 0.03 | 3.68E-03 |

(*) *a*, *b*, *c*, and *d* values are the contingency parameters used to calculate OR. (†) OR was calculated as described in the Methods section. (‡) P-values were calculated by Fisher’s exact test.

**Abbreviations:** DMR, differentially methylated region; OR, odds ratio; ChromHMM, Chromatin hidden markov model.

**Table S6**

**Complete list of TFBS motif enrichment for fibroblast DMRs acquired from HOMER**

| **DMR group** | **DMR type** | **Rank** | **Motif** | **Related Gene Name** | **HOMER TF Name** | **-Log(p-value)*** |
| --- | --- | --- | --- | --- | --- | --- |
| Family A | Hypo | 1 | TCAGACGTAGTCTCGAAGTCTCAGGCATCTAGCTAGAGCT | USF2 | Usf2(bHLH)/C2C12-Usf2-ChIP-Seq(GSE36030)/Homer | 19.61 |
| Family A | Hypo | 2 | TCGATCAGTCGAACTGCATGACGTAGTCCTGA | NR2F2 | COUP-TFII(NR)/Artia-Nr2f2-ChIP-Seq(GSE46497)/Homer | 12.41 |
| Family A | Hypo | 3 | AGTCTGCATCGACTGAACTGCATGACGTATGCGTCATACG | ESRRA | Erra(NR)/HepG2-Erra-ChIP-Seq(GSE31477)/Homer | 9.41 |
| Family A | Hypo | 4 | TACGTCAGGATCGTACTCGAGACTGCTAGCTAGCTACGTAGATCGTCA | CDX4 | CDX4(Homeobox)/ZebrafishEmbryos-Cdx4.Myc-ChIP-Seq(GSE48254)/Homer | 9.19 |
| Family A | Hypo | 5 | ATGCGACTACTGCAGTGATCACGTTACGTACG | SMAD2 | Smad2(MAD)/ES-SMAD2-ChIP-Seq(GSE29422)/Homer | 8.00 |
| Family A | Hypo | 6 | AGTCGACTCAGTGTACAGTCATCGTCAGACTGGTCACGTA | STAT3 | Stat3(Stat)/mES-Stat3-ChIP-Seq(GSE11431)/Homer | 6.48 |
| Family A | Hypo | 7 | AGCTGACTCTAGCGTACATGCGATCTAGATCGGACTCAGT | NKX3-2 | Bapx1(Homeobox)/VertebralCol-Bapx1-ChIP-Seq(GSE36672)/Homer | 6.37 |
| Family A | Hypo | 8 | CGATTAGCGACTGTCACGTAACGTCGTACGTACGTAGCTA | HOXD13 | HOXD13(Homeobox)/Chicken-Hoxd13-ChIP-Seq(GSE38910)/Homer | 6.22 |
| Family A | Hypo | 9 | TACGATCGTAGCGATCACTGACGTAGTCACGTCTAGATCG | SMAD4 | Smad4(MAD)/ESC-SMAD4-ChIP-Seq(GSE29422)/Homer | 5.91 |
| Family A | Hypo | 10 | CATGGTACGACTGCTACGTACGTACGTAGCTAGACTCTGATCAGGTAC | MEF2C | Mef2c(MADS)/GM12878-Mef2c-ChIP-Seq(GSE32465)/Homer | 5.86 |
| Family A | Hypo | 11 | GTACGACTCGTACTGATCGACGTAGCTACAGTCTGATACG | MEF2A | Mef2a(MADS)/HL1-Mef2a.biotin-ChIP-Seq(GSE21529)/Homer | 5.62 |
| Family A | Hypo | 12 | CATGAGTCGACTCGTACGATGCATGACTGCATCGATCTAGCATGTGAC | MEF2B | Mef2b(MADS)/HEK293-Mef2b.V5-ChIP-Seq(GSE67450)/Homer | 5.49 |
| Family A | Hypo | 13 | CGATACGTACGTACGTCGTAAGCTCAGTCTAGATCGACTG | HOXB13 | HOXB13(Homeobox)/ProstateTumor-HOXB13-ChIP-Seq(GSE56288)/Homer | 5.37 |
| Family A | Hypo | 14 | CTGATCGACGTAATGCCGTACGTACGATCTAGTCAGGATC | SOX15 | Sox15(HMG)/CPA-Sox15-ChIP-Seq(GSE62909)/Homer | 5.17 |
| Family A | Hypo | 15 | CGTATGACTCGAAGTCCGTAATCGATGCACGTACTGAGTC | TCF3 | E2A(bHLH)/proBcell-E2A-ChIP-Seq(GSE21978)/Homer | 4.69 |
| Family A | Hypo | 16 | ATCGAGCTCTGACTAGACTGACGTGTACGCTAATGCACGTCTAGCATGTACGCGATATGCCGTA | NR1D1 | Reverb(NR),DR2/RAW-Reverba.biotin-ChIP-Seq(GSE45914)/Homer | 4.67 |
| Family A | Hyper | 1 | CATGGTACGACTGCTACGTACGTACGTAGCTAGACTCTGATCAGGTAC | MEF2C | Mef2c(MADS)/GM12878-Mef2c-ChIP-Seq(GSE32465)/Homer | 26.42 |
| Family A | Hyper | 2 | GTACGACTCGTACTGATCGACGTAGCTACAGTCTGATACG | MEF2A | Mef2a(MADS)/HL1-Mef2a.biotin-ChIP-Seq(GSE21529)/Homer | 25.80 |
| Family A | Hyper | 3 | CATGAGTCGACTCGTACGATGCATGACTGCATCGATCTAGCATGTGAC | MEF2B | Mef2b(MADS)/HEK293-Mef2b.V5-ChIP-Seq(GSE67450)/Homer | 21.14 |
| Family A | Hyper | 4 | AGCTGACTCTAGCGTACATGCGATCTAGATCGGACTCAGT | NKX3-2 | Bapx1(Homeobox)/VertebralCol-Bapx1-ChIP-Seq(GSE36672)/Homer | 18.29 |
| Family A | Hyper | 5 | TCAGACGTAGTCTCGAAGTCTCAGGCATCTAGCTAGAGCT | USF2 | Usf2(bHLH)/C2C12-Usf2-ChIP-Seq(GSE36030)/Homer | 14.54 |
| Family A | Hyper | 6 | ATGCGACTAGCTCTAGCGTACTAGCGATCTAGATCGGATC | NKX2-2 | Nkx2.2(Homeobox)/NPC-Nkx2.2-ChIP-Seq(GSE61673)/Homer | 9.68 |
| Family A | Hyper | 7 | CTAGTACGAGTCCGTAAGTCACGTAGTCTCGACGTATACG | NKX2-1 | Nkx2.1(Homeobox)/LungAC-Nkx2.1-ChIP-Seq(GSE43252)/Homer | 8.58 |
| Family A | Hyper | 8 | CATGAGCTTACGGTCAGTACTAGCAGCTGACTATCGTCGA | ESRRB | Esrrb(NR)/mES-Esrrb-ChIP-Seq(GSE11431)/Homer | 8.33 |
| Family A | Hyper | 9 | CTGACTGATAGCGATCGCTAGTACACGTGATCTGCACGTA | NKX2-5 | Nkx2.5(Homeobox)/HL1-Nkx2.5.biotin-ChIP-Seq(GSE21529)/Homer | 8.19 |
| Family A | Hyper | 10 | GATCCTGAAGTCCGATCGATGATCAGTCACTGATCGAGCT | ELK4 | Elk4(ETS)/Hela-Elk4-ChIP-Seq(GSE31477)/Homer | 7.10 |
| Family A | Hyper | 11 | TCAGTCAGTAGCAGTCCTGAAGTCCTAGACGTACTGATCG | MYC | c-Myc(bHLH)/mES-cMyc-ChIP-Seq(GSE11431)/Homer | 7.02 |
| Family A | Hyper | 12 | ACGTCTAGAGCTACGTACGTCTGAAGTCGACTAGCTCGTA | FOXM1 | FOXM1(Forkhead)/MCF7-FOXM1-ChIP-Seq(GSE72977)/Homer | 6.88 |
| Family A | Hyper | 13 | TACGGACTACTGACTGCTAGATGCAGTCAGTCAGTCCTGA | ZNF692 | ZNF692(Zf)/HEK293-ZNF692.GFP-ChIP-Seq(GSE58341)/Homer | 6.84 |
| Family A | Hyper | 14 | TCGATCAGTCGAACTGCATGACGTAGTCCTGA | NR2F2 | COUP-TFII(NR)/Artia-Nr2f2-ChIP-Seq(GSE46497)/Homer | 6.46 |
| Family A | Hyper | 15 | ATCGAGCTCTGACTAGACTGACGTGTACGCTAATGCACGTCTAGCATGTACGCGATATGCCGTA | NR1D1 | Reverb(NR),DR2/RAW-Reverba.biotin-ChIP-Seq(GSE45914)/Homer | 6.35 |
| Family A | Hyper | 16 | GTACCTAGTCAGAGCTTAGCCGTAATGCTACGAGTCGTACGTCAAGTC | SREBF2 | Srebp2(bHLH)/HepG2-Srebp2-ChIP-Seq(GSE31477)/Homer | 6.14 |
| Family A | Hyper | 17 | TCGATGACAGTCCGTAAGTCCTAGACGTACTGACTGAGCTAGTCGCAT | MAX | Max(bHLH)/K562-Max-ChIP-Seq(GSE31477)/Homer | 5.96 |
| Family A | Hyper | 18 | TGACCGTACTGAACTGACTGGACTGATCTGCAGTACTACG | SF1 | SF1(NR)/H295R-Nr5a1-ChIP-Seq(GSE44220)/Homer | 5.54 |
| Family A | Hyper | 19 | ACGTGACTTAGCCGTACTGACATGCTAGGACTGATCCGTA | NR5A2 | Nr5a2(NR)/Pancreas-LRH1-ChIP-Seq(GSE34295)/Homer | 5.41 |
| Family A | Hyper | 20 | ATGCTAGCAGCTAGCTTGACGACTTCAGTACGGTCACTGAATCGTAGCGACTCAGTAGTCAGCTTCGAATCGTGCATGCA | HSF1 | HRE(HSF)/HepG2-HSF1-ChIP-Seq(GSE31477)/Homer | 5.40 |
| Family A | Hyper | 21 | CTGATCGACGTAATGCCGTACGTACGATCTAGTCAGGATC | SOX15 | Sox15(HMG)/CPA-Sox15-ChIP-Seq(GSE62909)/Homer | 4.97 |
| Family A | Hyper | 22 | GCATGCATCTGAACGTCTGAACGTCGTACGTACGTAAGTCGTCAGTCA | FOXF1 | Foxf1(Forkhead)/Lung-Foxf1-ChIP-Seq(GSE77951)/Homer | 4.68 |
| Family A | Hyper | 23 | GCATTCAGCTGAATCGACTGCGATGATCCTGA | THRB | THRb(NR)/Liver-NR1A2-ChIP-Seq(GSE52613)/Homer | 4.66 |
| Family C | Hypo | 1 | GCATGCATCTGAACGTCTGAACGTCGTACGTACGTAAGTCGTCAGTCA | FOXF1 | Foxf1(Forkhead)/Lung-Foxf1-ChIP-Seq(GSE77951)/Homer | 12.57 |
| Family C | Hypo | 2 | CGTAGCTACGATCTAGACGTGTCACGTACGTAAGTCCGTATGCATACG | FOXL2 | FoxL2(Forkhead)/Ovary-FoxL2-ChIP-Seq(GSE60858)/Homer | 11.90 |
| Family C | Hypo | 3 | ACGTCTAGAGCTACGTACGTCTGAAGTCGACTAGCTCGTA | FOXM1 | FOXM1(Forkhead)/MCF7-FOXM1-ChIP-Seq(GSE72977)/Homer | 10.01 |
| Family C | Hypo | 4 | CATGGTACGACTGCTACGTACGTACGTAGCTAGACTCTGATCAGGTAC | MEF2C | Mef2c(MADS)/GM12878-Mef2c-ChIP-Seq(GSE32465)/Homer | 9.77 |
| Family C | Hypo | 5 | AGCTGACTCTAGCGTACATGCGATCTAGATCGGACTCAGT | NKX3-2 | Bapx1(Homeobox)/VertebralCol-Bapx1-ChIP-Seq(GSE36672)/Homer | 9.62 |
| Family C | Hypo | 6 | TCAGACGTAGTCTCGAAGTCTCAGGCATCTAGCTAGAGCT | USF2 | Usf2(bHLH)/C2C12-Usf2-ChIP-Seq(GSE36030)/Homer | 9.57 |
| Family C | Hypo | 7 | GCTATCGACGTACTAGAGCTGTCAGTCACGTAAGTCCGTA | FOXA1 | FOXA1(Forkhead)/MCF7-FOXA1-ChIP-Seq(GSE26831)/Homer | 8.61 |
| Family C | Hypo | 8 | GCTATCGACGTACTAGAGCTGTCAGTCACGTAAGTCCGTA | FOXA1 | FOXA1(Forkhead)/LNCAP-FOXA1-ChIP-Seq(GSE27824)/Homer | 7.53 |
| Family C | Hypo | 9 | GCATATCGCATGGTACGCTAAGTCTCAGTGACGTCATGCA | ARNT, AHR | Arnt:Ahr(bHLH)/MCF7-Arnt-ChIP-Seq(Lo_et_al.)/Homer | 7.51 |
| Family C | Hypo | 10 | TCAGAGCTGTACCGTAACGTCGTACGTACGTAGCTAGACT | CDX2 | Cdx2(Homeobox)/mES-Cdx2-ChIP-Seq(GSE14586)/Homer | 7.35 |
| Family C | Hypo | 11 | ATGCGACTAGCTCTAGCGTACTAGCGATCTAGATCGGATC | NKX2-2 | Nkx2.2(Homeobox)/NPC-Nkx2.2-ChIP-Seq(GSE61673)/Homer | 6.83 |
| Family C | Hypo | 12 | CGATTAGCGACTGTCACGTAACGTCGTACGTACGTAGCTA | HOXD13 | HOXD13(Homeobox)/Chicken-Hoxd13-ChIP-Seq(GSE38910)/Homer | 6.53 |
| Family C | Hypo | 13 | GTACGACTCGTACTGATCGACGTAGCTACAGTCTGATACG | MEF2A | Mef2a(MADS)/HL1-Mef2a.biotin-ChIP-Seq(GSE21529)/Homer | 6.29 |
| Family C | Hypo | 14 | CTAGTACGAGTCCGTAAGTCACGTAGTCTCGACGTATACG | NKX2-1 | Nkx2.1(Homeobox)/LungAC-Nkx2.1-ChIP-Seq(GSE43252)/Homer | 6.02 |
| Family C | Hypo | 15 | CGATACGTACGTACGTCGTAAGCTCAGTCTAGATCGACTG | HOXB13 | HOXB13(Homeobox)/ProstateTumor-HOXB13-ChIP-Seq(GSE56288)/Homer | 5.55 |
| Family C | Hypo | 16 | GCTAACGTCTAGGTACGCTAGACTCTGAGCATCATGGATC | POU1F1 | Pit1(Homeobox)/GCrat-Pit1-ChIP-Seq(GSE58009)/Homer | 4.99 |
| Family C | Hypo | 17 | CATGAGTCGACTCGTACGATGCATGACTGCATCGATCTAGCATGTGAC | MEF2B | Mef2b(MADS)/HEK293-Mef2b.V5-ChIP-Seq(GSE67450)/Homer | 4.72 |
| Family C | Hypo | 18 | TGCACGTAGTCAAGCTAGTCGCTATAGCCGATCTAGGATC | GFI1B | Gfi1b(Zf)/HPC7-Gfi1b-ChIP-Seq(GSE22178)/Homer | 4.70 |
| Family C | Hyper | 1 | CATGGTACGACTGCTACGTACGTACGTAGCTAGACTCTGATCAGGTAC | MEF2C | Mef2c(MADS)/GM12878-Mef2c-ChIP-Seq(GSE32465)/Homer | 22.11 |
| Family C | Hyper | 2 | AGCTGACTCTAGCGTACATGCGATCTAGATCGGACTCAGT | NKX3-2 | Bapx1(Homeobox)/VertebralCol-Bapx1-ChIP-Seq(GSE36672)/Homer | 19.74 |
| Family C | Hyper | 3 | TCAGACGTAGTCTCGAAGTCTCAGGCATCTAGCTAGAGCT | USF2 | Usf2(bHLH)/C2C12-Usf2-ChIP-Seq(GSE36030)/Homer | 16.78 |
| Family C | Hyper | 4 | GTACGACTCGTACTGATCGACGTAGCTACAGTCTGATACG | MEF2A | Mef2a(MADS)/HL1-Mef2a.biotin-ChIP-Seq(GSE21529)/Homer | 16.38 |
| Family C | Hyper | 5 | CATGAGTCGACTCGTACGATGCATGACTGCATCGATCTAGCATGTGAC | MEF2B | Mef2b(MADS)/HEK293-Mef2b.V5-ChIP-Seq(GSE67450)/Homer | 13.09 |
| Family C | Hyper | 6 | GATCTCGAAGTCCGATCGATAGTCATGCACTGATCGGACT | ELK1 | Elk1(ETS)/Hela-Elk1-ChIP-Seq(GSE31477)/Homer | 11.99 |
| Family C | Hyper | 7 | GATCCTGAAGTCCGATCGATGATCAGTCACTGATCGAGCT | ELK4 | Elk4(ETS)/Hela-Elk4-ChIP-Seq(GSE31477)/Homer | 11.53 |
| Family C | Hyper | 8 | ATGCGACTAGCTCTAGCGTACTAGCGATCTAGATCGGATC | NKX2-2 | Nkx2.2(Homeobox)/NPC-Nkx2.2-ChIP-Seq(GSE61673)/Homer | 8.19 |
| Family C | Hyper | 9 | CTGACAGTCTGAAGTCCTAGGACTATCGGTAC | ARNT | HIF-1b(HLH)/T47D-HIF1b-ChIP-Seq(GSE59937)/Homer | 7.79 |
| Family C | Hyper | 10 | TCGATCAGTCGAACTGCATGACGTAGTCCTGA | NR2F2 | COUP-TFII(NR)/Artia-Nr2f2-ChIP-Seq(GSE46497)/Homer | 7.47 |
| Family C | Hyper | 11 | GTACCTAGTCAGAGCTTAGCCGTAATGCTACGAGTCGTACGTCAAGTC | SREBF2 | Srebp2(bHLH)/HepG2-Srebp2-ChIP-Seq(GSE31477)/Homer | 7.24 |
| Family C | Hyper | 12 | TCGAGCATATGCCTGAATGCTAGCAGTCGTACTCGAAGCT | SREBF1 | Srebp1a(bHLH)/HepG2-Srebp1a-ChIP-Seq(GSE31477)/Homer | 6.65 |
| Family C | Hyper | 13 | TCAGTCAGTAGCAGTCCTGAAGTCCTAGACGTACTGATCG | MYC | c-Myc(bHLH)/mES-cMyc-ChIP-Seq(GSE11431)/Homer | 6.46 |
| Family C | Hyper | 14 | CGTACTAGGACTGTCAGTCACGTAAGTCCGTATCGATCGATCGACGTACTGACTAGGCTACGTATAGCCGTACGATCGTA | FOXA1 | FOXA1:AR(Forkhead,NR)/LNCAP-AR-ChIP-Seq(GSE27824)/Homer | 6.26 |
| Family C | Hyper | 15 | TACGTCGATAGCAGTCCGTAAGTCCTAGGCATACTGATCG | MYCN | n-Myc(bHLH)/mES-nMyc-ChIP-Seq(GSE11431)/Homer | 6.25 |
| Family C | Hyper | 16 | AGTCCTGAAGTCCGATCAGTGATCATGCACTGATCGGACT | FLI1 | Fli1(ETS)/CD8-FLI-ChIP-Seq(GSE20898)/Homer | 5.95 |
| Family C | Hyper | 17 | CTGACTGATAGCGATCGCTAGTACACGTGATCTGCACGTA | NKX2-5 | Nkx2.5(Homeobox)/HL1-Nkx2.5.biotin-ChIP-Seq(GSE21529)/Homer | 5.92 |
| Family C | Hyper | 18 | CGTAACTGGTCAACGTATCGCAGTCTAGTCAGCGTAACTGCGTAACGTCGTACTGATACG | GATA3 | GATA3(Zf),DR4/iTreg-Gata3-ChIP-Seq(GSE20898)/Homer | 5.62 |
| Family C | Hyper | 19 | CTAGCATGACGTAGTCGCTAAGCTAGTCAGCTTCAGCTGAACTGCATGGCATATGCCGTA | THRA | THRa(NR)/C17.2-THRa-ChIP-Seq(GSE38347)/Homer | 5.48 |
| Family C | Hyper | 20 | TCGATGACAGTCCGTAAGTCCTAGACGTACTGACTGAGCTAGTCGCAT | MAX | Max(bHLH)/K562-Max-ChIP-Seq(GSE31477)/Homer | 5.30 |
| Family C | Hyper | 21 | ACGTCTAGAGCTACGTACGTCTGAAGTCGACTAGCTCGTA | FOXM1 | FOXM1(Forkhead)/MCF7-FOXM1-ChIP-Seq(GSE72977)/Homer | 5.25 |
| Family C | Hyper | 22 | CTAGTACGAGTCCGTAAGTCACGTAGTCTCGACGTATACG | NKX2-1 | Nkx2.1(Homeobox)/LungAC-Nkx2.1-ChIP-Seq(GSE43252)/Homer | 4.91 |
| Family C | Hyper | 23 | ATCGAGCTCTGACTAGACTGACGTGTACGCTAATGCACGTCTAGCATGTACGCGATATGCCGTA | NR1D1 | Reverb(NR),DR2/RAW-Reverba.biotin-ChIP-Seq(GSE45914)/Homer | 4.91 |
| Family C | Hyper | 24 | CTGATCGACGTAATGCCGTACGTACGATCTAGTCAGGATC | SOX15 | Sox15(HMG)/CPA-Sox15-ChIP-Seq(GSE62909)/Homer | 4.79 |
| Family C | Hyper | 25 | AGTCATCGGCATCTAGACTGTACGCGATTCAGCATGAGCTTAGCGATC | GLI3 | GLI3(Zf)/Limb-GLI3-ChIP-Chip(GSE11077)/Homer | 4.62 |
| Family C | Hyper | 26 | CATGGTACCGTAAGTCCTAGACGTACTGGTACAGTCAGCT | BHLH40E | bHLHE40(bHLH)/HepG2-BHLHE40-ChIP-Seq(GSE31477)/Homer | 4.61 |
| Shared | Hypo | 1 | TGCAAGCTACGTCTAGGATCCTAGGATCGTCACTGAAGTC | CEBPB | CEBP(bZIP)/ThioMac-CEBPb-ChIP-Seq(GSE21512)/Homer | 5.68 |
| Shared | Hypo | 2 | TGCAACTGTACGATGCAGTCGACTTCGAATCG | ZNF711 | ZNF711(Zf)/SHSY5Y-ZNF711-ChIP-Seq(GSE20673)/Homer | 5.41 |
| Shared | Hypo | 3 | CGTATGACTCGAAGTCCGTAATCGATGCACGTACTGAGTC | TCF3 | E2A(bHLH)/proBcell-E2A-ChIP-Seq(GSE21978)/Homer | 5.19 |
| Shared | Hyper | 1 | GCTATCGACGTACTAGAGCTGTCAGTCACGTAAGTCCGTA | FOXA1 | FOXA1(Forkhead)/LNCAP-FOXA1-ChIP-Seq(GSE27824)/Homer | 5.85 |
| Shared | Hyper | 2 | GCTATCGACGTACTAGAGCTGTCAGTCACGTAAGTCCGTA | FOXA1 | FOXA1(Forkhead)/MCF7-FOXA1-ChIP-Seq(GSE26831)/Homer | 4.93 |
| Shared | Hyper | 3 | GCATGCATCTGAACGTCTGAACGTCGTACGTACGTAAGTCGTCAGTCA | FOXF1 | Foxf1(Forkhead)/Lung-Foxf1-ChIP-Seq(GSE77951)/Homer | 4.87 |

(*) Significance of the motif is displayed in the last column as -log(p-value), calculated using the hypergeometric test through HOMER^1^.

**Abbreviations:** DMR, differentially methylated region; Hyper, hypermethylated; Hypo, hypomethylated; TF, transcription factor; TFBS, transcription factor binding site

**Table S7**

**Complete list of disease ontology terms from ToppGene for gene lists associated with either hypo or hypermethylated fibroblast DMR contexts**

| **DMR group** | **DMR type** | **Rank** | **ID*** | **Name** | **Source** | **p-value†** | **FDR B&H‡** |
| --- | --- | --- | --- | --- | --- | --- | --- |
| Shared | Hyper | 1 | C0014544 | Epilepsy | DisGeNET BeFree | 1.43E-06 | 1.04E-02 |
| Shared | Hyper | 2 | C1535926 | Neurodevelopmental Disorders | DisGeNET Curated | 3.30E-06 | 1.20E-02 |
| Shared | Hyper | 3 | 20090507:Lasky-Su | Hyperactive-impulsive symptoms | GWAS | 8.74E-06 | 2.12E-02 |
| Shared | Hyper | 4 | C0086743 | Osteoarthrosis Deformans | DisGeNET Curated | 1.81E-05 | 2.25E-02 |
| Shared | Hyper | 5 | C0029408 | Degenerative polyarthritis | DisGeNET Curated | 1.81E-05 | 2.25E-02 |
| Shared | Hyper | 6 | C3714756 | Intellectual Disability | DisGeNET BeFree | 1.86E-05 | 2.25E-02 |
| Shared | Hypo | - | - | None | - | - | - |
| Family A | Hyper | 1 | C0028754 | Obesity | DisGeNET BeFree | 1.50E-09 | 1.65E-05 |
| Family A | Hyper | 2 | C0014544 | Epilepsy | DisGeNET BeFree | 3.12E-09 | 1.65E-05 |
| Family A | Hyper | 3 | C0036341 | Schizophrenia | DisGeNET BeFree | 3.86E-09 | 1.65E-05 |
| Family A | Hyper | 4 | C0001418 | Adenocarcinoma | DisGeNET BeFree | 6.22E-09 | 1.65E-05 |
| Family A | Hyper | 5 | C0278878 | Adult Glioblastoma | DisGeNET BeFree | 6.57E-09 | 1.65E-05 |
| Family A | Hyper | 6 | C0280474 | Childhood Glioblastoma | DisGeNET BeFree | 6.57E-09 | 1.65E-05 |
| Family A | Hyper | 7 | C0027765 | nervous system disorder | DisGeNET BeFree | 6.76E-09 | 1.65E-05 |
| Family A | Hyper | 8 | C0338656 | Impaired cognition | DisGeNET BeFree | 1.69E-08 | 3.62E-05 |
| Family A | Hyper | 9 | C0007758 | Cerebellar Ataxia | DisGeNET BeFree | 3.57E-08 | 6.78E-05 |
| Family A | Hyper | 10 | C0699790 | Colon Carcinoma | DisGeNET BeFree | 4.71E-08 | 8.05E-05 |
| Family A | Hyper | 11 | C3714756 | Intellectual Disability | DisGeNET BeFree | 5.29E-08 | 8.21E-05 |
| Family A | Hyper | 12 | C1535926 | Neurodevelopmental Disorders | DisGeNET BeFree | 8.99E-08 | 1.28E-04 |
| Family A | Hyper | 13 | C0001973 | Alcoholic Intoxication, Chronic | DisGeNET Curated | 9.80E-08 | 1.29E-04 |
| Family A | Hyper | 14 | C0007102 | Malignant tumor of colon | DisGeNET BeFree | 2.44E-07 | 2.98E-04 |
| Family A | Hyper | 15 | C1510586 | Autism Spectrum Disorders | DisGeNET BeFree | 3.50E-07 | 3.98E-04 |
| Family A | Hyper | 16 | C0344315 | Depressed mood | DisGeNET BeFree | 4.49E-07 | 4.80E-04 |
| Family A | Hyper | 17 | C0009319 | Colitis | DisGeNET BeFree | 5.35E-07 | 5.38E-04 |
| Family A | Hyper | 18 | C0011570 | Mental Depression | DisGeNET BeFree | 7.66E-07 | 7.27E-04 |
| Family A | Hyper | 19 | C0007097 | Carcinoma | DisGeNET BeFree | 9.95E-07 | 8.95E-04 |
| Family A | Hyper | 20 | C0025286 | Meningioma | DisGeNET BeFree | 1.09E-06 | 9.32E-04 |
| Family A | Hyper | 21 | C0011581 | Depressive disorder | DisGeNET BeFree | 1.17E-06 | 9.48E-04 |
| Family A | Hyper | 22 | C0013384 | Dyskinetic syndrome | DisGeNET BeFree | 1.22E-06 | 9.48E-04 |
| Family A | Hyper | 23 | C0011849 | Diabetes Mellitus | DisGeNET BeFree | 1.95E-06 | 1.45E-03 |
| Family A | Hyper | 24 | C0085281 | Addictive Behavior | DisGeNET BeFree | 2.33E-06 | 1.66E-03 |
| Family A | Hyper | 25 | C0002736 | Amyotrophic Lateral Sclerosis | DisGeNET BeFree | 2.92E-06 | 2.00E-03 |
| Family A | Hyper | 26 | C0557874 | Global developmental delay | DisGeNET BeFree | 4.01E-06 | 2.61E-03 |
| Family A | Hyper | 27 | C0007785 | Cerebral Infarction | DisGeNET BeFree | 4.12E-06 | 2.61E-03 |
| Family A | Hyper | 28 | C0011847 | Diabetes | DisGeNET BeFree | 4.39E-06 | 2.68E-03 |
| Family A | Hyper | 29 | C0042769 | Virus Diseases | DisGeNET BeFree | 4.78E-06 | 2.82E-03 |
| Family A | Hyper | 30 | C0524851 | Neurodegenerative Disorders | DisGeNET BeFree | 6.01E-06 | 3.42E-03 |
| Family A | Hyper | 31 | C0005586 | Bipolar Disorder | DisGeNET Curated | 8.40E-06 | 4.47E-03 |
| Family A | Hyper | 32 | C0006142 | Malignant neoplasm of breast | DisGeNET Curated | 8.73E-06 | 4.47E-03 |
| Family A | Hyper | 33 | C0026764 | Multiple Myeloma | DisGeNET BeFree | 8.76E-06 | 4.47E-03 |
| Family A | Hyper | 34 | C1458155 | Mammary Neoplasms | DisGeNET BeFree | 8.89E-06 | 4.47E-03 |
| Family A | Hyper | 35 | C0424605 | Developmental delay (disorder) | DisGeNET BeFree | 9.50E-06 | 4.64E-03 |
| Family A | Hyper | 36 | C0000768 | Congenital Abnormality | DisGeNET BeFree | 1.11E-05 | 5.25E-03 |
| Family A | Hyper | 37 | C0010054 | Coronary Arteriosclerosis | DisGeNET BeFree | 1.18E-05 | 5.45E-03 |
| Family A | Hyper | 38 | C1389018 | Atrioventricular Septal Defect | DisGeNET BeFree | 1.27E-05 | 5.69E-03 |
| Family A | Hyper | 39 | C0271650 | Impaired glucose tolerance | DisGeNET BeFree | 1.31E-05 | 5.75E-03 |
| Family A | Hyper | 40 | C0278877 | Adult Meningioma | DisGeNET BeFree | 1.43E-05 | 5.99E-03 |
| Family A | Hyper | 41 | C0036341 | Schizophrenia | DisGeNET Curated | 1.48E-05 | 5.99E-03 |
| Family A | Hyper | 42 | C0026838 | Muscle Spasticity | DisGeNET BeFree | 1.52E-05 | 5.99E-03 |
| Family A | Hyper | 43 | C0006118 | Brain Neoplasms | DisGeNET BeFree | 1.54E-05 | 5.99E-03 |
| Family A | Hyper | 44 | C0025202 | melanoma | DisGeNET BeFree | 1.54E-05 | 5.99E-03 |
| Family A | Hyper | 45 | C0030193 | Pain | DisGeNET BeFree | 1.64E-05 | 6.21E-03 |
| Family A | Hyper | 46 | C1328504 | Hormone refractory prostate cancer | DisGeNET BeFree | 1.78E-05 | 6.63E-03 |
| Family A | Hyper | 47 | C0007959 | Charcot-Marie-Tooth Disease | DisGeNET BeFree | 1.96E-05 | 7.12E-03 |
| Family A | Hyper | 48 | C0010068 | Coronary heart disease | DisGeNET BeFree | 2.11E-05 | 7.53E-03 |
| Family A | Hyper | 49 | C1762616 | Meningioma, benign, no ICD-O subtype | DisGeNET BeFree | 2.30E-05 | 8.01E-03 |
| Family A | Hyper | 50 | C0004936 | Mental disorders | DisGeNET BeFree | 2.41E-05 | 8.11E-03 |
| Family A | Hyper | 51 | C0234958 | Muscle degeneration | DisGeNET BeFree | 2.42E-05 | 8.11E-03 |
| Family A | Hyper | 52 | C0025958 | Microcephaly | DisGeNET BeFree | 2.65E-05 | 8.57E-03 |
| Family A | Hyper | 53 | C0019569 | Hirschsprung Disease | DisGeNET BeFree | 2.77E-05 | 8.57E-03 |
| Family A | Hyper | 54 | C1611743 | Familial (FPAH) | DisGeNET BeFree | 2.85E-05 | 8.57E-03 |
| Family A | Hyper | 55 | C3539878 | Triple Negative Breast Neoplasms | DisGeNET BeFree | 2.89E-05 | 8.57E-03 |
| Family A | Hyper | 56 | C0153690 | Secondary malignant neoplasm of bone | DisGeNET BeFree | 3.15E-05 | 8.57E-03 |
| Family A | Hyper | 57 | C0700095 | Central neuroblastoma | DisGeNET BeFree | 3.24E-05 | 8.57E-03 |
| Family A | Hyper | 58 | C4316881 | Prescription Drug Abuse | DisGeNET Curated | 3.34E-05 | 8.57E-03 |
| Family A | Hyper | 59 | C0013170 | Drug habituation | DisGeNET Curated | 3.34E-05 | 8.57E-03 |
| Family A | Hyper | 60 | C0013146 | Drug abuse | DisGeNET Curated | 3.34E-05 | 8.57E-03 |
| Family A | Hyper | 61 | C0013222 | Drug Use Disorders | DisGeNET Curated | 3.34E-05 | 8.57E-03 |
| Family A | Hyper | 62 | C0038580 | Substance Dependence | DisGeNET Curated | 3.34E-05 | 8.57E-03 |
| Family A | Hyper | 63 | C0038586 | Substance Use Disorders | DisGeNET Curated | 3.34E-05 | 8.57E-03 |
| Family A | Hyper | 64 | C0236969 | Substance-Related Disorders | DisGeNET Curated | 3.34E-05 | 8.57E-03 |
| Family A | Hyper | 65 | C1510472 | Drug Dependence | DisGeNET Curated | 3.34E-05 | 8.57E-03 |
| Family A | Hyper | 66 | C0029231 | Organic Mental Disorders, Substance-Induced | DisGeNET Curated | 3.34E-05 | 8.57E-03 |
| Family A | Hyper | 67 | C4086165 | Childhood Neuroblastoma | DisGeNET BeFree | 3.36E-05 | 8.57E-03 |
| Family A | Hyper | 68 | C0027819 | Neuroblastoma | DisGeNET BeFree | 3.45E-05 | 8.67E-03 |
| Family A | Hyper | 69 | C0019348 | Herpes Simplex Infections | DisGeNET BeFree | 3.66E-05 | 9.06E-03 |
| Family A | Hyper | 70 | C0023418 | leukemia | DisGeNET BeFree | 3.75E-05 | 9.15E-03 |
| Family A | Hyper | 71 | C0740858 | Substance abuse problem | DisGeNET Curated | 4.02E-05 | 9.67E-03 |
| Family A | Hyper | 72 | C0030567 | Parkinson Disease | DisGeNET BeFree | 4.08E-05 | 9.67E-03 |
| Family A | Hyper | 73 | C1561643 | Chronic Kidney Diseases | DisGeNET BeFree | 4.79E-05 | 1.12E-02 |
| Family A | Hyper | 74 | C0004352 | Autistic Disorder | DisGeNET BeFree | 4.99E-05 | 1.15E-02 |
| Family A | Hyper | 75 | C0153676 | Secondary malignant neoplasm of lung | DisGeNET BeFree | 5.04E-05 | 1.15E-02 |
| Family A | Hyper | 76 | C0020429 | Hyperalgesia | DisGeNET BeFree | 5.15E-05 | 1.16E-02 |
| Family A | Hyper | 77 | C0020538 | Hypertensive disease | DisGeNET BeFree | 5.49E-05 | 1.22E-02 |
| Family A | Hyper | 78 | C0598766 | Leukemogenesis | DisGeNET BeFree | 5.68E-05 | 1.24E-02 |
| Family A | Hyper | 79 | C0017638 | Glioma | DisGeNET BeFree | 5.77E-05 | 1.25E-02 |
| Family A | Hyper | 80 | C0008073 | Developmental Disabilities | DisGeNET BeFree | 6.13E-05 | 1.30E-02 |
| Family A | Hyper | 81 | C0003873 | Rheumatoid Arthritis | DisGeNET BeFree | 6.14E-05 | 1.30E-02 |
| Family A | Hyper | 82 | C0011860 | Diabetes Mellitus, Non-Insulin-Dependent | DisGeNET BeFree | 6.35E-05 | 1.32E-02 |
| Family A | Hyper | 83 | C0036572 | Seizures | DisGeNET BeFree | 6.93E-05 | 1.42E-02 |
| Family A | Hyper | 84 | C0376634 | Craniofacial Abnormalities | DisGeNET Curated | 7.00E-05 | 1.42E-02 |
| Family A | Hyper | 85 | C0019340 | Herpes NOS | DisGeNET BeFree | 7.09E-05 | 1.43E-02 |
| Family A | Hyper | 86 | C0020179 | Huntington Disease | DisGeNET BeFree | 8.73E-05 | 1.73E-02 |
| Family A | Hyper | 87 | C1332977 | Childhood Leukemia | DisGeNET BeFree | 8.99E-05 | 1.77E-02 |
| Family A | Hyper | 88 | C4722518 | Triple-Negative Breast Carcinoma | DisGeNET BeFree | 9.11E-05 | 1.77E-02 |
| Family A | Hyper | 89 | C0040822 | Tremor | DisGeNET BeFree | 9.26E-05 | 1.78E-02 |
| Family A | Hyper | 90 | C0004134 | Ataxia | DisGeNET BeFree | 1.10E-04 | 2.09E-02 |
| Family A | Hyper | 91 | C0233514 | Abnormal behavior | DisGeNET BeFree | 1.16E-04 | 2.16E-02 |
| Family A | Hyper | 92 | C0598589 | Inherited neuropathies | DisGeNET BeFree | 1.17E-04 | 2.16E-02 |
| Family A | Hyper | 93 | C0009241 | Cognition Disorders | DisGeNET BeFree | 1.19E-04 | 2.19E-02 |
| Family A | Hyper | 94 | C0037763 | Spasm | DisGeNET BeFree | 1.27E-04 | 2.28E-02 |
| Family A | Hyper | 95 | C0235974 | Pancreatic carcinoma | DisGeNET BeFree | 1.27E-04 | 2.28E-02 |
| Family A | Hyper | 96 | C0014175 | Endometriosis | DisGeNET BeFree | 1.30E-04 | 2.28E-02 |
| Family A | Hyper | 97 | C0278595 | Adult Fibrosarcoma | DisGeNET BeFree | 1.30E-04 | 2.28E-02 |
| Family A | Hyper | 98 | C0025149 | Medulloblastoma | DisGeNET BeFree | 1.38E-04 | 2.41E-02 |
| Family A | Hyper | 99 | C3266262 | Multiple Chronic Conditions | DisGeNET BeFree | 1.52E-04 | 2.62E-02 |
| Family A | Hyper | 100 | C0023467 | Leukemia, Myelocytic, Acute | DisGeNET BeFree | 1.53E-04 | 2.62E-02 |
| Family A | Hyper | 101 | C2677180 | Congenital microcephaly | DisGeNET BeFree | 1.59E-04 | 2.68E-02 |
| Family A | Hyper | 102 | C0858600 | Taste sweet | DisGeNET BeFree | 1.60E-04 | 2.68E-02 |
| Family A | Hyper | 103 | C0026650 | Movement Disorders | DisGeNET BeFree | 1.65E-04 | 2.69E-02 |
| Family A | Hyper | 104 | C0027868 | Neuromuscular Diseases | DisGeNET BeFree | 1.66E-04 | 2.69E-02 |
| Family A | Hyper | 105 | C0029408 | Degenerative polyarthritis | DisGeNET BeFree | 1.67E-04 | 2.69E-02 |
| Family A | Hyper | 106 | C1956346 | Coronary Artery Disease | DisGeNET BeFree | 1.67E-04 | 2.69E-02 |
| Family A | Hyper | 107 | C0023449 | Acute lymphocytic leukemia | DisGeNET BeFree | 1.69E-04 | 2.70E-02 |
| Family A | Hyper | 108 | C0346647 | Malignant neoplasm of pancreas | DisGeNET BeFree | 1.71E-04 | 2.70E-02 |
| Family A | Hyper | 109 | C0042063 | Urogenital Abnormalities | DisGeNET BeFree | 1.80E-04 | 2.82E-02 |
| Family A | Hyper | 110 | C0014544 | Epilepsy | DisGeNET Curated | 1.81E-04 | 2.82E-02 |
| Family A | Hyper | 111 | C0023434 | Chronic Lymphocytic Leukemia | DisGeNET BeFree | 1.84E-04 | 2.84E-02 |
| Family A | Hyper | 112 | C0016057 | Fibrosarcoma | DisGeNET BeFree | 1.95E-04 | 2.97E-02 |
| Family A | Hyper | 113 | C0085220 | Cerebral Amyloid Angiopathy | DisGeNET BeFree | 2.09E-04 | 3.16E-02 |
| Family A | Hyper | 114 | C0280222 | stage, pancreatic cancer | DisGeNET BeFree | 2.15E-04 | 3.17E-02 |
| Family A | Hyper | 115 | C1842937 | AURAL ATRESIA, CONGENITAL | DisGeNET BeFree | 2.15E-04 | 3.17E-02 |
| Family A | Hyper | 116 | C0004352 | Autistic Disorder | DisGeNET Curated | 2.15E-04 | 3.17E-02 |
| Family A | Hyper | 117 | C0086438 | Hypogammaglobulinemia | DisGeNET BeFree | 2.22E-04 | 3.25E-02 |
| Family A | Hyper | 118 | C0027794 | Neural Tube Defects | DisGeNET BeFree | 2.27E-04 | 3.28E-02 |
| Family A | Hyper | 119 | C0162809 | Kallmann Syndrome | DisGeNET BeFree | 2.32E-04 | 3.30E-02 |
| Family A | Hyper | 120 | cv: | Progressive myoclonus epilepsy | Clinical Variations | 2.32E-04 | 3.30E-02 |
| Family A | Hyper | 121 | C0027819 | Neuroblastoma | DisGeNET Curated | 2.57E-04 | 3.63E-02 |
| Family A | Hyper | 122 | C0917981 | Progressive Muscular Atrophy | DisGeNET BeFree | 2.77E-04 | 3.88E-02 |
| Family A | Hyper | 123 | C0001973 | Alcoholic Intoxication, Chronic | DisGeNET BeFree | 2.90E-04 | 4.03E-02 |
| Family A | Hyper | 124 | C0021841 | Intestinal Neoplasms | DisGeNET BeFree | 3.00E-04 | 4.13E-02 |
| Family A | Hyper | 125 | C0021390 | Inflammatory Bowel Diseases | DisGeNET BeFree | 3.02E-04 | 4.13E-02 |
| Family A | Hyper | 126 | C0021141 | Inappropriate ADH Syndrome | DisGeNET BeFree | 3.31E-04 | 4.35E-02 |
| Family A | Hyper | 127 | C0017178 | Gastrointestinal Diseases | DisGeNET Curated | 3.31E-04 | 4.35E-02 |
| Family A | Hyper | 128 | C0559031 | Functional Gastrointestinal Disorders | DisGeNET Curated | 3.31E-04 | 4.35E-02 |
| Family A | Hyper | 129 | C1565321 | Cholera Infantum | DisGeNET Curated | 3.31E-04 | 4.35E-02 |
| Family A | Hyper | 130 | C0023440 | Acute Erythroblastic Leukemia | DisGeNET BeFree | 3.31E-04 | 4.35E-02 |
| Family A | Hyper | 131 | C0023467 | Leukemia, Myelocytic, Acute | DisGeNET Curated | 3.40E-04 | 4.44E-02 |
| Family A | Hypo | 1 | C0004352 | Autistic Disorder | DisGeNET BeFree | 6.27E-06 | 3.62E-02 |
| Family A | Hypo | 2 | C3854173 | Pre-renal acute kidney injury | DisGeNET BeFree | 1.30E-05 | 3.62E-02 |
| Family A | Hypo | 3 | C0011581 | Depressive disorder | DisGeNET BeFree | 1.31E-05 | 3.62E-02 |
| Family A | Hypo | 4 | C1510586 | Autism Spectrum Disorders | DisGeNET BeFree | 1.57E-05 | 3.62E-02 |
| Family A | Hypo | 5 | C0344315 | Depressed mood | DisGeNET BeFree | 1.72E-05 | 3.62E-02 |
| Family A | Hypo | 6 | C0011570 | Mental Depression | DisGeNET BeFree | 1.75E-05 | 3.62E-02 |
| Family C | Hyper | 1 | C0036341 | Schizophrenia | DisGeNET BeFree | 1.16E-11 | 1.89E-07 |
| Family C | Hyper | 2 | C1510586 | Autism Spectrum Disorders | DisGeNET BeFree | 3.05E-09 | 1.86E-05 |
| Family C | Hyper | 3 | C0000768 | Congenital Abnormality | DisGeNET BeFree | 3.42E-09 | 1.86E-05 |
| Family C | Hyper | 4 | C0011581 | Depressive disorder | DisGeNET BeFree | 1.39E-08 | 5.67E-05 |
| Family C | Hyper | 5 | C0557874 | Global developmental delay | DisGeNET BeFree | 2.01E-08 | 6.54E-05 |
| Family C | Hyper | 6 | C0036341 | Schizophrenia | DisGeNET Curated | 2.62E-08 | 7.12E-05 |
| Family C | Hyper | 7 | C0424295 | Hyperactive behavior | DisGeNET BeFree | 5.03E-08 | 1.14E-04 |
| Family C | Hyper | 8 | C0344315 | Depressed mood | DisGeNET BeFree | 5.59E-08 | 1.14E-04 |
| Family C | Hyper | 9 | C0011570 | Mental Depression | DisGeNET BeFree | 1.12E-07 | 1.83E-04 |
| Family C | Hyper | 10 | C0424605 | Developmental delay (disorder) | DisGeNET BeFree | 1.12E-07 | 1.83E-04 |
| Family C | Hyper | 11 | C0338656 | Impaired cognition | DisGeNET BeFree | 1.65E-07 | 2.45E-04 |
| Family C | Hyper | 12 | C0004352 | Autistic Disorder | DisGeNET BeFree | 2.08E-07 | 2.80E-04 |
| Family C | Hyper | 13 | C0221357 | Brachydactyly | DisGeNET BeFree | 2.23E-07 | 2.80E-04 |
| Family C | Hyper | 14 | C0020456 | Hyperglycemia | DisGeNET BeFree | 2.64E-07 | 3.08E-04 |
| Family C | Hyper | 15 | C0376634 | Craniofacial Abnormalities | DisGeNET Curated | 4.63E-07 | 5.03E-04 |
| Family C | Hyper | 16 | C0028754 | Obesity | DisGeNET BeFree | 1.14E-06 | 1.16E-03 |
| Family C | Hyper | 17 | C0524528 | Pervasive Development Disorder | DisGeNET BeFree | 1.77E-06 | 1.70E-03 |
| Family C | Hyper | 18 | C0030193 | Pain | DisGeNET BeFree | 2.74E-06 | 2.47E-03 |
| Family C | Hyper | 19 | C1269683 | Major Depressive Disorder | DisGeNET BeFree | 2.88E-06 | 2.47E-03 |
| Family C | Hyper | 20 | C0030567 | Parkinson Disease | DisGeNET BeFree | 3.99E-06 | 3.25E-03 |
| Family C | Hyper | 21 | C0018798 | Congenital Heart Defects | DisGeNET BeFree | 4.93E-06 | 3.83E-03 |
| Family C | Hyper | 22 | C0036572 | Seizures | DisGeNET BeFree | 7.94E-06 | 5.84E-03 |
| Family C | Hyper | 23 | C0041696 | Unipolar Depression | DisGeNET BeFree | 8.24E-06 | 5.84E-03 |
| Family C | Hyper | 24 | C0003467 | Anxiety | DisGeNET BeFree | 9.23E-06 | 6.27E-03 |
| Family C | Hyper | 25 | C0302142 | Deformity | DisGeNET BeFree | 1.03E-05 | 6.72E-03 |
| Family C | Hyper | 26 | C0020676 | Hypothyroidism | DisGeNET BeFree | 1.08E-05 | 6.75E-03 |
| Family C | Hyper | 27 | C0011269 | Dementia, Vascular | DisGeNET BeFree | 1.12E-05 | 6.75E-03 |
| Family C | Hyper | 28 | C0033975 | Psychotic Disorders | DisGeNET BeFree | 1.16E-05 | 6.75E-03 |
| Family C | Hyper | 29 | C0025286 | Meningioma | DisGeNET BeFree | 1.23E-05 | 6.90E-03 |
| Family C | Hyper | 30 | C1535926 | Neurodevelopmental Disorders | DisGeNET BeFree | 1.47E-05 | 8.01E-03 |
| Family C | Hyper | 31 | C0349204 | Nonorganic psychosis | DisGeNET BeFree | 1.53E-05 | 8.03E-03 |
| Family C | Hyper | 32 | C0027765 | nervous system disorder | DisGeNET BeFree | 1.64E-05 | 8.37E-03 |
| Family C | Hyper | 33 | C0008073 | Developmental Disabilities | DisGeNET BeFree | 1.81E-05 | 8.96E-03 |
| Family C | Hyper | 34 | C0233514 | Abnormal behavior | DisGeNET BeFree | 2.42E-05 | 1.16E-02 |
| Family C | Hyper | 35 | C0003469 | Anxiety Disorders | DisGeNET BeFree | 2.48E-05 | 1.16E-02 |
| Family C | Hyper | 36 | C0001973 | Alcoholic Intoxication, Chronic | DisGeNET Curated | 2.67E-05 | 1.17E-02 |
| Family C | Hyper | 37 | C3714756 | Intellectual Disability | DisGeNET BeFree | 2.72E-05 | 1.17E-02 |
| Family C | Hyper | 38 | C0011860 | Diabetes Mellitus, Non-Insulin-Dependent | DisGeNET BeFree | 2.73E-05 | 1.17E-02 |
| Family C | Hyper | 39 | C1321551 | Shprintzen-Goldberg syndrome | DisGeNET BeFree | 2.82E-05 | 1.18E-02 |
| Family C | Hyper | 40 | C0236733 | Amphetamine-Related Disorders | DisGeNET Curated | 3.35E-05 | 1.30E-02 |
| Family C | Hyper | 41 | C0236807 | Amphetamine Abuse | DisGeNET Curated | 3.35E-05 | 1.30E-02 |
| Family C | Hyper | 42 | C0236804 | Amphetamine Addiction | DisGeNET Curated | 3.35E-05 | 1.30E-02 |
| Family C | Hyper | 43 | C0278878 | Adult Glioblastoma | DisGeNET BeFree | 3.66E-05 | 1.36E-02 |
| Family C | Hyper | 44 | C0280474 | Childhood Glioblastoma | DisGeNET BeFree | 3.66E-05 | 1.36E-02 |
| Family C | Hyper | 45 | C0877015 | Pelvic Organ Prolapse | DisGeNET BeFree | 3.78E-05 | 1.37E-02 |
| Family C | Hyper | 46 | C3714796 | Isolated somatotropin deficiency | DisGeNET BeFree | 4.08E-05 | 1.45E-02 |
| Family C | Hyper | 47 | C0751265 | Learning Disabilities | DisGeNET BeFree | 4.23E-05 | 1.47E-02 |
| Family C | Hyper | 48 | C1535926 | Neurodevelopmental Disorders | DisGeNET Curated | 6.28E-05 | 2.13E-02 |
| Family C | Hyper | 49 | C0014544 | Epilepsy | DisGeNET Curated | 6.60E-05 | 2.15E-02 |
| Family C | Hyper | 50 | C0345967 | Malignant mesothelioma | DisGeNET Curated | 6.60E-05 | 2.15E-02 |
| Family C | Hyper | 51 | C0004936 | Mental disorders | DisGeNET BeFree | 6.96E-05 | 2.22E-02 |
| Family C | Hyper | 52 | C0026837 | Muscle Rigidity | DisGeNET BeFree | 7.47E-05 | 2.34E-02 |
| Family C | Hyper | 53 | C0007222 | Cardiovascular Diseases | DisGeNET BeFree | 7.74E-05 | 2.38E-02 |
| Family C | Hyper | 54 | C0027819 | Neuroblastoma | DisGeNET BeFree | 7.96E-05 | 2.40E-02 |
| Family C | Hyper | 55 | C1611743 | Familial (FPAH) | DisGeNET BeFree | 8.56E-05 | 2.51E-02 |
| Family C | Hyper | 56 | C0020429 | Hyperalgesia | DisGeNET BeFree | 8.64E-05 | 2.51E-02 |
| Family C | Hyper | 57 | C0600520 | Left Ventricle Remodeling | DisGeNET Curated | 9.81E-05 | 2.76E-02 |
| Family C | Hyper | 58 | C0600519 | Ventricular Remodeling | DisGeNET Curated | 9.81E-05 | 2.76E-02 |
| Family C | Hyper | 59 | C0424296 | Social disinhibition | DisGeNET BeFree | 1.03E-04 | 2.81E-02 |
| Family C | Hyper | 60 | C0005586 | Bipolar Disorder | DisGeNET Curated | 1.04E-04 | 2.81E-02 |
| Family C | Hyper | 61 | C0700095 | Central neuroblastoma | DisGeNET BeFree | 1.06E-04 | 2.84E-02 |
| Family C | Hyper | 62 | C4086165 | Childhood Neuroblastoma | DisGeNET BeFree | 1.10E-04 | 2.89E-02 |
| Family C | Hyper | 63 | C0009241 | Cognition Disorders | DisGeNET BeFree | 1.19E-04 | 3.08E-02 |
| Family C | Hyper | 64 | C0014544 | Epilepsy | DisGeNET BeFree | 1.23E-04 | 3.12E-02 |
| Family C | Hyper | 65 | C0524620 | Metabolic Syndrome X | DisGeNET BeFree | 1.28E-04 | 3.13E-02 |
| Family C | Hyper | 66 | C0020538 | Hypertensive disease | DisGeNET BeFree | 1.28E-04 | 3.13E-02 |
| Family C | Hyper | 67 | C0221271 | Elastosis perforans serpiginosa | DisGeNET BeFree | 1.29E-04 | 3.13E-02 |
| Family C | Hyper | 68 | C1565489 | Renal Insufficiency | DisGeNET BeFree | 1.46E-04 | 3.49E-02 |
| Family C | Hyper | 69 | C0233794 | Memory impairment | DisGeNET BeFree | 1.54E-04 | 3.60E-02 |
| Family C | Hyper | 70 | C0027051 | Myocardial Infarction | DisGeNET BeFree | 1.55E-04 | 3.60E-02 |
| Family C | Hyper | 71 | C0001973 | Alcoholic Intoxication, Chronic | DisGeNET BeFree | 1.76E-04 | 4.03E-02 |
| Family C | Hyper | 72 | C0006012 | Borderline Personality Disorder | DisGeNET BeFree | 2.04E-04 | 4.61E-02 |
| Family C | Hyper | 73 | C0026650 | Movement Disorders | DisGeNET BeFree | 2.17E-04 | 4.85E-02 |
| Family C | Hypo | 1 | C2711227 | Steatohepatitis | DisGeNET BeFree | 3.96E-06 | 2.84E-02 |
| Family C | Hypo | 2 | C0334583 | Pilocytic Astrocytoma | DisGeNET BeFree | 4.68E-06 | 2.84E-02 |
| Family C | Hypo | 3 | C0027765 | nervous system disorder | DisGeNET BeFree | 8.90E-06 | 2.84E-02 |
| Family C | Hypo | 4 | C0023448 | Lymphoid leukemia | DisGeNET BeFree | 1.82E-05 | 2.84E-02 |
| Family C | Hypo | 5 | C0149931 | Migraine Disorders | DisGeNET BeFree | 1.98E-05 | 2.84E-02 |
| Family C | Hypo | 6 | C0013384 | Dyskinetic syndrome | DisGeNET BeFree | 2.03E-05 | 2.84E-02 |
| Family C | Hypo | 7 | C1332977 | Childhood Leukemia | DisGeNET BeFree | 2.15E-05 | 2.84E-02 |
| Family C | Hypo | 8 | C0740858 | Substance abuse problem | DisGeNET BeFree | 2.30E-05 | 2.84E-02 |
| Family C | Hypo | 9 | C0023418 | leukemia | DisGeNET BeFree | 3.37E-05 | 2.84E-02 |
| Family C | Hypo | 10 | C0271650 | Impaired glucose tolerance | DisGeNET BeFree | 4.57E-05 | 2.84E-02 |
| Family C | Hypo | 11 | C0338656 | Impaired cognition | DisGeNET BeFree | 4.91E-05 | 2.84E-02 |
| Family C | Hypo | 12 | C2267227 | Bulimia Nervosa | DisGeNET BeFree | 5.14E-05 | 2.84E-02 |
| Family C | Hypo | 13 | C3642347 | Basal-Like Breast Carcinoma | DisGeNET BeFree | 5.59E-05 | 2.84E-02 |
| Family C | Hypo | 14 | C0270824 | Visual seizure | DisGeNET Curated | 7.13E-05 | 2.84E-02 |
| Family C | Hypo | 15 | C0270846 | Epileptic drop attack | DisGeNET Curated | 7.13E-05 | 2.84E-02 |
| Family C | Hypo | 16 | C0234533 | Generalized seizures | DisGeNET Curated | 7.13E-05 | 2.84E-02 |
| Family C | Hypo | 17 | C0234535 | Clonic Seizures | DisGeNET Curated | 7.13E-05 | 2.84E-02 |
| Family C | Hypo | 18 | C0751056 | Non-epileptic convulsion | DisGeNET Curated | 7.13E-05 | 2.84E-02 |
| Family C | Hypo | 19 | C0751123 | Atonic Absence Seizures | DisGeNET Curated | 7.13E-05 | 2.84E-02 |
| Family C | Hypo | 20 | C0751110 | Single Seizure | DisGeNET Curated | 7.13E-05 | 2.84E-02 |
| Family C | Hypo | 21 | C0751494 | Convulsive Seizures | DisGeNET Curated | 7.13E-05 | 2.84E-02 |
| Family C | Hypo | 22 | C0751496 | Seizures, Sensory | DisGeNET Curated | 7.13E-05 | 2.84E-02 |
| Family C | Hypo | 23 | C0149958 | Complex partial seizures | DisGeNET Curated | 7.13E-05 | 2.84E-02 |
| Family C | Hypo | 24 | C3495874 | Nonepileptic Seizures | DisGeNET Curated | 7.13E-05 | 2.84E-02 |
| Family C | Hypo | 25 | C4505436 | Generalized Absence Seizures | DisGeNET Curated | 7.13E-05 | 2.84E-02 |
| Family C | Hypo | 26 | C0422855 | Vertiginous seizure | DisGeNET Curated | 7.13E-05 | 2.84E-02 |
| Family C | Hypo | 27 | C0422854 | Gustatory seizure | DisGeNET Curated | 7.13E-05 | 2.84E-02 |
| Family C | Hypo | 28 | C0422850 | Seizures, Somatosensory | DisGeNET Curated | 7.13E-05 | 2.84E-02 |
| Family C | Hypo | 29 | C0422853 | Olfactory seizure | DisGeNET Curated | 7.13E-05 | 2.84E-02 |
| Family C | Hypo | 30 | C0422852 | Seizures, Auditory | DisGeNET Curated | 7.13E-05 | 2.84E-02 |
| Family C | Hypo | 31 | C0022333 | Jacksonian Seizure | DisGeNET Curated | 7.13E-05 | 2.84E-02 |
| Family C | Hypo | 32 | C4317109 | Epileptic Seizures | DisGeNET Curated | 7.13E-05 | 2.84E-02 |
| Family C | Hypo | 33 | C0018801 | Heart failure | DisGeNET BeFree | 7.39E-05 | 2.85E-02 |
| Family C | Hypo | 34 | C4316903 | Absence Seizures | DisGeNET Curated | 8.24E-05 | 2.92E-02 |
| Family C | Hypo | 35 | C4048158 | Convulsions | DisGeNET Curated | 8.24E-05 | 2.92E-02 |
| Family C | Hypo | 36 | C0270844 | Tonic Seizures | DisGeNET Curated | 8.24E-05 | 2.92E-02 |
| Family C | Hypo | 37 | C0085207 | Gestational Diabetes | DisGeNET BeFree | 8.62E-05 | 2.97E-02 |
| Family C | Hypo | 38 | C0015695 | Fatty Liver | DisGeNET BeFree | 9.11E-05 | 3.06E-02 |
| Family C | Hypo | 39 | C0279583 | Childhood T Acute Lymphoblastic Leukemia | DisGeNET BeFree | 9.37E-05 | 3.06E-02 |
| Family C | Hypo | 40 | C0005586 | Bipolar Disorder | DisGeNET Curated | 1.01E-04 | 3.16E-02 |
| Family C | Hypo | 41 | C0279565 | Invasive Lobular Breast Carcinoma | DisGeNET BeFree | 1.04E-04 | 3.16E-02 |
| Family C | Hypo | 42 | C0751495 | Seizures, Focal | DisGeNET Curated | 1.09E-04 | 3.16E-02 |
| Family C | Hypo | 43 | C0494475 | Tonic - clonic seizures | DisGeNET Curated | 1.09E-04 | 3.16E-02 |
| Family C | Hypo | 44 | C4317123 | Myoclonic Seizures | DisGeNET Curated | 1.09E-04 | 3.16E-02 |
| Family C | Hypo | 45 | C0265509 | Congenital anomaly of skeletal bone | DisGeNET BeFree | 1.21E-04 | 3.20E-02 |
| Family C | Hypo | 46 | C0853892 | Catabolic state | DisGeNET BeFree | 1.21E-04 | 3.20E-02 |
| Family C | Hypo | 47 | C0677886 | Epithelial ovarian cancer | DisGeNET BeFree | 1.22E-04 | 3.20E-02 |
| Family C | Hypo | 48 | C0038443 | Stress, Psychological | DisGeNET BeFree | 1.22E-04 | 3.20E-02 |
| Family C | Hypo | 49 | C0206658 | Smooth Muscle Tumor | DisGeNET BeFree | 1.23E-04 | 3.20E-02 |
| Family C | Hypo | 50 | C4288891 | Infant T Acute Lymphoblastic Leukemia | DisGeNET BeFree | 1.48E-04 | 3.78E-02 |
| Family C | Hypo | 51 | C0005699 | Blast Phase | DisGeNET BeFree | 1.53E-04 | 3.81E-02 |
| Family C | Hypo | 52 | C0019569 | Hirschsprung Disease | DisGeNET BeFree | 1.66E-04 | 4.00E-02 |
| Family C | Hypo | 53 | C0007134 | Renal Cell Carcinoma | DisGeNET BeFree | 1.67E-04 | 4.00E-02 |
| Family C | Hypo | 54 | C0011581 | Depressive disorder | DisGeNET BeFree | 1.84E-04 | 4.35E-02 |
| Family C | Hypo | 55 | C0030567 | Parkinson Disease | DisGeNET BeFree | 1.95E-04 | 4.51E-02 |
| Family C | Hypo | 56 | C0024301 | Lymphoma, Follicular | DisGeNET BeFree | 2.09E-04 | 4.71E-02 |
| Family C | Hypo | 57 | C0035344 | Retinopathy of Prematurity | DisGeNET BeFree | 2.14E-04 | 4.71E-02 |
| Family C | Hypo | 58 | C2062441 | Influenza A | DisGeNET BeFree | 2.14E-04 | 4.71E-02 |
| Family C | Hypo | 59 | C0001973 | Alcoholic Intoxication, Chronic | DisGeNET BeFree | 2.31E-04 | 4.92E-02 |
| Family C | Hypo | 60 | C0018802 | Congestive heart failure | DisGeNET BeFree | 2.32E-04 | 4.92E-02 |

(*) IDs are unique to the associated database. (†) P-values were calculated using the hypergeometric test. (‡) FDR B&H: False discovery rates were calculated by the Benjamini and Hochberg method^3^.

**Abbreviations:** DMR, differentially methylated region; Hyper, hypermethylated; Hypo, hypomethylated.

**Table S8**

**OR statistics for iPSC DMR and histone modifications**

| **DMR Group** | **DMR Type** | **Histone Mark** | **a*** | **b*** | **c*** | **d*** | **OR†** | **log(OR)** | **p-value‡** |
| --- | --- | --- | --- | --- | --- | --- | --- | --- | --- |
| Shared | Hyper | H3K27Ac | 326 | 1015793 | 1126 | 3408263 | 9.71E-01 | -1.26E-02 | 6.62E-01 |
| Shared | Hyper | H3K27me3 | 532 | 1238261 | 920 | 3185589 | 1.49E+00 | 1.73E-01 | 1.02E-12 |
| Shared | Hyper | H3K36me3 | 366 | 1355818 | 1086 | 3068198 | 7.63E-01 | -1.18E-01 | 5.12E-06 |
| Shared | Hyper | H3K4me1 | 445 | 899637 | 1007 | 3524300 | 1.73E+00 | 2.38E-01 | 2.08E-20 |
| Shared | Hyper | H3K4me3 | 328 | 951104 | 1124 | 3472950 | 1.07E+00 | 2.76E-02 | 3.07E-01 |
| Shared | Hyper | H3K9me3 | 308 | 747719 | 1144 | 3676355 | 1.32E+00 | 1.22E-01 | 2.19E-05 |
| Shared | Hypo | H3K27Ac | 116 | 1016003 | 890 | 3408709 | 4.37E-01 | -3.59E-01 | 1.95E-20 |
| Shared | Hypo | H3K27me3 | 361 | 1238432 | 645 | 3186035 | 1.44E+00 | 1.58E-01 | 5.85E-08 |
| Shared | Hypo | H3K36me3 | 235 | 1355949 | 771 | 3068644 | 6.90E-01 | -1.61E-01 | 3.27E-07 |
| Shared | Hypo | H3K4me1 | 169 | 899913 | 837 | 3524746 | 7.91E-01 | -1.02E-01 | 4.78E-03 |
| Shared | Hypo | H3K4me3 | 160 | 951272 | 846 | 3473396 | 6.91E-01 | -1.61E-01 | 9.73E-06 |
| Shared | Hypo | H3K9me3 | 249 | 747778 | 757 | 3676801 | 1.62E+00 | 2.09E-01 | 2.96E-10 |
| Family A | Hyper | H3K27Ac | 1946 | 912786 | 9406 | 3009559 | 6.82E-01 | -1.66E-01 | 2.13E-57 |
| Family A | Hyper | H3K27me3 | 3613 | 1099621 | 7739 | 2821057 | 1.20E+00 | 7.84E-02 | 1.01E-18 |
| Family A | Hyper | H3K36me3 | 2645 | 1211411 | 8707 | 2710235 | 6.80E-01 | -1.68E-01 | 6.64E-72 |
| Family A | Hyper | H3K4me1 | 2502 | 801355 | 8850 | 3120434 | 1.10E+00 | 4.17E-02 | 2.71E-05 |
| Family A | Hyper | H3K4me3 | 1931 | 860760 | 9421 | 3061600 | 7.29E-01 | -1.37E-01 | 5.58E-39 |
| Family A | Hyper | H3K9me3 | 2626 | 661875 | 8726 | 3259790 | 1.48E+00 | 1.71E-01 | 6.65E-65 |
| Family A | Hypo | H3K27Ac | 1540 | 913192 | 5963 | 3013408 | 8.52E-01 | -6.94E-02 | 1.47E-08 |
| Family A | Hypo | H3K27me3 | 2781 | 1100453 | 4722 | 2824906 | 1.51E+00 | 1.80E-01 | 3.08E-64 |
| Family A | Hypo | H3K36me3 | 1871 | 1212185 | 5632 | 2714084 | 7.44E-01 | -1.29E-01 | 8.57E-30 |
| Family A | Hypo | H3K4me1 | 1896 | 801961 | 5607 | 3124283 | 1.32E+00 | 1.20E-01 | 4.61E-24 |
| Family A | Hypo | H3K4me3 | 1654 | 861037 | 5849 | 3065449 | 1.01E+00 | 2.93E-03 | 8.12E-01 |
| Family A | Hypo | H3K9me3 | 1475 | 663026 | 6028 | 3263639 | 1.20E+00 | 8.08E-02 | 3.37E-10 |
| Family C | Hyper | H3K27Ac | 1405 | 805630 | 7602 | 2623702 | 6.02E-01 | -2.20E-01 | 4.48E-76 |
| Family C | Hyper | H3K27me3 | 3151 | 963853 | 5856 | 2463733 | 1.38E+00 | 1.38E-01 | 1.75E-45 |
| Family C | Hyper | H3K36me3 | 2013 | 1058107 | 6994 | 2370617 | 6.45E-01 | -1.91E-01 | 2.21E-72 |
| Family C | Hyper | H3K4me1 | 1701 | 707970 | 7306 | 2721066 | 8.95E-01 | -4.83E-02 | 3.20E-05 |
| Family C | Hyper | H3K4me3 | 1362 | 763549 | 7645 | 2665826 | 6.22E-01 | -2.06E-01 | 7.55E-65 |
| Family C | Hyper | H3K9me3 | 1696 | 574902 | 7311 | 2854139 | 1.15E+00 | 6.13E-02 | 2.53E-07 |
| Family C | Hypo | H3K27Ac | 1231 | 805804 | 5425 | 2626053 | 7.39E-01 | -1.31E-01 | 8.45E-23 |
| Family C | Hypo | H3K27me3 | 2421 | 964583 | 4235 | 2466084 | 1.46E+00 | 1.65E-01 | 3.34E-48 |
| Family C | Hypo | H3K36me3 | 1586 | 1058534 | 5070 | 2372968 | 7.01E-01 | -1.54E-01 | 8.19E-37 |
| Family C | Hypo | H3K4me1 | 1391 | 708280 | 5265 | 2723417 | 1.02E+00 | 6.84E-03 | 6.06E-01 |
| Family C | Hypo | H3K4me3 | 1352 | 763559 | 5304 | 2668177 | 8.91E-01 | -5.03E-02 | 1.33E-04 |
| Family C | Hypo | H3K9me3 | 1249 | 575349 | 5407 | 2856490 | 1.15E+00 | 5.95E-02 | 1.68E-05 |

(*) *a*, *b*, *c*, and *d* values are the contingency parameters used to calculate OR. (†) OR was calculated as described in the Methods section. (‡) P-values were calculated by Fisher’s exact test.

**Abbreviations:** OR, odds ratio; DMR, differentially methylated region; iPSC, induced pluripotent stem cell.

**Table S9**

**Complete list of disease ontology terms from ToppGene for gene lists associated with fibroblast and iPSC DMRs**

| **DMR group** | **DMR type**  **(Fibroblast to iPSC)** | **Rank** | **ID*** | **Name** | **Source** | **p-value**† | **FDR B&H**‡ |
| --- | --- | --- | --- | --- | --- | --- | --- |
| Family C | Hyper to Hypo | 1 | C0014544 | Epilepsy | DisGeNET BeFree | 2.22E-07 | 9.66E-04 |
| Family C | Hyper to Hypo | 2 | C0424605 | Developmental delay (disorder) | DisGeNET BeFree | 2.75E-07 | 9.66E-04 |
| Family C | Hyper to Hypo | 3 | C0000768 | Congenital Abnormality | DisGeNET BeFree | 4.71E-07 | 1.10E-03 |
| Family C | Hyper to Hypo | 4 | C0008073 | Developmental Disabilities | DisGeNET BeFree | 1.23E-06 | 2.17E-03 |
| Family C | Hyper to Hypo | 5 | C0557874 | Global developmental delay | DisGeNET BeFree | 3.22E-06 | 4.53E-03 |
| Family C | Hyper to Hypo | 6 | C0221357 | Brachydactyly | DisGeNET BeFree | 4.3E-06 | 5.04E-03 |
| Family C | Hyper to Hypo | 7 | C0023418 | leukemia | DisGeNET BeFree | 7.58E-06 | 7.61E-03 |
| Family C | Hyper to Hypo | 8 | C0598766 | Leukemogenesis | DisGeNET BeFree | 2.29E-05 | 1.89E-02 |
| Family C | Hyper to Hypo | 9 | C3714756 | Intellectual Disability | DisGeNET BeFree | 2.47E-05 | 1.89E-02 |
| Family C | Hyper to Hypo | 10 | C4021790 | Abnormality of the skeletal system | DisGeNET BeFree | 2.69E-05 | 1.89E-02 |
| Family C | Hyper to Hypo | 11 | C0524528 | Pervasive Development Disorder | DisGeNET BeFree | 3.7E-05 | 2.25E-02 |
| Family C | Hyper to Hypo | 12 | C0036572 | Seizures | DisGeNET BeFree | 4.13E-05 | 2.25E-02 |
| Family C | Hyper to Hypo | 13 | C1332977 | Childhood Leukemia | DisGeNET BeFree | 4.15E-05 | 2.25E-02 |
| Family C | Hyper to Hypo | 14 | C0221356 | Brachycephaly | DisGeNET BeFree | 5.11E-05 | 2.25E-02 |
| Family C | Hyper to Hypo | 15 | C0410179 | Ullrich congenital muscular dystrophy 1 | DisGeNET Curated | 5.11E-05 | 2.25E-02 |
| Family C | Hyper to Hypo | 16 | C1834674 | BETHLEM MYOPATHY 1 | DisGeNET Curated | 5.11E-05 | 2.25E-02 |
| Family C | Hyper to Hypo | 17 | C0240340 | Microdontia (disorder) | DisGeNET BeFree | 8.81E-05 | 3.65E-02 |
| Family C | Hyper to Hypo | 18 | C0079218 | Fibromatosis, Aggressive | DisGeNET BeFree | 9.9E-05 | 3.87E-02 |
| Family C | Hyper to Hypo | 19 | C0008029 | Cherubism | DisGeNET BeFree | 0.000106 | 3.92E-02 |
| Family C | Hyper to Hypo | 20 | C0025958 | Microcephaly | DisGeNET BeFree | 0.000129 | 4.53E-02 |
| Family C | Hyper to Hypo | 21 | C0265354 | CHARGE Syndrome | DisGeNET BeFree | 0.000141 | 4.72E-02 |
| Family C | Hyper to Hyper | 1 | C0000768 | Congenital Abnormality | DisGeNET BeFree | 8.69E-09 | 5.12E-05 |
| Family C | Hyper to Hyper | 2 | C0000846 | Agenesis | DisGeNET BeFree | 2.94E-06 | 8.68E-03 |
| Family C | Hypo to Hyper | 1 | C0000768 | Congenital Abnormality | DisGeNET BeFree | 1.02E-05 | 3.06E-02 |
| Family C | Hypo to Hyper | 2 | C0424605 | Developmental delay (disorder) | DisGeNET BeFree | 1.28E-05 | 3.06E-02 |
| Family C | Hypo to Hyper | 3 | C0013080 | Down Syndrome | DisGeNET BeFree | 2.67E-05 | 4.26E-02 |
| Family C | Hypo to Hyper | 4 | C1449563 | Cardiomyopathy, Familial Idiopathic | DisGeNET BeFree | 4.51E-05 | 4.77E-02 |
| Family C | Hypo to Hyper | 5 | C0003873 | Rheumatoid Arthritis | DisGeNET Curated | 5.67E-05 | 4.77E-02 |
| Family C | Hypo to Hyper | 6 | C0557874 | Global developmental delay | DisGeNET BeFree | 5.98E-05 | 4.77E-02 |
| Family C | Non-DMR to Hyper | none | none | none | none | none | none |
| Family C | Hypo to Hypo | 1 | C0014544 | Epilepsy | DisGeNET BeFree | 3.66E-07 | 2.71E-03 |
| Family C | Hypo to Hypo | 2 | C0008925 | Cleft Palate | DisGeNET BeFree | 1.69E-05 | 1.68E-02 |
| Family C | Hypo to Hypo | 3 | C1535926 | Neurodevelopmental Disorders | DisGeNET BeFree | 2.64E-05 | 1.68E-02 |
| Family C | Hypo to Hypo | 4 | C0000768 | Congenital Abnormality | DisGeNET BeFree | 2.97E-05 | 1.68E-02 |
| Family C | Hypo to Hypo | 5 | C0270824 | Visual seizure | DisGeNET Curated | 5.9E-05 | 1.68E-02 |
| Family C | Hypo to Hypo | 6 | C0270846 | Epileptic drop attack | DisGeNET Curated | 5.9E-05 | 1.68E-02 |
| Family C | Hypo to Hypo | 7 | C0234533 | Generalized seizures | DisGeNET Curated | 5.9E-05 | 1.68E-02 |
| Family C | Hypo to Hypo | 8 | C0234535 | Clonic Seizures | DisGeNET Curated | 5.9E-05 | 1.68E-02 |
| Family C | Hypo to Hypo | 9 | C0751056 | Non-epileptic convulsion | DisGeNET Curated | 5.9E-05 | 1.68E-02 |
| Family C | Hypo to Hypo | 10 | C0751123 | Atonic Absence Seizures | DisGeNET Curated | 5.9E-05 | 1.68E-02 |
| Family C | Hypo to Hypo | 11 | C0751110 | Single Seizure | DisGeNET Curated | 5.9E-05 | 1.68E-02 |
| Family C | Hypo to Hypo | 12 | C0422855 | Vertiginous seizure | DisGeNET Curated | 5.9E-05 | 1.68E-02 |
| Family C | Hypo to Hypo | 13 | C0422854 | Gustatory seizure | DisGeNET Curated | 5.9E-05 | 1.68E-02 |
| Family C | Hypo to Hypo | 14 | C0422850 | Seizures, Somatosensory | DisGeNET Curated | 5.9E-05 | 1.68E-02 |
| Family C | Hypo to Hypo | 15 | C0422853 | Olfactory seizure | DisGeNET Curated | 5.9E-05 | 1.68E-02 |
| Family C | Hypo to Hypo | 16 | C0422852 | Seizures, Auditory | DisGeNET Curated | 5.9E-05 | 1.68E-02 |
| Family C | Hypo to Hypo | 17 | C0751494 | Convulsive Seizures | DisGeNET Curated | 5.9E-05 | 1.68E-02 |
| Family C | Hypo to Hypo | 18 | C0751496 | Seizures, Sensory | DisGeNET Curated | 5.9E-05 | 1.68E-02 |
| Family C | Hypo to Hypo | 19 | C0149958 | Complex partial seizures | DisGeNET Curated | 5.9E-05 | 1.68E-02 |
| Family C | Hypo to Hypo | 20 | C3495874 | Nonepileptic Seizures | DisGeNET Curated | 5.9E-05 | 1.68E-02 |
| Family C | Hypo to Hypo | 21 | C4505436 | Generalized Absence Seizures | DisGeNET Curated | 5.9E-05 | 1.68E-02 |
| Family C | Hypo to Hypo | 22 | C0022333 | Jacksonian Seizure | DisGeNET Curated | 5.9E-05 | 1.68E-02 |
| Family C | Hypo to Hypo | 23 | C4317109 | Epileptic Seizures | DisGeNET Curated | 5.9E-05 | 1.68E-02 |
| Family C | Hypo to Hypo | 24 | C4707243 | Familial thoracic aortic aneurysm and aortic dissection | DisGeNET Curated | 5.91E-05 | 1.68E-02 |
| Family C | Hypo to Hypo | 25 | C4316903 | Absence Seizures | DisGeNET Curated | 6.46E-05 | 1.68E-02 |
| Family C | Non-DMR to Hypo | none | none | none | none | none | none |
| Family A | Hyper to Hypo | 1 | C0270764 | Motor Neuron Disease, Lower | DisGeNET BeFree | 2.29E-06 | 4.70E-03 |
| Family A | Hyper to Hypo | 2 | C0524730 | Odontome | DisGeNET Curated | 3.07E-06 | 4.70E-03 |
| Family A | Hyper to Hypo | 3 | C0040427 | Tooth Abnormalities | DisGeNET Curated | 3.07E-06 | 4.70E-03 |
| Family A | Hyper to Hypo | 4 | C0206762 | Limb Deformities, Congenital | DisGeNET BeFree | 1.05E-05 | 1.21E-02 |
| Family A | Hyper to Hypo | 5 | C1839839 | MAJOR AFFECTIVE DISORDER 2 | DisGeNET Curated | 3.27E-05 | 3.00E-02 |
| Family A | Hyper to Hypo | 6 | C0850639 | premalignant lesion | DisGeNET BeFree | 6.48E-05 | 4.96E-02 |
| Family A | Hyper to Hyper | none | none | none | none | none | none |
| Family A | Hypo to Hyper | none | none | none | none | none | none |
| Family A | Non-DMR to Hyper | 1 | C0917796 | Optic Atrophy, Hereditary, Leber | DisGeNET Curated | 5.01E-06 | 2.36E-02 |
| Family A | Non-DMR to Hyper | 2 | 535000 | LEBER OPTIC ATROPHY | OMIM MedGen | 1.26E-05 | 2.36E-02 |
| Family A | Non-DMR to Hyper | 3 | cv:C0917796 | Leber's optic atrophy | Clinical Variations | 1.26E-05 | 2.36E-02 |
| Family A | Hypo to Hypo | 1 | C0266544 | Microcornea | DisGeNET BeFree | 4.34E-06 | 1.76E-02 |
| Family A | Hypo to Hypo | 2 | C1839839 | MAJOR AFFECTIVE DISORDER 2 | DisGeNET Curated | 1.4E-05 | 2.85E-02 |
| Family A | Hypo to Hypo | 3 | C0007124 | Noninfiltrating Intraductal Carcinoma | DisGeNET BeFree | 2.53E-05 | 3.43E-02 |
| Family A | Non-DMR to Hypo | none | none | none | none | none | none |

(*) IDs are unique to the associated database. (†) P-values were calculated using the hypergeometric test. (‡) FDR B&H: False discovery rates were calculated by the Benjamini and Hochberg method^3^.

**Abbreviations:** DMR, differentially methylated region; Hyper, hypermethylated; Hypo, hypomethylation; iPSC, induced pluripotent stem cell.

**Table S10**

**KEGG pathway enrichment for the set of 28 genes associated to DMRs whose methylation change is hypermethylated in fibroblast and hypomethylated in iPSC, acquired from STRING**

| **#term ID** | **term description** | **Strength*** | **FDR†** | **matching proteins in the network (labels)‡** |
| --- | --- | --- | --- | --- |
| hsa04340 | Hedgehog signaling pathway | 1.66 | 4.10E-04 | CCND1,SHH,PTCH1 |
| hsa04933 | AGE-RAGE signaling pathway in diabetic complications | 1.63 | 1.01E-06 | CCND1,RAC1,CDC42,IL6,NFATC1,PRKCA |
| hsa05143 | African trypanosomiasis | 1.61 | 4.90E-03 | IL6,PRKCA |
| hsa05130 | Pathogenic Escherichia coli infection | 1.6 | 5.50E-04 | ROCK1,CDC42,PRKCA |
| hsa04370 | VEGF signaling pathway | 1.55 | 7.10E-04 | RAC1,CDC42,PRKCA |
| hsa05132 | Salmonella infection | 1.52 | 1.10E-04 | RAC1,ROCK1,CDC42,IL6 |
| hsa05131 | Shigellosis | 1.52 | 7.80E-04 | RAC1,ROCK1,CDC42 |
| hsa04666 | Fc gamma R-mediated phagocytosis | 1.5 | 1.20E-04 | RAC1,SYK,CDC42,PRKCA |
| hsa04664 | Fc epsilon RI signaling pathway | 1.5 | 8.50E-04 | RAC1,SYK,PRKCA |
| hsa05211 | Renal cell carcinoma | 1.49 | 8.50E-04 | CREBBP,RAC1,CDC42 |
| hsa04520 | Adherens junction | 1.47 | 9.30E-04 | CREBBP,RAC1,CDC42 |
| hsa04662 | B cell receptor signaling pathway | 1.47 | 9.30E-04 | RAC1,SYK,NFATC1 |
| hsa05212 | Pancreatic cancer | 1.45 | 9.70E-04 | CCND1,RAC1,CDC42 |
| hsa05205 | Proteoglycans in cancer | 1.4 | 1.01E-06 | CCND1,SHH,PTCH1,RAC1,  ROCK1,CDC42,PRKCA |
| hsa04670 | Leukocyte transendothelial migration | 1.4 | 2.30E-04 | RAC1,ROCK1,CDC42,PRKCA |
| hsa05416 | Viral myocarditis | 1.4 | 1.02E-02 | CCND1,RAC1 |
| hsa04310 | Wnt signaling pathway | 1.39 | 4.12E-05 | CCND1,CREBBP,RAC1,NFATC1,PRKCA |
| hsa05161 | Hepatitis B | 1.39 | 4.12E-05 | CCND1,CREBBP,IL6,NFATC1,PRKCA |
| hsa04360 | Axon guidance | 1.38 | 6.48E-06 | SHH,PTCH1,RAC1,ROCK1,CDC42,PRKCA |
| hsa05206 | MicroRNAs in cancer | 1.37 | 4.12E-05 | CCND1,CREBBP,HDAC4,ROCK1,PRKCA |
| hsa00310 | Lysine degradation | 1.37 | 1.06E-02 | KMT2C,EHMT1 |
| hsa05167 | Kaposi's sarcoma-associated herpesvirus infection | 1.36 | 7.14E-06 | CCND1,CREBBP,RAC1,SYK,IL6,NFATC1 |
| hsa05203 | Viral carcinogenesis | 1.36 | 7.14E-06 | CCND1,CREBBP,HDAC4,RAC1,SYK,CDC42 |
| hsa04650 | Natural killer cell mediated cytotoxicity | 1.35 | 3.00E-04 | RAC1,SYK,NFATC1,PRKCA |
| hsa05321 | Inflammatory bowel disease (IBD) | 1.35 | 1.14E-02 | IL6,NFATC1 |
| hsa05217 | Basal cell carcinoma | 1.35 | 1.15E-02 | SHH,PTCH1 |
| hsa04720 | Long-term potentiation | 1.34 | 1.17E-02 | CREBBP,PRKCA |
| hsa04066 | HIF-1 signaling pathway | 1.33 | 2.10E-03 | CREBBP,IL6,PRKCA |
| hsa05120 | Epithelial cell signaling in Helicobacter pylori infection | 1.33 | 1.21E-02 | RAC1,CDC42 |
| hsa05223 | Non-small cell lung cancer | 1.33 | 1.21E-02 | CCND1,PRKCA |
| hsa05214 | Glioma | 1.31 | 1.22E-02 | CCND1,PRKCA |
| hsa05100 | Bacterial invasion of epithelial cells | 1.29 | 1.33E-02 | RAC1,CDC42 |
| hsa04921 | Oxytocin signaling pathway | 1.27 | 5.30E-04 | CCND1,ROCK1,NFATC1,PRKCA |
| hsa04071 | Sphingolipid signaling pathway | 1.26 | 3.20E-03 | RAC1,ROCK1,PRKCA |
| hsa04919 | Thyroid hormone signaling pathway | 1.26 | 3.20E-03 | CCND1,CREBBP,PRKCA |
| hsa04024 | cAMP signaling pathway | 1.25 | 1.20E-04 | CREBBP,PTCH1,RAC1,ROCK1,NFATC1 |
| hsa04510 | Focal adhesion | 1.25 | 1.20E-04 | CCND1,RAC1,ROCK1,CDC42,PRKCA |
| hsa01521 | EGFR tyrosine kinase inhibitor resistance | 1.25 | 1.52E-02 | IL6,PRKCA |
| hsa04110 | Cell cycle | 1.23 | 3.60E-03 | CCND1,CREBBP,CDKN1C |
| hsa04380 | Osteoclast differentiation | 1.23 | 3.60E-03 | RAC1,SYK,NFATC1 |
| hsa04350 | TGF-beta signaling pathway | 1.23 | 1.68E-02 | CREBBP,ROCK1 |
| hsa04530 | Tight junction | 1.22 | 7.10E-04 | CCND1,RAC1,ROCK1,CDC42 |
| hsa05210 | Colorectal cancer | 1.22 | 1.73E-02 | CCND1,RAC1 |
| hsa04068 | FoxO signaling pathway | 1.21 | 3.80E-03 | CCND1,CREBBP,IL6 |
| hsa04912 | GnRH signaling pathway | 1.2 | 1.82E-02 | CDC42,PRKCA |
| hsa04972 | Pancreatic secretion | 1.17 | 2.03E-02 | RAC1,PRKCA |
| hsa05146 | Amoebiasis | 1.17 | 2.03E-02 | IL6,PRKCA |
| hsa05215 | Prostate cancer | 1.16 | 2.08E-02 | CCND1,CREBBP |
| hsa05166 | HTLV-I infection | 1.15 | 2.80E-04 | CCND1,CREBBP,ATF3,IL6,NFATC1 |
| hsa04932 | Non-alcoholic fatty liver disease (NAFLD) | 1.15 | 5.20E-03 | RAC1,CDC42,IL6 |
| hsa04660 | T cell receptor signaling pathway | 1.15 | 2.09E-02 | CDC42,NFATC1 |
| hsa04916 | Melanogenesis | 1.15 | 2.09E-02 | CREBBP,PRKCA |
| hsa05231 | Choline metabolism in cancer | 1.15 | 2.09E-02 | RAC1,PRKCA |
| hsa04620 | Toll-like receptor signaling pathway | 1.14 | 2.15E-02 | RAC1,IL6 |
| hsa04659 | Th17 cell differentiation | 1.14 | 2.15E-02 | IL6,NFATC1 |
| hsa04218 | Cellular senescence | 1.13 | 5.80E-03 | CCND1,IL6,NFATC1 |
| hsa04630 | Jak-STAT signaling pathway | 1.12 | 6.00E-03 | CCND1,CREBBP,IL6 |
| hsa05225 | Hepatocellular carcinoma | 1.11 | 6.20E-03 | CCND1,ARID1B,PRKCA |
| hsa05164 | Influenza A | 1.1 | 6.60E-03 | CREBBP,IL6,PRKCA |
| hsa05200 | Pathways in cancer | 1.09 | 1.45E-06 | CCND1,CREBBP,SHH,PTCH1,RAC1,  ROCK1,CDC42,IL6,PRKCA |
| hsa05152 | Tuberculosis | 1.09 | 6.90E-03 | CREBBP,SYK,IL6 |
| hsa04722 | Neurotrophin signaling pathway | 1.08 | 2.66E-02 | RAC1,CDC42 |
| hsa04270 | Vascular smooth muscle contraction | 1.07 | 2.76E-02 | ROCK1,PRKCA |
| hsa04062 | Chemokine signaling pathway | 1.06 | 7.80E-03 | RAC1,ROCK1,CDC42 |
| hsa04611 | Platelet activation | 1.06 | 2.89E-02 | SYK,ROCK1 |
| hsa05169 | Epstein-Barr virus infection | 1.03 | 9.20E-03 | CREBBP,HDAC4,SYK |
| hsa04210 | Apoptosis | 1.02 | 3.30E-02 | LMNB1,SPTAN1 |
| hsa04371 | Apelin signaling pathway | 1.02 | 3.30E-02 | CCND1,HDAC4 |
| hsa05162 | Measles | 1.02 | 3.30E-02 | CCND1,IL6 |
| hsa04015 | Rap1 signaling pathway | 1.01 | 1.02E-02 | RAC1,CDC42,PRKCA |
| hsa04810 | Regulation of actin cytoskeleton | 1.01 | 1.02E-02 | RAC1,ROCK1,CDC42 |
| hsa04151 | PI3K-Akt signaling pathway | 1 | 8.50E-04 | CCND1,RAC1,SYK,IL6,PRKCA |
| hsa04010 | MAPK signaling pathway | 0.98 | 3.60E-03 | RAC1,CDC42,NFATC1,PRKCA |
| hsa04072 | Phospholipase D signaling pathway | 0.98 | 3.72E-02 | SYK,PRKCA |
| hsa05226 | Gastric cancer | 0.98 | 3.77E-02 | CCND1,SHH |
| hsa04014 | Ras signaling pathway | 0.96 | 1.21E-02 | RAC1,CDC42,PRKCA |
| hsa05165 | Human papillomavirus infection | 0.95 | 4.50E-03 | CCND1,CREBBP,HDAC4,CDC42 |
| hsa04022 | cGMP-PKG signaling pathway | 0.94 | 4.35E-02 | ROCK1,NFATC1 |
| hsa05202 | Transcriptional misregulation in cancer | 0.92 | 4.75E-02 | MEIS1,IL6 |

(*) Strength is calculated by STRING as log_10_(observed/expected). (†) False discovery rates (FDR) were calculated by the Benjamini and Hochberg method^3^ as part of STRING. (‡) Specific genes related to each pathway.

**Table S11**

**Fibroblast and iPSC line pairs with corresponding genotype, sex, and age when skin biopsies were performed (N = 10)**

| **Cell ID** | **Family** | **Genotype*** | **Sex** | **Age at Skin Biopsy (years)†** |
| --- | --- | --- | --- | --- |
| P1 | A | +/- | F | 38 |
| P2 | A | +/- | M | 62 |
| P3 | A | +/- | F | 70 |
| P4 | C | +/- | M | 51 |
| P5 | C | +/- | M | 29 |
| C1 | A | +/+ | F | 49 |
| C2 | Donor | +/+ | M | 51 |
| C3 | A | +/+ | F | 68 |
| C4 | C | +/+ | F | 60 |
| C5 | C | +/+ | M | 26 |

(*) Genotype: +/+ homozygous normal, +/- heterozygous *LMNA* mutation. (†) Age: average age ± SD of Control (50.8 ± 16) vs. Patient (50 ± 17) is not significantly different p> 0.05, (t-test).

**Abbreviations:** iPSC, induced pluripotent stem cell

**Table S12**

**Table of antibodies used for pluripotency characterization of iPSCs**

| **Primary antibodies** | | | **Secondary antibodies** | | |
| --- | --- | --- | --- | --- | --- |
| **Antigen (host)** | **Company, Catalog no.** | **Dilution** | **Antigen (host)** | **Company, Catalog no.** | **Dilution** |
| OCT4 (rabbit) | TFS, A24867* | 1:100 | Anti-rabbit (donkey) AF-594 | TFS, A24870* | 1:250 |
| OCT4 (rabbit) | Abcam, ab181557 | 1:500 | Anti-rabbit (donkey) AF-594 | TFS, A24870* | 1:250 |
| SSEA4 (mouse) | TFS, A24866* | 1:100 | Anti-mouse (goat) AF-488 | TFS, A24877* | 1:250 |
| SSEA4 (mouse) | TFS, 414000 | 1:500 | Anti-mouse (goat) AF-488 | TFS, A24877* | 1:250 |
| SOX2 (rat) | TFS, A24759* | 1:100 | Anti-rat (donkey) AF-488 | TFS, A24876* | 1:250 |
| TRA-1-60 (mouse) | TFS, A24868* | 1:100 | Anti-mouse (goat) AF-594 | TFS, A24872* | 1:250 |
| TRA-1-60 (mouse) | TFS, MAB4360 | 1:500 | Anti-mouse (goat) AF-594 | TFS, A24872* | 1:250 |

(*) Pluripotent Stem Cell 4-Marker Immunocytochemistry Kit (A24881, Life Technologies).

**Abbreviations:** TFS, Thermo Fisher Scientific; AF, Alexa Fluor

**Table S13**

1. Hyper and hypomethylated DMR statistics in fibroblasts for all samples and by family

|  | **All Samples** | **Family A** | **Family C** |
| --- | --- | --- | --- |
| **Total DMR** | 1485 | 5713 | 4924 |
| **Ambiguous*** | 5 | 46 | 25 |
| **Unambiguous†** | 1480 | 5667 | 4899 |
| **Overlap filter‡** | 1479 | 5378 | 4725 |
| **Hyper DMR** | 885 | 3339 | 2872 |
| **Hypo DMR** | 594 | 2039 | 1853 |

1. Hyper and hypomethylated DMR statistics in iPSCs for all samples and by family

|  | **All Samples** | **Family A** | **Family C** |
| --- | --- | --- | --- |
| **Total DMR** | 511 | 1083 | 1547 |
| **Ambiguous*** | 1 | 8 | 2 |
| **Unambiguous†** | 510 | 1075 | 1545 |
| **Overlap filter‡** | 506 | 1004 | 1496 |
| **Hyper DMR** | 238 | 646 | 559 |
| **Hypo DMR** | 268 | 358 | 937 |

(*) Tiles containing differentially methylated CpGs with methylation differences with opposite directionality (hyper- or hypomethylation) were considered ambiguous. (†) Tiles containing differentially methylated CpGs with methylation differences with the same directionality. (‡) DMRs were filtered to keep only those with CpG methylation data found in both Family A and Family C.

**Abbreviations:** DMR, differentially methylated region; Hyper, hypermethylated; Hypo, hypomethylated; iPSC, induced pluripotent stem cell

1. **SUPPLEMENTARY FIGURES**

**
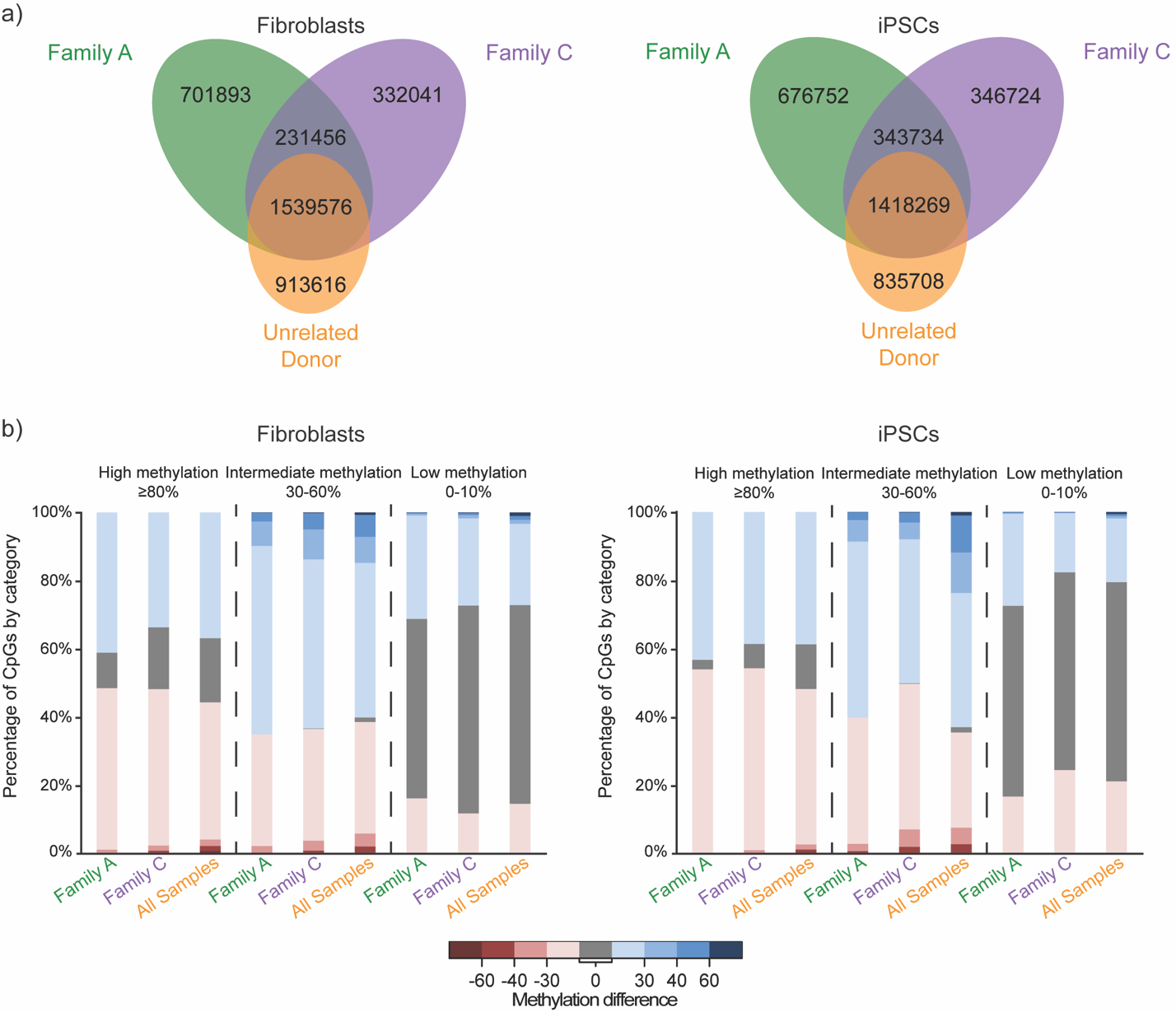
**

**Figure S1: Characterization of DNA methylation in *LMNA­*-mutant fibroblasts and iPSCs. A**, Venn diagrams of the number of overlapping CpGs, captured in RRBS and filtered for ≥ 5x depth across grouped samples for fibroblast (top) and induced pluripotent stem cell (iPSC) (bottom). **B**, Top, Classification of CpGs based on methylation percentage of input control samples (high – left, intermediate – center, or low – right). Middle, Percentage stacked bar plot of CpGs based on the degree of methylation difference (patient-control), as indicated by heatmap legend. Bottom, Group of samples used for percentage calculation.

**
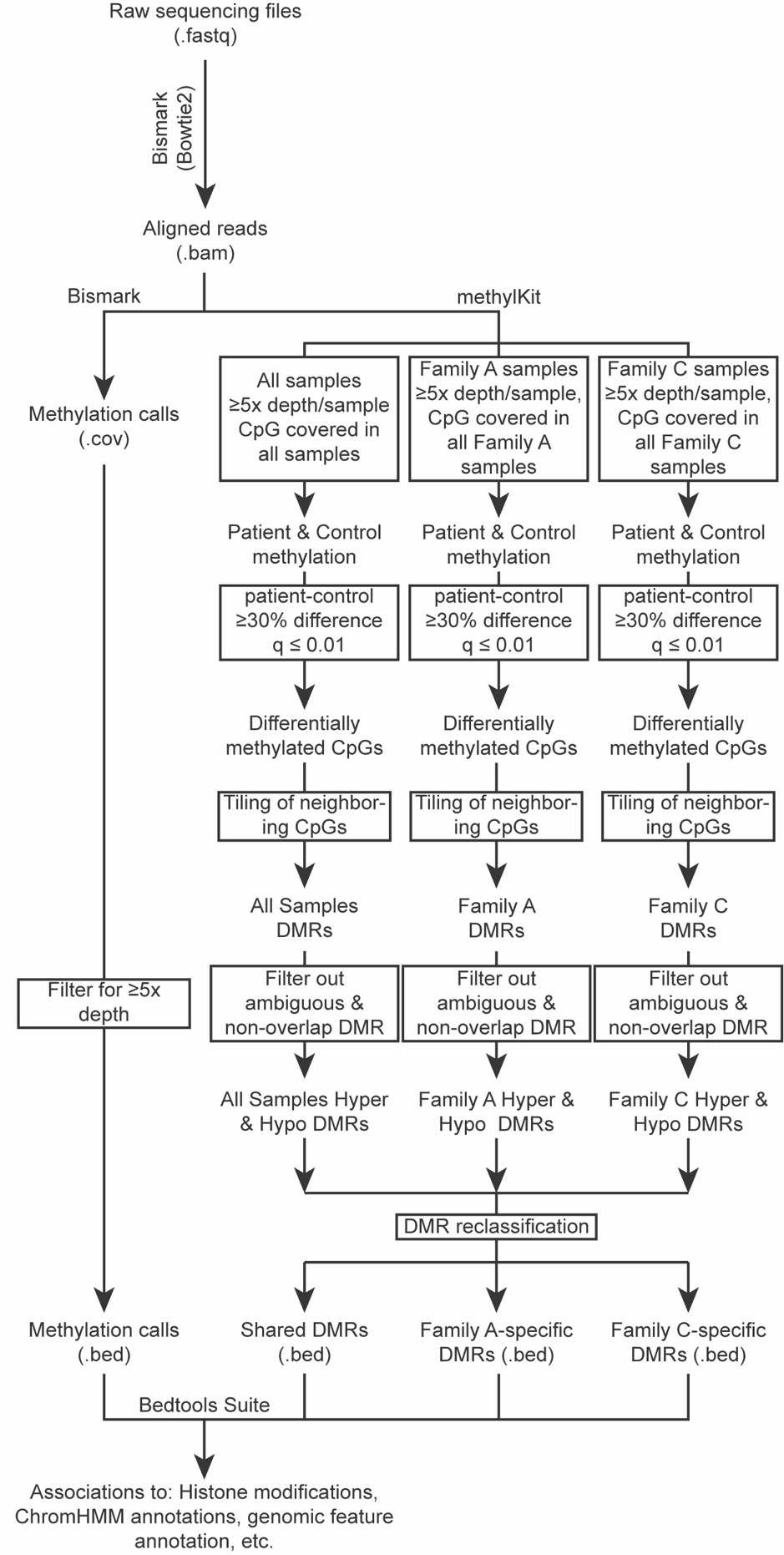
**

**Figure S2: Computational workflow of DNA methylation analyses.** Flowchart showing the computational workflow for producing methylation call and DMR BED files from raw fastq files, and their subsequent analyses.

**
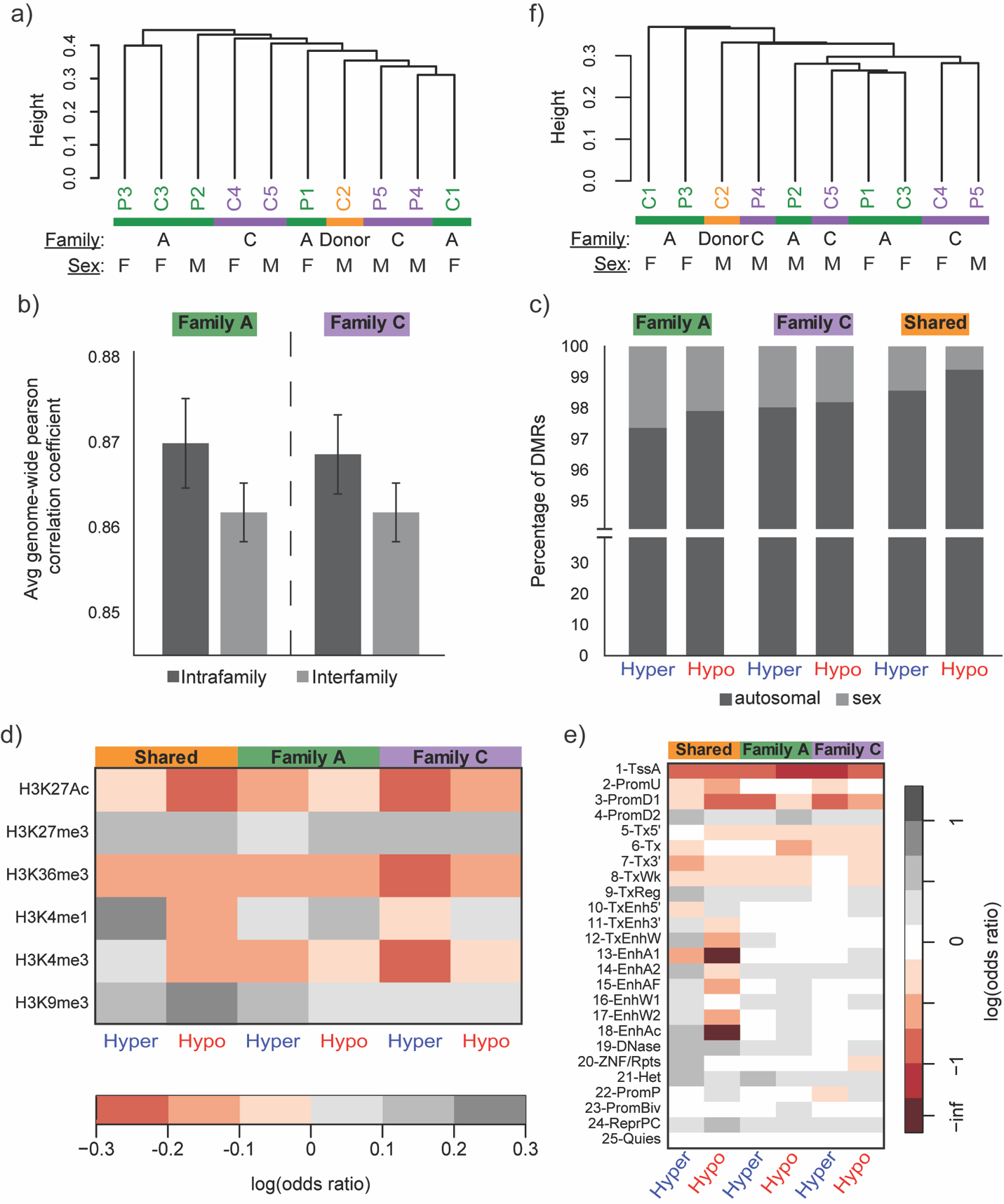
**

**Figure S3: Sex chromosomes had minimal impact on genome-wide and DMR results. A**, Hierarchical clustering of all fibroblast samples by genome-wide DNA methylation of autosomal chromosomes. Colors represent family groupings. **B**, Bar plot of the average Pearson correlation coefficient of genome-wide DNA methylation between fibroblast samples belonging either to the same family (“Intrafamily”) or to the other family (“Interfamily”) in Family A (left) or Family C (right). **C**, Stacked barplot showing the percentage of fibroblast DMRs present either in autosomal or sex chromosomes for hypermethylated (“Hyper”) or hypomethylated (“Hypo”) differentially methylated regions (DMRs) in Family A-specific (left), Family C-specific (center), or Shared (right) groupings. **D**, Heatmap showing the log odds ratio of a CpG falling within both a given histone modification and a fibroblast DMR generated with only autosomal chromosomes. **E**, Heatmap showing the log odds ratio of a CpG falling within both one of 25 ChromHMM annotated genomic regions and a fibroblast DMR generated with only autosomal chromosomes. **F**, Hierarchical clustering of all induced pluripotent stem cell samples by genome-wide DNA methylation of autosomal chromosomes. Colors represent family groupings.

**
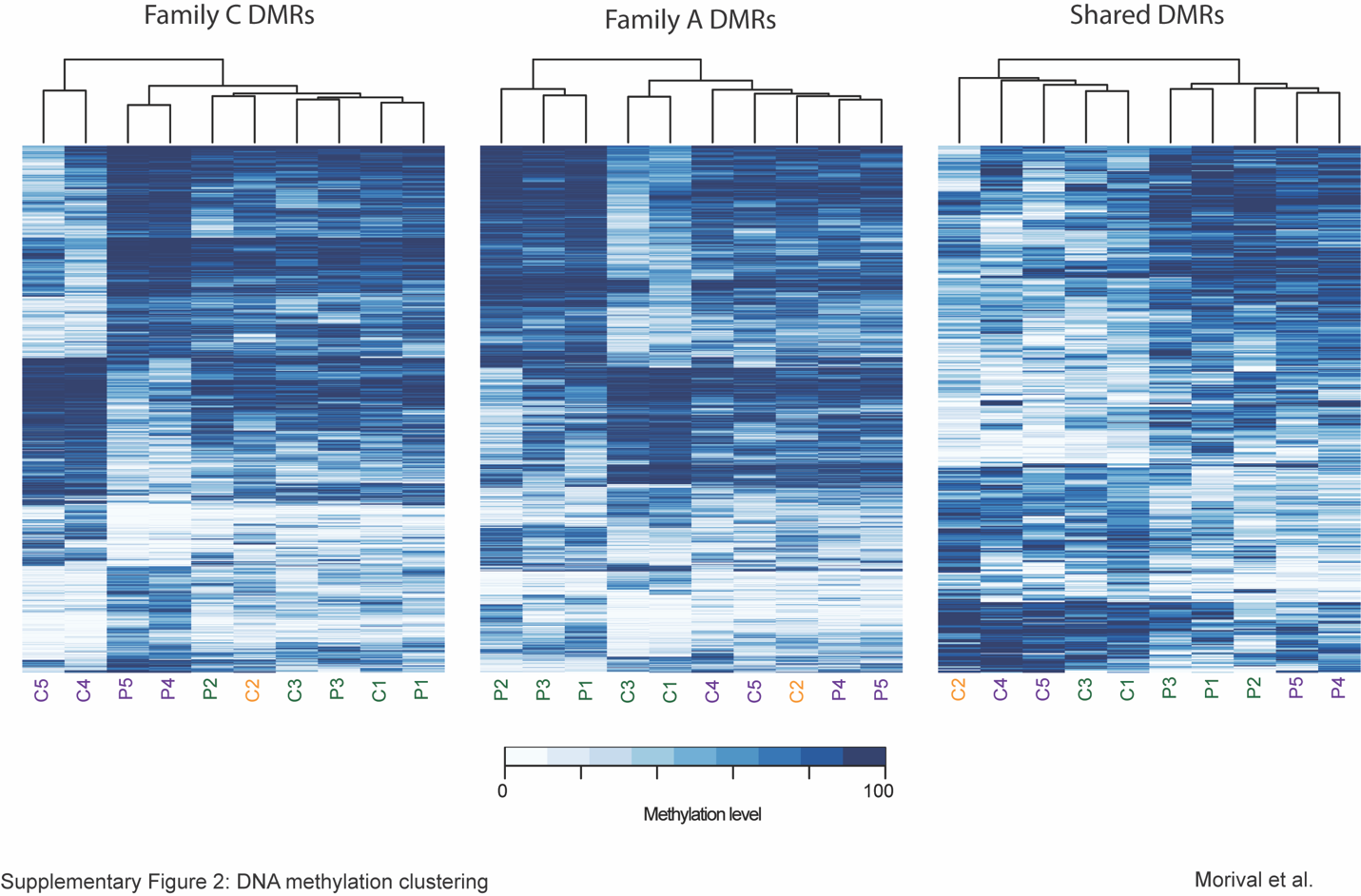
**

**Figure S4: Hypermethylated and hypomethylated DMRs localize at distal regulatory features and transcriptionally repressed chromatin in fibroblasts.** Top, Hierarchical clustering by methylation level in fibroblast samples. Bottom, Heatmap of average CpG (≥ 5x depth) methylation percentage across Family C, Family A, and shared differentially methylated regions (DMRs). Sample IDs are colored based on family of origin: Family C – purple, Family A – green, Unrelated Donor – orange.


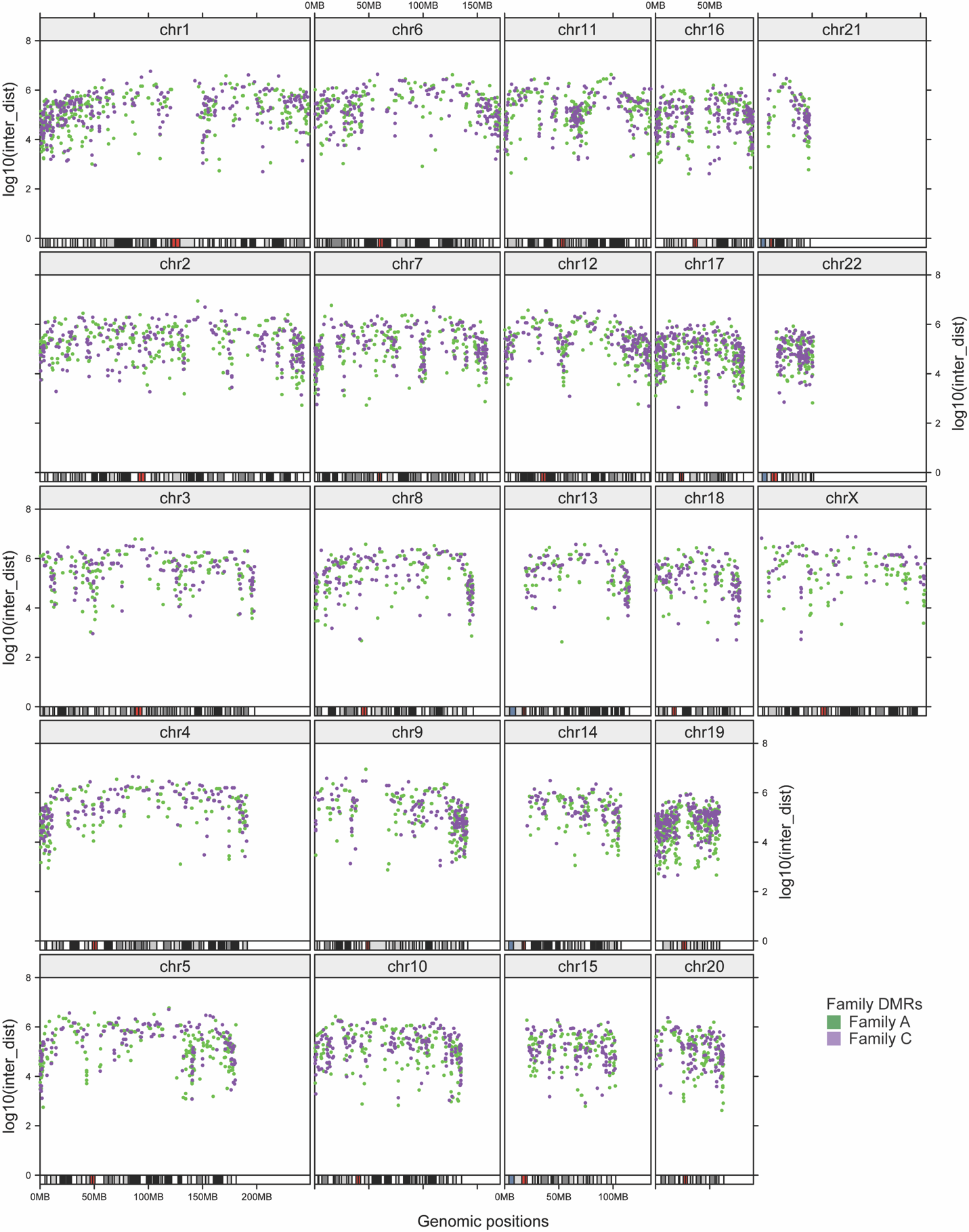


**Figure S5: Inter-DMR distances overlap across both families in all chromosomes.** Top, Trellis rainfall plot showing genomic distance (log10 bp) between DMRs within Family A (green) or Family C (purple) for chromosomes 1 through 22 and X.

**
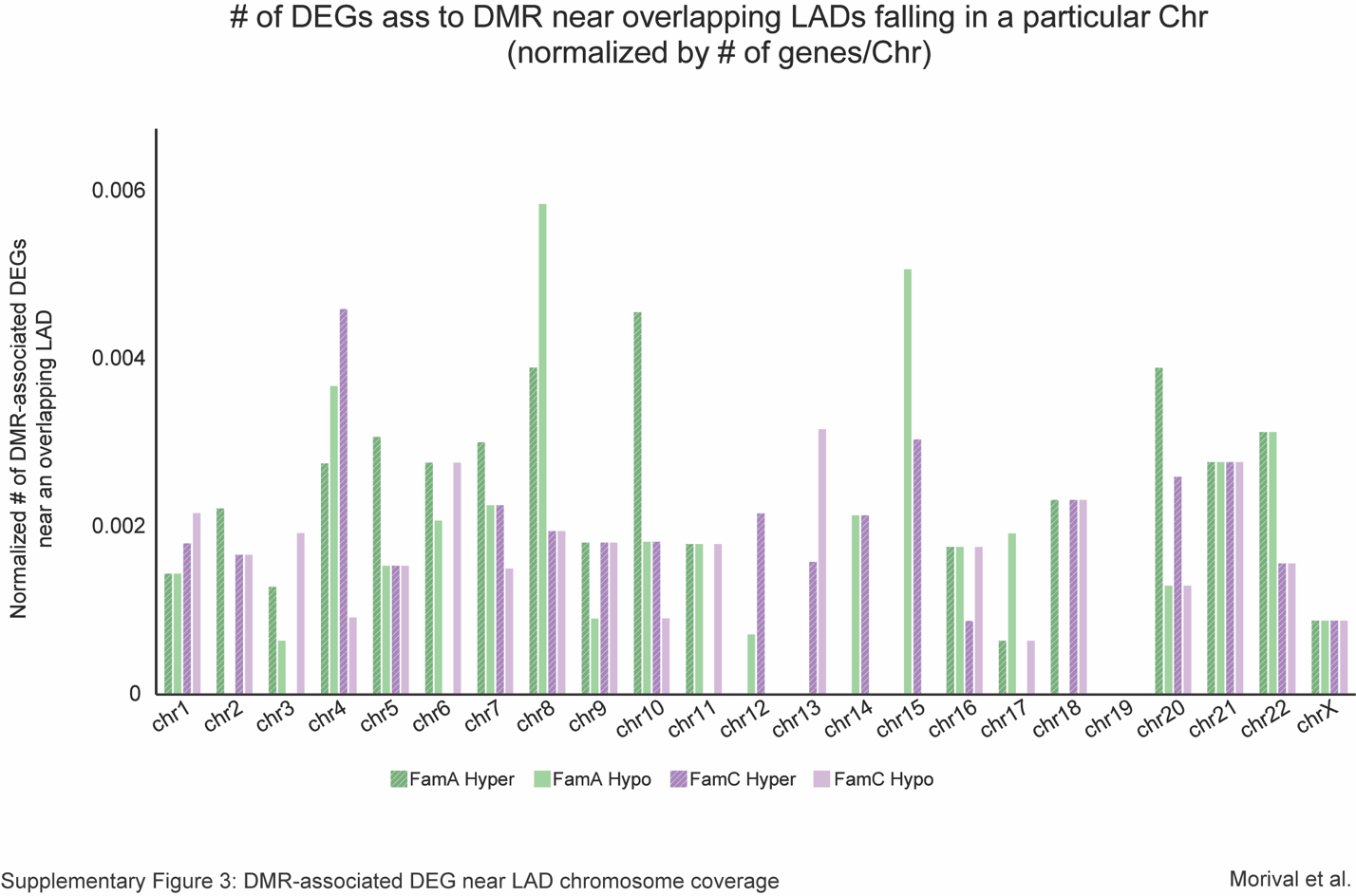
**

**Figure S6: DMRs associate to dysregulated and disease-relevant genes near redistributed LADs.** Bar plot showing the number of DMR-associated differentially expressed genes (DEGs) conserved between fibroblast and cardiomyocyte samples that fall near a redistributed LAD at a particular chromosome. The value is normalized to the number of genes present within the specified chromosome.


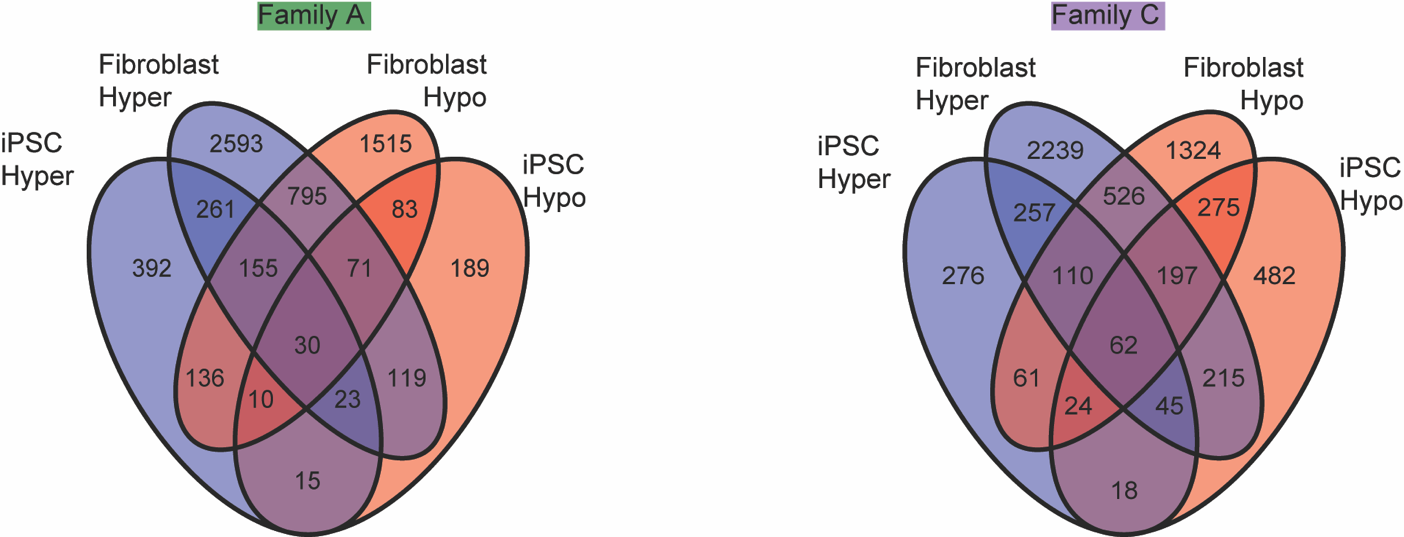


**Figure S7: DMRs in iPSCs reveal tissue-persistent epimutation hotspots at developmentally and laminopathy relevant genes.** Venn diagrams showing the number of genes associated to hyper methylated (blue) and hypomethylated (red) DMRs groups overlapped between both fibroblasts and iPSCs for Family A (left) and Family C (right).


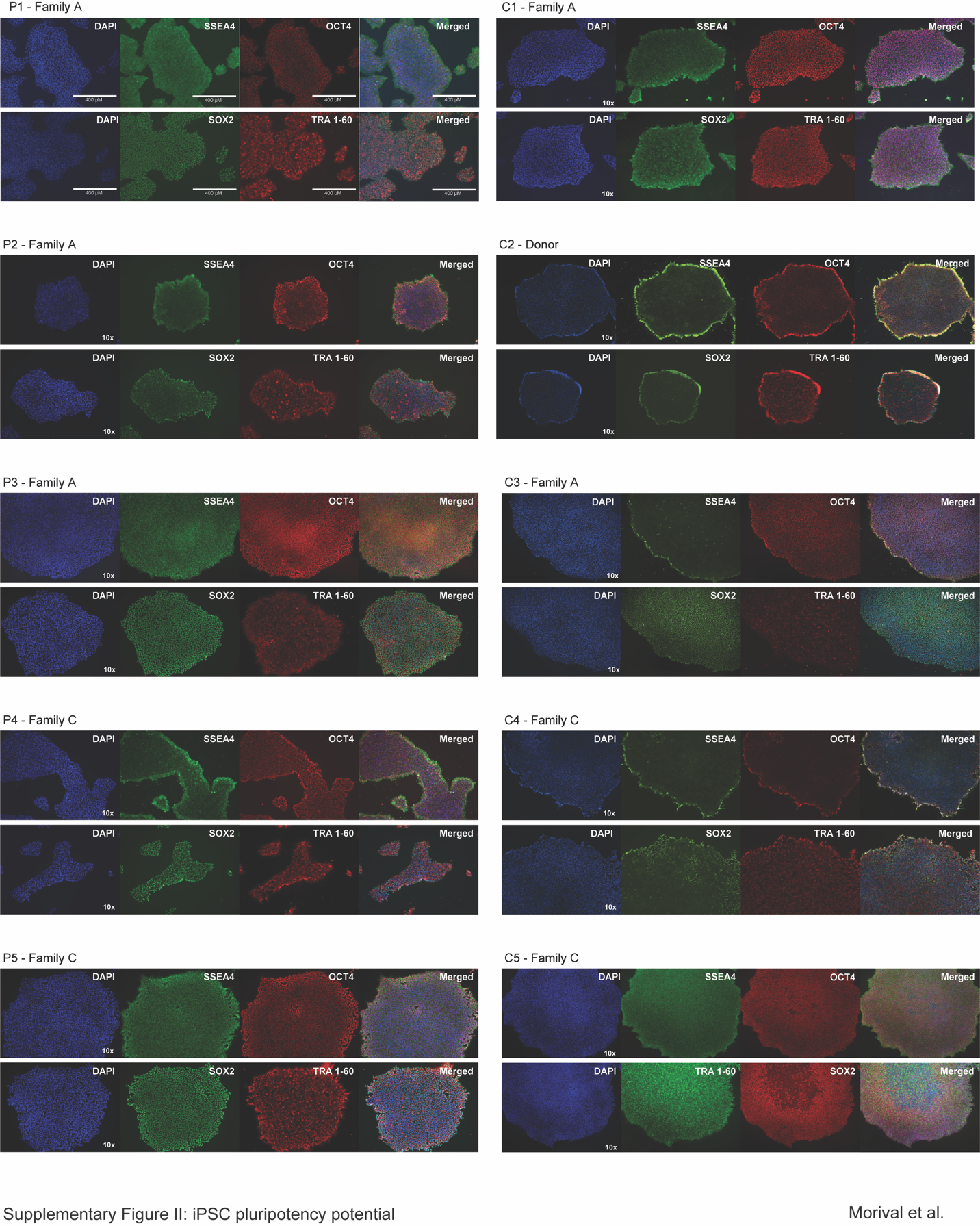


**Figure S8: Validation of induced pluripotent stem cell (iPSC) pluripotency.** Immunocytochemistry staining of all 10 iPSC lines for pluripotent stem cell markers: top, SSEA4 (green) and OCT4 (red) and bottom, SOX2 (green) and TRA 1-60 (red). Nuclei were visualized with DAPI (blue). All images were taken at 10x magnification.


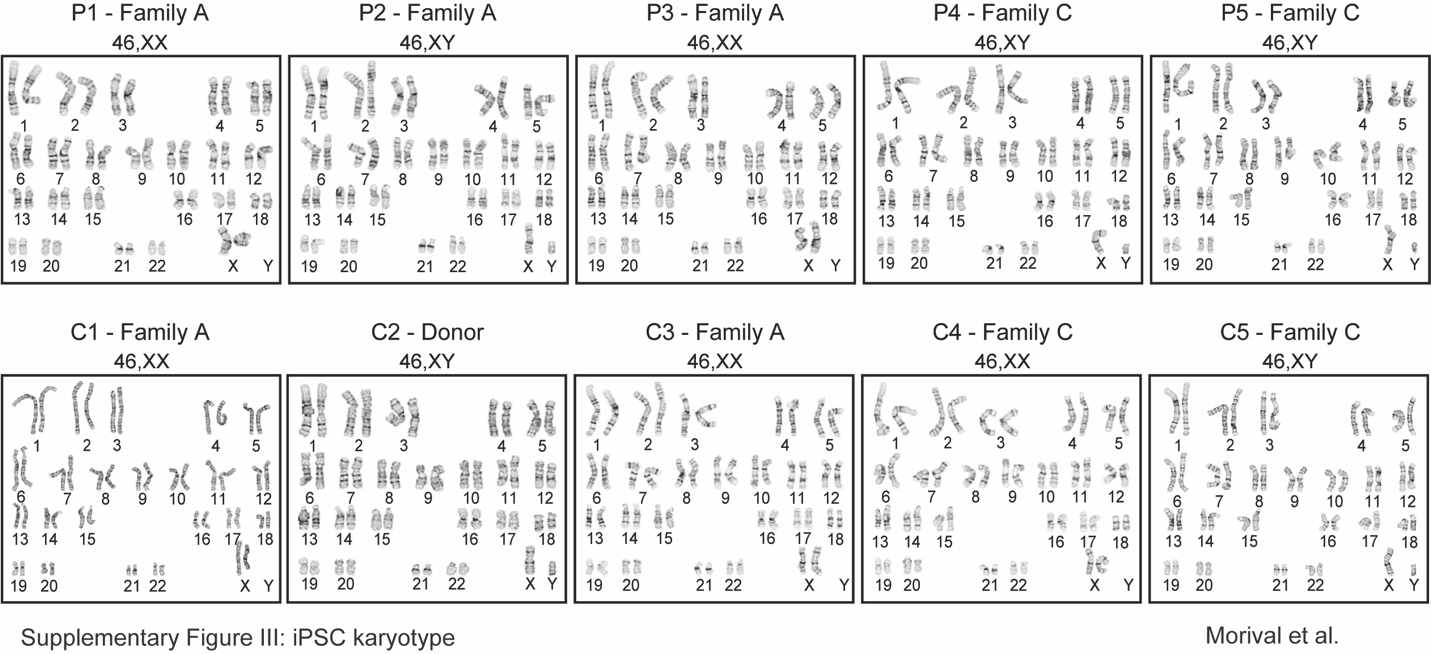


**Figure S9: Validation of normal chromosome constitution in each induced pluripotent stem cell clone.** G-banding metaphase karyotype of all 10 iPSC lines derived from dermal fibroblasts.
